# A Unique Gene Module in *Thermococcales* Archaea Centered on a Hypervariable Protein Containing Immunoglobulin Domains

**DOI:** 10.3389/fmicb.2021.721392

**Published:** 2021-08-18

**Authors:** Kira S. Makarova, Yuri I. Wolf, Svetlana Karamycheva, Eugene V. Koonin

**Affiliations:** National Center for Biotechnology Information, National Library of Medicine, Bethesda, MD, United States

**Keywords:** archaea, *Thermococcales*, hypervariability, gene shuffling, immunoglobulin, polymorphic toxins

## Abstract

Molecular mechanisms involved in biological conflicts and self vs nonself recognition in archaea remain poorly characterized. We apply phylogenomic analysis to identify a hypervariable gene module that is widespread among *Thermococcales*. These loci consist of an upstream gene coding for a large protein containing several immunoglobulin (Ig) domains and unique combinations of downstream genes, some of which also contain Ig domains. In the large Ig domain containing protein, the C-terminal Ig domain sequence is hypervariable, apparently, as a result of recombination between genes from different *Thermococcales*. To reflect the hypervariability, we denote this gene module VARTIG (VARiable *Thermococcales* IG). The overall organization of the VARTIG modules is similar to the organization of Polymorphic Toxin Systems (PTS). Archaeal genomes outside *Thermococcales* encode a variety of Ig domain proteins, but no counterparts to VARTIG and no Ig domains with comparable levels of variability. The specific functions of VARTIG remain unknown but the identified features of this system imply three testable hypotheses: (i) involvement in inter-microbial conflicts analogous to PTS, (ii) role in innate immunity analogous to the vertebrate complement system, and (iii) function in self vs nonself discrimination analogous to the vertebrate Major Histocompatibility Complex. The latter two hypotheses seem to be of particular interest given the apparent analogy to the vertebrate immunity.

## Introduction

Most archaea and bacteria inhabit multispecies environments where they are involved in complex interactions with each other and other community members (Huber et al., 2002; Keller and Surette, 2006; Schopf et al., 2008; Stewart and Franklin, 2008; Nadell et al., 2009; Castro et al., 2016; Flemming and Wuertz, 2019; Imachi et al., 2020). Even in pure cultures, cells interact and, under certain conditions, form biofilms, densely packed microbial communities attached to surfaces and surrounded by extracellular polymers (Lopez et al., 2010; Frols et al., 2012; Jachlewski et al., 2015; Flemming and Wuertz, 2019). In the case of archaea, the current understanding of the key molecular pathways and proteins involved in cell aggregation is limited although archaea known to form complex cell aggregates and biofilms (Chimileski et al., 2014; van Wolferen et al., 2018; Flemming and Wuertz, 2019). Furthermore, relatively little is known about the composition and functions of surface proteins and structures in archaea. Notable exceptions are the archaellum, a motility system, that has been studied in detail and shown to participate in cell adhesion and several other type IV pili structures also shown to be involved in cell adhesion in model organisms (Albers and Jarrell, 2015; Makarova et al., 2016; Chaudhury et al., 2018; Pohlschroder et al., 2018). Even the identity of the major S-layer protein comprising the archaeal cell envelope is known only for several model archaea, whereas the majority of the numerous lipoproteins encoded in archaeal genomes remain completely uncharacterized (Rodrigues-Oliveira et al., 2017; Pohlschroder et al., 2018).

In addition to the widespread biofilm formation, some specialized cell contacts have been described in prokaryotes (Konovalova and Sogaard-Andersen, 2011; Chaudhury et al., 2018; Peterson et al., 2020; Ruhe et al., 2020). In archaea, these include intercellular DNA exchange through different mechanisms, such as conjugation, which is mediated by dedicated systems typically encoded on plasmids that have been characterized, primarily, in Sulfolobales (Stedman et al., 2000; Erauso et al., 2006). Thermococci can form tubular structures with a row of internal vesicles carrying proteins and DNA (Marguet et al., 2013), and Halobacteria form intercellular bridges for mating (Sivabalasarma et al., 2020). Mechanistic details of the formation of such structures in archaea remain poorly understood. It also has been predicted that many archaea possess contact-dependent toxin delivery systems, known as polymorphic toxin systems (PTS) (Makarova et al., 2019), but so far, these have not been experimentally characterized.

*Thermococcales* are hyperthermophilic motile cocci that typically grow in media containing peptides, chitin, starch, or pyruvate, using sulfur or hydrogen as terminal electron acceptors (Rashid and Aslam, 2020). *Termococcus kodakarensis* and *Pyrococcus furiosus* are most commonly used as models (Leigh et al., 2011; Rashid and Aslam, 2020). These organisms are known to form contacts between cells of the same or even different species through their archaeallum filaments (Nather et al., 2006; Schopf et al., 2008).

Only a few surface proteins and structures of *Thermococcales* have been functionally characterized, including the components of archaeallum (Makarova et al., 2016; Daum et al., 2017) and the main S-layer protein (Goda et al., 2018). Altogether, the genome of *T. kodakarensis*, a model organism for this archaeal lineage, encodes 144 proteins (6.3%) predicted to be secreted, most of which remain uncharacterized (Pohlschroder et al., 2018).

We are interested in the functions and evolution of highly variable genes in bacteria and archaea, particularly, those that are subject to frequent horizontal gene transfer (HGT). During the systematic analysis of such genes, we identified a unique, hypervariable gene module in *Thermococcales*, which is centered on immunoglobulin domain-containing proteins predicted to be secreted. We discuss potential functions of this system, which we denote VARTIG (VARiable *Thermococcales* IG), in the light of its remarkable variability.

## Materials and Methods

### Comparative Genomic Framework and Evolutionary Reconstructions

Genome sequences of 526 archaea with complete or nearly complete genomes were downloaded from the NCBI FTP site.^1^ Sequences were assigned to the 2014 arCOGs using PSI-BLAST (Altschul et al., 1997) with the arCOG alignments used to generate position-specific scoring matrices (PSSM) sources as previously described (Makarova et al., 2015). Phyletic patterns, that is, patterns of presence-absence of arCOGs, were derived from the respective arCOGs assignments with a few patterns manually corrected after detailed sequence analysis.

### Construction of COGs for *Thermococcales*

Protein complements of 42 *Thermococcales* genomes were clustered using UCLUST (Edgar, 2010) with sequence similarity threshold of 0.5; sequences within clusters were aligned using MUSCLE (Edgar, 2004). Cluster sequence alignments (as well as singletons) were compared as follows: each alignment was used as a query in a search against the database of alignment consensus sequences using PSI-BLAST (Altschul et al., 1997), with a cut-off *e*-value of 0.0001 (composition-based statistics on, low complexity filtering off). Matches covering at least 75% of the query length were considered full-length; when an alignment with more sequences had a full-length match to a longer alignment with fewer sequences, the longer alignment was cut into segments (putative domains) corresponding to the footprint of the shorter alignment. Alignments that had full-length matches to each other were merged using HHALIGN (Soding et al., 2005). Approximate Maximum Likelihood trees were reconstructed from alignments using FastTree (Price et al., 2010) with the WAG evolutionary model and gamma-distributed site rates; trees were rooted at midpoint. Clades (subtrees), that offer the best trade-off between the representation of species and the number of paralogs, calculated as the value of $S_{C}^{2}/\left(S_{T}\,N_{C}\right)$index (where *N_C* is the number of leaves in a clade and *S_C* and *S_T* are the numbers of species in the clade and in the entire tree, respectively), were iteratively extracted from the tree as clusters of orthologs, and the alignments were partitioned accordingly. The procedure of merging the clusters that produce full-length alignments and splitting clusters with many paralogs was performed iteratively.

### Reconstruction of Gene Gains and Losses

The scaffold phylogenetic tree for *Thermococcales* genomes was obtained from 16S rRNA sequences, aligned using MUSCLE program (Edgar, 2004). Approximate ML phylogenetic tree was constructed using FastTree (Price et al., 2010) (GTR evolutionary model, 20 site rate categories). The GLOOME program (Cohen et al., 2010) with independent gamma-distributed gain and loss rates was used to reconstruct the posterior probabilities for ancestral states for all *Thermococcales* COGs in all internal nodes of the scaffold tree, based on the COG phyletic pattern. Gene gains and losses were inferred from changes in the posterior probabilities (*P*_*descendant*_ - *P*_*ancestral*_ > 0.5 was interpreted as a gain; *P*_*ancestral*_ - *P*_*descendant*_ > 0.5 as a loss). Chromosomal segments of extant genomes with contiguous stretches of genes gained at the terminal branch of the phylogenetic tree, were defined as recently acquired islands.

### Sequence Analysis

Iterative profile searches using PSI-BLAST (Altschul et al., 1997), with a cut-off *e*-value of 0.0001, and composition-based statistics and low complexity filtering turned off, were employed to search for similar sequences in either the NR (non-redundant) database or the protein sequence database of 524 archaeal genomes, unless indicated otherwise in a Supplementary Figure legend. HHsearch was used to identify proteins matching selected profiles from the PFAM database (Soding, 2005). Additionally, other sensitive methods for distant sequence similarity detection were employed including CDD-search (Marchler-Bauer et al., 2009), with cut-off *e*-value of 0.01 and low complexity filtering turned off, and HHpred search with default parameters against PDB, Pfam and CDD profile databases (Zimmermann et al., 2018). Transmembrane helices were predicted using TMHMM v. 2.0c with default parameters (Krogh et al., 2001). Signal peptides were predicted using SignalP v. 5.0 (Almagro Armenteros et al., 2019). Protein secondary structure was predicted using Jpred 4 (Drozdetskiy et al., 2015). Approximate Maximum Likelihood phylogenetic trees were constructed using FastTree with default parameters (Price et al., 2010).

## Results

### Gene Content of Hypervariable, Recently Acquired Loci in Different Lineages of *Thermococcales*

**FIGURE 1:**
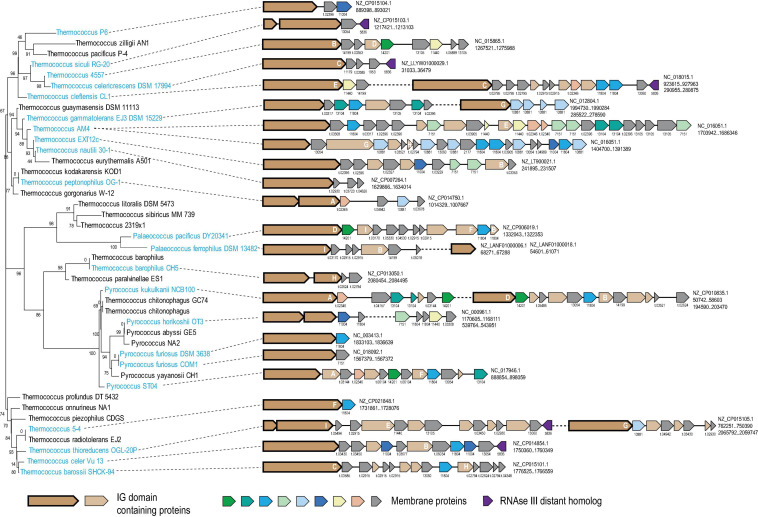
Genomic organization of hypervariable VARTIG loci in *Thermococcales*. The loci are mapped to the 16S rRNA phylogenetic tree of *Thermococcales*. Names of species at the tree leaves where the loci are present are colored light blue. For each gene neighborhood, the genbank accession and coordinates of the locus are indicated on the right. Genes are shown by block arrows, roughly to scale. Genes are colored according to the homology groups (only if found five or more times in the loci) and the key shown at the bottom. Arrows corresponding to gene assigned to arCOG07710 are shown by a thick outline. Cluster number is indicated for all genes, except those coding for Ig-like domain containing proteins. For the genes that are in respective arCOGs the cluster number corresponds to the respective arCOG number. If the proteins are not assigned to any arCOG, the cluster number correspond to Thermococcus specific COGs and indicated with prefix “t.” The letters A-H inside the genes coding for Ig-like domain containing proteins correspond to the respective branches in the tree built for the C-terminal region of these proteins (Supplementary Figure 2). Detailed information on these neighborhoods is available in Supplementary Table 1.

We explored large genomic islands that appeared to have been recently acquired by different lineages of *Thermococcales* using information on the point of gene acquisition during the evolution of this group of archaea (see section “Materials and Methods” for details). Two such genomic islands containing more than 20 genes each were identified in the genome of *Thermococcus sp.* AM4 (TAM4_RS07620-TAM4_RS07720 and TAM4_RS09285- TAM4_RS09405) and were predicted to have been acquired by this organism only (Supplementary Table 1). These two islands did not share any genes from same Thermococci-specific COGs, but both encoded proteins of arCOG07710 and arCOG14200, initially annotated as “Cell surface protein” and “Uncharacterized protein,” respectively. Both these arCOGs are represented in *Thermococcales* only (Supplementary Table 2). Further examination of the neighborhoods of arCOG07710 and arCOG14200 revealed 31 islands in 22 of the 42 analyzed thermococcal genomes. Almost all genes in these islands were predicted to be acquired at the respective terminal branches, with the exception of two strains of *P. furiosus*, where acquisition of such an island was mapped to the common ancestor of these strains (Supplementary Tables 1, 2). The length of the islands varied from 1 to 25 genes (Figure 1). Altogether, these islands contained 239 genes assigned to 120 thermococcal COGs including singletons, 68 of which are represented only in a single genome (Supplementary Table 2). None of these genes was detected in any genome location other than these islands, suggesting that they all belong to the same functional system. The arCOG07710 proteins are most common, being represented in 28 islands. However, the arCOG07710 proteins were assigned to 17 thermococcal COGs because, due to their extreme divergence, they were not merged in a single COG by our procedure and because several of the respective genes appear to be fragments of pseudogenes (Figure 1 and Supplementary Table 1). Of the 120 thermococcal COGs represented in these islands, 82 consist of proteins predicted to contain one or more transmembrane segments, and in three more COGs, at least half of the proteins contain predicted signal peptides (Supplementary Table 1). The arCOG07710 proteins are always encoded at the 5′ end of a putative operon, followed by genes coding for smaller proteins, most of which contain predicted transmembrane segments (Figure 1). These observations strongly suggest that the putative functional system encoded in the islands is membrane-associated, and that its activity involves protein secretion.

### Sequence Analysis of Proteins From the Hypervariable Islands

Given that the proteins encoded in the identified variable islands apparently evolve fast, we ran PSI-BLAST for up to three iterations against proteins from the arCOG database using a representative of each thermococcal COG as the query to determine whether some of these proteins could be further combined into broader groups of homologs. These searches allowed us to delineate 44 homology groups (Supplementary Table 2). The largest group combined members of arCOG07710 and arCOG14200, and a variety of other proteins encoded in the islands. Most of the arCOG07710 members are large proteins (>500 aa), whereas arCOG14200 members and other proteins of this homology group are smaller and align with the C-terminal region of the arCOG07710 protein (Supplementary Table 1 and Supplementary Figure 1A). A signal peptide was predicted for 19 of the 30 arCOG07710 proteins, and for 8 proteins from this arCOG, a transmembrane domain was predicted at the C-terminus (Supplementary Table 1 and Supplementary Figure 1A).

**FIGURE 2:**
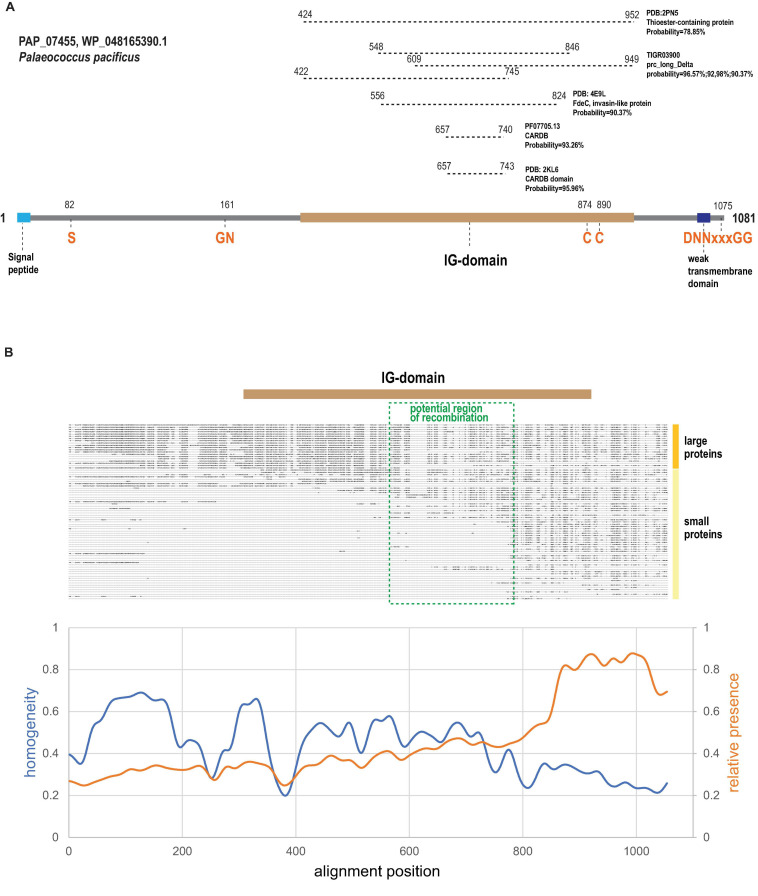
Domain organization, sequence motifs and C-terminal variability of Ig-domain containing proteins encoded in VARTIG loci. **(A)** Domains and sequence motifs discussed in the text are mapped on a representative protein of arCOG07710. Coordinates of the motifs are indicated. Regions of similarity to several Ig-domain containing proteins or profiles identified using HHpred are indicated above the domain scheme. Coordinates for the respective regions are indicated. **(B)** Schematic multiple alignment of Ig domain-containing proteins (Supplementary Figure 1) and sequence conservation plot. The Ig region and the potential area of recombination are mapped to the alignment. The smoothed curves show the fraction of sequences in the corresponding position of the alignment (orange, calculated for sequences shorter than 700 amino acids) and the alignment homogeneity (Esterman et al., 2021) (blue, calculated for sequences longer than 700 amino acids; homogeneity of 1 corresponds to perfect conservation, homogeneity of 0 corresponds to a random amino acid mixture).

All strong matches (probability >90%) detected by HHpred were confined to the C-terminal half of the large arCOG07710 proteins, which aligns with other members of this homology group, as exemplified by PAP_07455 (WP_048165390.1) protein from *Palaeococcus pacificus* DY20341 (Figure 2A). In particular, the bacterial cell adhesion related domain CARDB (PF07705.13) was identified as a homolog (Figure 2A). CARDB belongs to PFAM clan CL0159 termed the ‘‘early’’ (E)-set and includes multiple protein families with the immunoglobulin (Ig) fold found in bacteria, archaea and eukaryotes, including fibronectin type-III, PKD repeats, bacterial Ig-like domains, and many others^2^ (Bork et al., 1994). Furthermore, a structure of a closely related single CARDB domain was solved for another cell surface protein from *Thermococcales* (PDB: 2KL6; PF1109 from *P. furiosus*). However, these proteins (arCOG07086) are encoded neither in the variable islands described here (hereafter VARTIG islands or loci) nor in any other identified variable loci although our reconstructions indicate a relatively high HGT propensity for these genes (Supplementary Table 2). The region from 556 to 824 amino acid (here and after mapped to WP_048165390.1) positions corresponds to the PDB: 4E9L (invasin-like protein FdeC) profile and, more specifically, to region B in FdeC (Nesta et al., 2012; Figure 2A). In the FdeC structure, region B accommodates three Ig-like domains, and the CARDB similarity region roughly corresponds to a single Ig unit. The TIGR03900, carboxyl-terminal-processing protease, profile can be mapped to the same region of arCOG07710 as FdeC but also displays similarity to two more regions covering 527 amino acids altogether (Figure 2A). The longest region of similarity corresponds to PDB:2PN5, insect thioester-containing protein (TEP) (Figure 2A), which contains five Ig domains (Baxter et al., 2007). Thus, we predict that arCOG07710 proteins contain five Ig-like domains that cover the region from 422 to 952 amino acid positions (Figure 2A).

We next examined the multiple alignment of the arCOG07710 proteins to identify any distinct shared features and detected a highly conserved N-terminal region containing a GN motif located close to the Ig domain (Figure 2A and Supplementary Figure 1A). Considering parallels with PTS and other surface proteins involved in immunity, such as bacteriocins and archaeocins, the GN motif might comprise a protease cleavage site resembling several known cleavage sites that all include a glycine residue (Dirix et al., 2004; Shen, 2010; Makarova et al., 2019). Many polymorphic toxins contain a protease domain that is involved in the protein maturation (Prochazkova et al., 2009; Shen, 2010). Depending on the type of the protease, conserved histidines, cysteines, aspartates and/or serines that are directly involved in catalysis can be found in such protease domains. Thus, we checked the conserved region for the presence of such residues. The only strictly conserved residue we identified was a serine, suggesting that these proteins might contain a serine endoprotease (Figure 2A and Supplementary Figure 1A).

Next, we noticed two conserved cysteines in the region corresponding to the most distal Ig-domain (Figure 2A and Supplementary Figure 1A). In extracellular proteins including the Ig domains, cysteines often form disulfide bonds that additionally stabilize the mature protein (Wiedemann et al., 2020). We further noticed that 31 arCOG07710 proteins and smaller homologous proteins containing predicted Ig domains contained a DNNxxxGG (where x is any amino acid) motif at the C-terminal end (Figure 2A and Supplementary Figure 1A). This motif is often preceded by a hydrophobic region that in some cases is predicted to form a transmembrane segment. A short C-terminal conserved motif preceded by a hydrophobic region might be a recognition signal for a sortase, a dedicated enzyme that covalently attaches proteins to the cell surface, although this particular signal has not been described previously (Haft et al., 2012; Malik and Kim, 2019). All known sortases are membrane transpeptidases with a catalytic triad that includes an active cysteine, typically, along with a histidine and an asparagine (Haft et al., 2012; Malik and Kim, 2019). Sortases are often encoded in the immediate vicinity of the protein containing the cognate signal because most are specific for only a few or even a single target (Haft et al., 2012). Because we did not find the D[ND]NxxxG[GD] (x is any amino acid) motif in the 15 C-terminal amino acids of any other proteins of *Thermococcales*, it could be expected that a dedicated sortase (if any) would be encoded in the same loci. However, we failed to identify any protein family encoded within these loci that would possess a conserved cysteine along with the other conserved residues that might contribute to the catalytic triad of a sortase. The possibility remains that arCOG07710 proteins are processed by a sortase that is encoded elsewhere in the thermococcal genomes. Proteins of one small family (arCOG14201) represented in the variable islands contained several conserved histidines suggesting that these could be metalloenzymes (Supplementary Figure 1B). The function of these putative enzymes remains unclear.

### Evidence of Frequent Recombination in the C-Terminal Ig Domain-Containing Region of arCOG07710 Proteins

Examination of the alignment of the Ig-domain containing proteins from VARTIG (Figure 2B) showed that the smaller proteins aligned with the C-terminal region of the larger (arCOG07710) proteins. The plot of amino acid residue conservation for the shorter proteins shows a prominent plateau corresponding to the last 200 amino acids of the longer proteins (Figure 2B). This region contains the most distal Ig domain (∼100 aa), including conserved cysteines and the C-terminal region with the putative sorting signal. In contrast, the conservation plot for the large proteins only (20 proteins >700 aa) shows that this C-terminal region is the most variable portion of these proteins (Figure 2B). The likely explanation of such diversity is frequent recombination generating many variants of the distal Ig domain, which remains in the putative mature protein after the cleavage of the sorting signal.

To further analyze this apparent recombination, we constructed a phylogenetic tree for the ∼200 aa C-terminal region of arCOG07710 only and mapped 9 well-supported branches (A–I) each consisting of closely similar sequences on the respective gene neighborhoods (Figure 1 and Supplementary Figure 2). This mapping showed that the level of sequence conservation did not follow the species tree, with the most similar sequences scattered among the tree branches. No indications of recombination within the loci were detected. Instead, according to the species tree, all exchanges occurred between different species, at least on several occasions, *in situ*, next to a gene encoding a distant homolog of RNAse III (arCOG05836) (Figure 1). This observation strongly suggests that the smaller genes recombine with the long genes in apparently random combinations and that this shuffling might occur during DNA exchange between cells from different populations or even species.

Given the hypervariability of the C-terminal Ig domain of the arCOG07710 proteins, we denoted this gene module VARTIG (after VARiable *Thermococcales* IG). Although we did not detect intra-gene recombination events in other gene families in the VARTIG loci, the fact that all these loci have different gene arrangements (Figure 1) strongly suggests that they undergo extensive shuffling during the evolution of *Thermococcales*.

### Search for Analogous, Hypervariable Gene Modules in Other Archaea

In order to test for the presence of analogous, hypervariable systems in other archaea, we employed three distinct strategies. First, we performed a PSI-BLAST search against the arCOG database using the sequence of PAP_07455 (WP_048165390.1) protein from *P. pacificus*, a typical arCOG07710 member, as the query. This search converged after 5 iterations and identified, in addition to the homologous proteins from *Thermococcales*, 139 proteins from other groups of archaea, mostly Halobacteria (83) and Methanomicrobia (45) (Supplementary Table 3). According to the automatic arCOG assignments, these proteins possess 110 distinct domain architectures, the most frequent one (combination of arCOGs 2540, 3256, and 7560) identified in only 9 proteins from Halobacteria (e.g., WP_082230105.1). These are large (predicted) secreted proteins containing several CARDB domains. The arCOGs identified most frequently in these proteins are arCOG07560, multidomain secreted protein, often associated with PTS (71), and arCOG02508, secreted proteins, containing PKD repeats (68). The genomic neighborhoods (5 genes upstream and downstream) of these genes were examined in an attempt to identify variable loci that might have similar organization to VARTIG but would be characteristic of other major archaeal lineages (Supplementary Table 3).

In the second approach, we examined the neighborhoods (five genes upstream and downstream) of genes encoding CARDB domain-containing proteins identified by similarity to the respective PFAM profile (pfam07705). Altogether, we analyzed 858 neighborhoods of genes encoding 914 non-redundant CARDB-containing proteins (Supplementary Table 3). According to the automatic arCOG assignments, these proteins display 489 distinct domain architectures, the most frequent one being a single CARDB domain containing protein (arCOG02532).

The third strategy was to search for any Ig-domain containing proteins encoded in a close vicinity of each other. To this end, we used assignments of all proteins from the arCOGs to the 238 PFAMs profiles within the E-set clan (see text footnote 2, Supplementary Table 3). We identified those loci that encoded two or more Ig-containing proteins separated by no more than five other genes (Supplementary Table 3). Altogether, we visually examined 38 sets of such loci, in search for organizations similar to that of VARTIG. In particular, we searched for loci specific to a major archaeal lineage and encoding a large Ig-domain containing protein (1000 aa or more) with a variable C-terminal region, followed by a variable number of other genes, at least one of which would also encode an Ig-domain containing protein.

Neither of these approaches identified any gene modules closely resembling VARTIG in other archaea. However, we detected many loci encoding several large Ig-domain containing proteins, often with different but overlapping domain organizations (Supplementary Table 3). In particular, Methanomicrobiales encompass numerous loci that consist of multiple genes encoding putative surface proteins containing pfam18911, PKD repeats of the Ig fold (for example, WP_011024165.1-WP_011024196.1 in *Methanosarcina acetivorans* C2A). It could be expected that genes in such loci would frequently recombine, resulting in new domain combinations and/or mosaic protein containing several domains of different origins. Indeed, we identified several examples of apparent mosaic proteins in Methanosarcinales (Supplementary Figure 3). Unlike the case of VARTIG, however, in many of these loci, the C-terminal Ig domain is either conserved or homogenized by recombination.

### Archaeal Gene Neighborhoods Encoding α2-Macroglobulin Homologs

**FIGURE 3:**
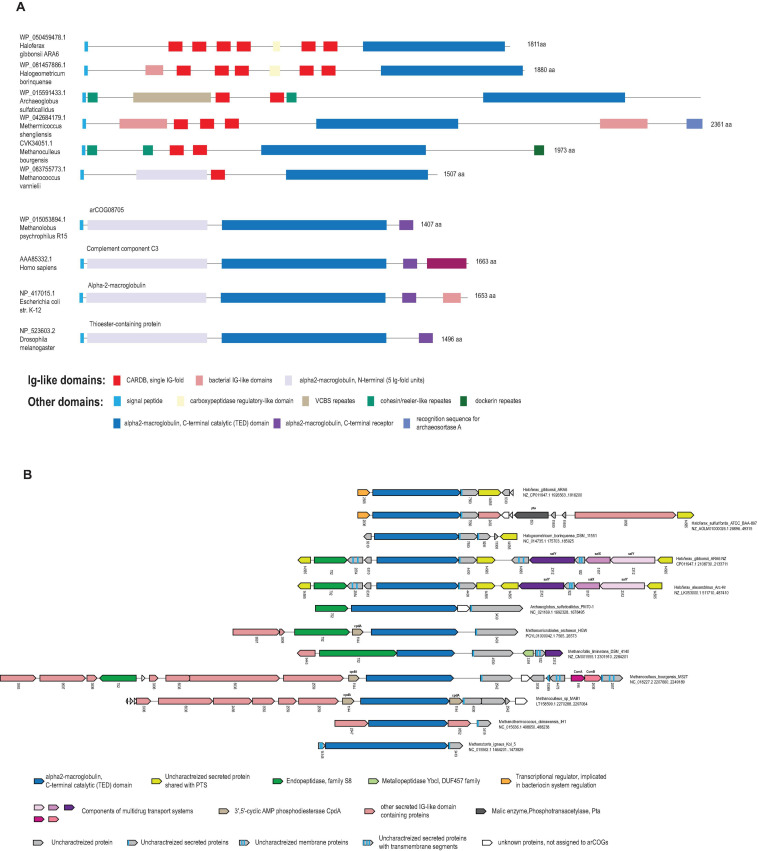
Alpha 2-macroglobulin homologs in archaea. **(A)** The domain organizations of selected alpha 2-macroglobulin homologs. Domains are shown by colored rectangles according to the color key shown beneath the protein schematics. The length of the domain scheme and the domain boundaries are shown roughly to scale. For each protein, the GenBank accession and the species name are indicated. The length of each protein is indicated on the right. **(B)** Selected neighborhoods of genes coding for alpha 2-macroglobulin homologs. Designations are mostly the same as in the Figure 1. The arCOG numbers are indicated below the respective arrows. Gene names are indicated for a few known protein families. The color code is shown underneath the schematics.

Considering the pronounced similarity of the domain organizations and sequences between the Ig domain-containing regions of the arCOG07710 proteins and TEP, several distant homologs from other archaea appeared of interest. Specifically, we identified a group of archaeal proteins with an even greater similarity to TEPs and other α2-macroglobulin family members from several arCOGs (Supplementary Tables 2, 3) that are encoded in archaeal genomic neighborhoods sharing genes with PTS (Makarova et al., 2019). Several of these proteins, primarily in Halobacteria and Methanomicrobia, were picked up by our third search strategy because they are encoded in the immediate vicinity of other Ig domain-containing proteins (Figure 3A and Supplementary Table 3). Sequence analysis of these proteins (for example, WP_050459478.1 from *Haloferax gibbonsii* ARA6) showed that, in addition to the Ig-domain, they contain the thioester-containing alpha helical domain (TED), in an arrangement similar to TEPs and alpha 2-macroglobulins (Figure 3A). Furthermore, these archaeal proteins contain the motif “CxEQ,” where the catalytic cysteine forms a covalent bond with the target (Blandin and Levashina, 2004; Baxter et al., 2007; Rehman et al., 2013).

Next, we searched the arCOG database using PSI-BLAST and the TED domain from WP_050459478.1 as a query and identified the TED domain with intact “CxEQ” motif in 27 proteins from 24 archaeal genomes (Supplementary Figure 1C and Supplementary Tables 2, 3). Several proteins from Methanomicrobia are closely related to bacterial α2-macroglobulin homologs (Garcia-Ferrer et al., 2015), for example, WP_015053894.1 from *Methanolobus psychrophilus*, and thus, most likely, were acquired from bacteria via HGT (Figure 3A). The remaining α2-macroglobulin homologs are specific to archaea and typically contain a predicted signal peptide, but otherwise, have different domain organizations in different archaea (Figure 3A). In some archaeal genomes, genes encoding α2-macroglobulin homologs are located in complex neighborhoods along with other putative surface proteins, some of which contain Ig domains, antimicrobial peptide transporters, endoproteases and uncharacterized proteins. These loci are not as diverse as the VARTIG loci, and we found no evidence of shuffling in the C-terminal regions of these proteins (Figure 3B and Supplementary Table 2). Notably, however, in Halobacteria, the α2-macroglobulin domain-containing genes are often followed by genes of arCOG14565 encoding an uncharacterized protein that is also encoded next to many PTS (Makarova et al., 2019; Figure 3B and Supplementary Table 3). Additionally, the transcriptional regulator from arCOG02808 associated with Halobacterial alpha 2-macroglobulin modules were detected in the vicinity of genes encoding archaeocins (Makarova et al., 2019). Taken together, these observations suggest that archaeal immune systems and systems involved in inter-microbial conflicts share components and, potentially, the mechanisms for active molecule delivery and domain shuffling.

## Discussion

Here, we describe the VARTIG modules that are unique to *Thermococcales* and consist of genes encoding multiple, highly variable Ig domain-containing proteins along with heterogeneous sets of additional proteins. We obtained strong indications of recombination occurring in the C-terminal region of the large VARTIG protein resulting in its extreme variability. Surprisingly, recombination appears to occur not between different VARTIG loci within the same genome, but rather, between loci from different strains and species of Thermococci. In addition, the VARTIG loci seem to be subject to extensive shuffling. Recombination and rearrangement in the VARTIG loci likely represent dedicated diversification mechanisms that are, in principle, analogous to the intragene rearrangements that occur in genes encoding various surface proteins in bacteria and archaea, such as PTS (Hill et al., 1994; Makarova et al., 2019), adhesins (Martin-Galiano, 2017), and virulence factors (Dashper et al., 2017). Another, perhaps distant, but relevant analogy is the diversification of Ig in the vertebrate immune system that occurs via a combination of hypermutation and V(D)J recombination (Schatz and Swanson, 2011). However, diversification via inter-strain or even inter-species recombination appears to be a unique feature of VARTIG that implies the possibility of community-level adaptation.

The molecular mechanisms of recombination and rearrangement in the VARTIG loci remain unclear. There are not enough closely related genomes of *Thermococcales* currently available to align the nucleotide sequences of the genes coding for long Ig domain containing proteins and identify recombination sites precisely, especially, because these sites are likely spread across a region of about 600 nucleotides (Figure 2B). These genes seem to be too far diverged for homologous recombination, and the other genes in the VARTIG loci are even more variable. It is unclear if any integrase-like proteins are involved in VARTIG shuffling in a manner similar to the phase variation mechanism of restriction-modification systems (De Ste Croix et al., 2017), but no identifiable integrases are encoded in the VARTIG neighborhoods. Considering the parallels in the organizations of the VARTIG loci and PTS, it appears likely that the mechanisms of shuffling are similar as well. Apart from direct experimental study, genome sequencing of closely related strains should at least allow the identification of the sequence features promoting recombination.

The function of VARTIG remains enigmatic. Considering the patchy distribution and variability of VARTIG in *Thermococcales*, it is highly unlikely to be essential. We could not identify any consistent difference between the lifestyles of those Thermococci that encode VARTIG modules and those that lack them, at least, with the current state of the knowledge of the biology of these archaea. This pattern of phyletic distribution, and especially, the extensive diversification seems to be compatible with the involvement of VARTIG in inter-microbial conflicts (Aravind et al., 2012). Again, parallels with PTS are prominent. Both types of systems typically encompass a long protein encoded by the first gene in an array of co-directed genes. In PTS, the gene arrays include a variable number of genes encoding proteins homologous to the C-terminal regions of the longer protein that encompass diverse toxin domains (Zhang et al., 2012; Makarova et al., 2019). These loci also often encode immunity proteins, and typically, a dedicated immunity protein is encoded in the immediate vicinity of the respective toxin (Zhang et al., 2012; Makarova et al., 2019). A similar trend was observed in the VARTIG modules: genes encoding a distinct set of (predicted) membrane proteins often follow the same subfamily of genes coding for Ig domain proteins (Figure 1). For example, membrane proteins from cluster 14199 are encoded next to Ig domain protein from branch B, whereas membrane proteins from cluster 11440 are encoded next to a Ig domain protein from branch E. Furthermore, PTS containing Ig domains within the effector protein also have been identified in archaea (Figure 3; Makarova et al., 2019). The major caveat of the hypothesis that VARTIG is a distinct variety of PTS is that we could not identify any potential toxins, in particular, DNase or RNase domains, which are the most common toxin moieties in archaeal PTS (Makarova et al., 2019).

The second hypothesis is that VARTIG is an innate immunity system. The similarity between the Ig domain-containing proteins of VARTIG and the Ig-like N-terminal domain of TEP and complement components, which are a key animal innate immunity systems (Hajishengallis et al., 2017; Shokal and Eleftherianos, 2017), is best compatible with this hypothesis (Figure 2A). Although VARTIG proteins do not contain TED domains, the conserved cysteines potentially could be involved in the formation of covalent bonds with the targets. Thus, considering the diversity of the C-terminal region of the large Ig domain-containing protein, VARTIG might be analogous to the vertebrate complement factors that can bind different targets (Ricklin et al., 2016). The identification of TEP homologs in archaea and the fact that they also share some features with PTS further suggest that archaea evolved immune systems that generate an extensive repertoire of immunity proteins via gene shuffling and recombination (Figure 3A). An intriguing possibility is that the α2-macroglobulin domain-containing module is ancestral to VARTIG, which evolved into a more sophisticated complement-like innate immunity system capable of targeting many different antimicrobial molecules.

The third possibility is that VARTIG is involved in cell-cell adhesion and/or self-nonself recognition. This hypothesis is based on the fact that many surface proteins in bacteria and eukaryotes are known to be involved in cellular aggregation or in attachment to the host cells, in the case of parasites (Trunk et al., 2018; Honig and Shapiro, 2020). In particular, the Ig domain of PAP_07455 protein shows a highly significant sequence similarity with *E. coli* FdeC, the adherence factor which mediates *E. coli* adhesion to mammalian cells (Nesta et al., 2012; Figure 2). In archaea, so far, only type IV pili have been shown to be involved in cell-cell adhesion (Chaudhury et al., 2018; Pohlschroder et al., 2018; and references therein). Recently, it has been shown that UV-inducible type IV pili in *Sulfolobus acidocaldarius* mediate formation of cellular aggregates, ensuring specific self vs nonself recognition (van Wolferen et al., 2020). This specificity is primarily determined by the variable region of the major pilin UpsA (van Wolferen et al., 2020). Notably, archaeal type IV major pilins contain Ig-fold domains (Braun et al., 2016) that are highly variable and are often encoded in multiple copies in some archaeal genomes (Makarova et al., 2016). Another thoroughly characterized case of the involvement of Ig domain in self vs nonself discrimination are the major histocompatibility complex (MHC) proteins in vertebrates (Wieczorek et al., 2017). Numerous allelic variants of MHC (more than 200 in humans) recombine in meiosis producing a unique combination of alleles for each individual (Abualrous et al., 2021). Each MHC molecule displays an epitope, a small peptide derived from a degraded protein on the cell surface. The presented peptides derived from self proteins prevent the immune system from self-targeting. This specificity is achieved during the early development of the T lymphocytes, which are selected to recognize self MHC molecules, but not any self antigens. The MHC also appears to affect the mate choice (Boehm and Zufall, 2006). The predicted ability of VARTIG to generate numerous genetic variants of the surface domains (“epitopes”), possibly, by intragenic shuffling during DNA exchange, is compatible with the hypothesis that VARTIG is involved in self vs nonself recognition, and perhaps, in the mate choice for DNA exchange.

Given the extreme variability of the VARTIG modules, it does not seem surprising that their spread is currently limited to Thermococci. Conceivably, whatever the specific biological functions of VARTIG are, these are limited to interactions among Thermococci and perhaps their viruses, so that HGT to other organisms would not result in fixation of VARTIG in their genomes. Although Ig domain-containing proteins encoded in other currently available archaeal genomes, are not found in such highly variable loci, some of these might also contribute to cell-cell adhesion, especially those that are unlikely to be involved in inter-microbial conflicts or immunity because they consist entirely of domains frequently found in adhesins and S-layer proteins (Figure 3A).

## Conclusion

**FIGURE 4:**
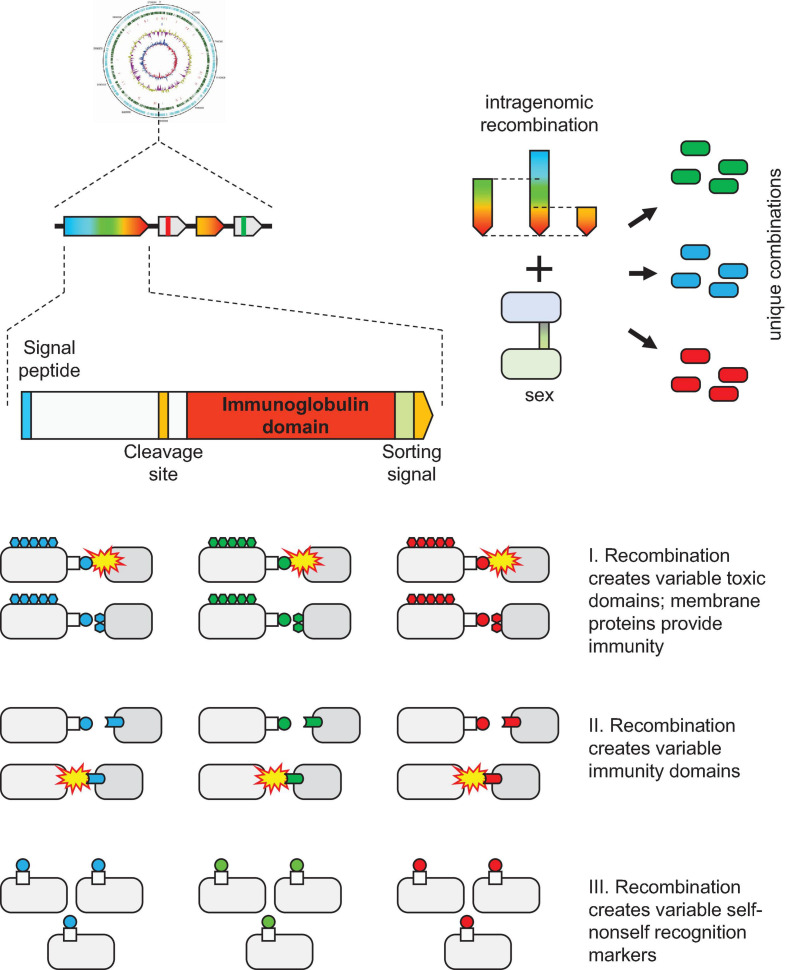
Three hypotheses on the function of VARTIG.

We describe here a gene module that is widespread among *Thermococcales* and is characterized by variability that is so far unprecedented in archaea. These loci contain an upstream gene coding for a large protein containing several Ig domains and unique combinations of downstream genes, some of which also encode Ig domains. In the large Ig domain-containing proteins (arCOG07710), the C-terminal Ig domain sequence is hypervariable, apparently, as a result of recombination between genes from different *Thermococcales*. To reflect this hypervariability, we named this gene module VARTIG, after VARiable *Thermococcales* IG. The overall organization of the VARTIG modules is similar to the organization of PTS. We searched all archaeal genomes for Ig-containing variable proteins and identified numerous such proteins, but no counterpart to VARTIG outside *Thermococcales* and no domains with a comparable level of variability. The specific functions of VARTIG remain unknown. However, the identified features of this system suggest three testable hypotheses: (i) involvement in inter-microbial conflicts analogous to PTS, (ii) role in innate immunity analogous to the vertebrate complement system, and (iii) function in self vs nonself recognition analogous to the vertebrate MHC (Figure 4). The latter two hypotheses appear to be of particular interest due to the unsuspected analogies to the vertebrate immune mechanisms.

## Data Availability Statement

The data used in the study are publicly available at the FTP site (https://ftp.ncbi.nlm.nih.gov/genomes/). Detailed information for specific accessions is provided in Supplementary Tables 1–3.

## Author Contributions

KM initiated the study. KM and EK wrote the manuscript. All authors analyzed the data, edited, and approved the manuscript.

## Conflict of Interest

The authors declare that the research was conducted in the absence of any commercial or financial relationships that could be construed as a potential conflict of interest.

## Publisher’s Note

All claims expressed in this article are solely those of the authors and do not necessarily represent those of their affiliated organizations, or those of the publisher, the editors and the reviewers. Any product that may be evaluated in this article, or claim that may be made by its manufacturer, is not guaranteed or endorsed by the publisher.

## Footnotes

1
https://ftp.ncbi.nlm.nih.gov/genomes/

2
http://pfam.xfam.org/clan/E-set

## Supplementary Material

Supplementary Figure 1 Supporting alignments for key components of thermococcal hypervariable system and for alpha 2-macroglobulin catalytic domain.

Supplementary Figure 2 Phylogenetic tree of the ∼200 last amino acids of Ig-like domain containing proteins associated with thermococcal hypervariable system.

Supplementary Figure 3 Mosaic proteins containing Ig-like domain. In each scheme is shown high similarity region for each blast hit which is mapped on the query protein (first line). The length of the line is proportional for respective protein length. For query sequence the domain organization is schematically shown above the respective line. The BLAST search was performed with the parameters modified as follows: “-seg no -comp_based_stats 0 -dbsize 20000000 -gapopen 32767 -gapextend 32767.” This modification allows to identify ungapped regions of a very high similarity, which might be suggestive of intraprotein recombination (or mosaicism).

Supplementary Table 1 Detailed information for the genes encoded in thermococcal variable system loci.

Supplementary Table 2 Two worksheets. (1) Phyletic patterns (protein presence/absence) for *Thermococcales* COGs associated with VARTIG system; (2) Phyletic patterns (protein presence/absence) for the arCOGs mentioned in the text.

Supplementary Table 3 Five worksheets. (1) Results of PSI-BLAST search for homologs of PAP_07455 protein. (2) Detailed information for the neighborhoods of the genes found using first strategy of the search for analogous systems in other archaea. (3) Detailed information for the genes found using second strategy of the search for analogous systems in other archaea. (4) Detailed information for the genes found using third strategy of the search for analogous systems in other archaea. (5) Detailed information for the neighborhoods of archaeal gene coding for alpha 2-macroglobulin homologs.
