# Supplementary material for: A Unique Gene Module in Thermococcales Archaea Centered on a Hypervariable Protein Containing Immunoglobulin Domains
Source: Front Microbiol. 2021 Aug 18;12:721392. doi: 10.3389/fmicb.2021.721392 (PMC8416519; doi:10.3389/fmicb.2021.721392)
Supplement: Supplementary Figure 1 — Supporting alignments for key components of thermococcal hypervariable system and for alpha 2-macroglobulin catalytic domain. [file Image_1.pdf]

Supplementary Figure S1. Evidence for sequence features of the protein families identified in this work.

A. **Thermoccocal CARDB domain containing proteins.** Alignments were colored using http://www.bioinformatics.org/sms2/color_align_cons.html server with default amino acid groups with 20% consensus. Residues corresponding to identified motifs (see details on the Figure 2 and the text) are within signal peptide region are colored red. Sequences that were truncated to alignable only region are denoted by asterisks next to the respective accession.

```
WP_058939188_1  -----------------------------------------------------------MRKAVG-----------VFLIILLLGSIVAAAGST---AYA 27
WP_014788798_1  ----------------------------------------------------------MVTAAGS-----------T--------------------AQA 11
WP_048151755_1  -----------------------------------------------------------MRRAVG-----------VLLVILLLGSMVTAAGST---AQA 27
WP_088181398_1  -----------------------------------------------------------MKRLGG-----------IWLIFLIVGMSVSVPSYS-----K 25
WP_048165390_1  -----------------------------------------------------------MRKILI-----------LVLSYLILSLSFITAQNT--TITV 28
WP_088865610_1  -----------------------------------------------------------MKSIGG-----------IWLISLIVGMSLSMAVSS-----K 25
WP_014012907_1  -----------------------------------------------------------MRKAVG-----------VLLITLLLGSIVAAAGST---VYA 27
WP_099209391_1  --------------------------------------------------------------MLG-----------VVFSSAAAGGIASGVPVS------ 21
WP_088863686_1  ---------------------------------------------------------------------------------------------------- 0
WP_068319935_1  ------------------------------------------------------------------------------MVILILMVSVSQAMEE----GF 18
WP_011013125_1  ----------------------------------------------------------MVRRSIV-----------FFVISLLLGGLVNPSIVQ------ 25
WP_042692137_1  -----------------------------------------------------------MKRGLG-----------VLIIGIMLGALVYGAMVS-TPMVL 29
WP_052696221_1  -----------------------------------------------------------MRKAGG-----------LVIVILLLGSLINI---------G 21
WP_014122995_1  ---------------------------------------------------------------------------------------------------- 0
WP_088886144_1  ---------------------------------------------------------------------------------------------------- 0
WP_088856136_1  ---------------------------------------------------------------------------------------------------- 0
WP_015857921_1  ---------------------------------------------------------------------------------------------------- 0
WP_014733996_1  -----------------------------------------------------------MKRLMG-----------VIILVLLVGSTLSVISAN----TF 26
WP_088886109_1  ---------------------------------------------------------------------------------------------------- 0
WP_088882563_1  -----------------------------------------------------------MERLVG-----------VWLIALLAGAVVGTPIFTPVVGAS 30
WP_015859709_1  ---------------------------------------------------------------------------------------------------- 0
WP_014122675_1  ---------------------------------------------------------------------------------------------------- 0
WP_014122677_1  ---------------------------------------------------------------------------------------------------- 0
WP_056934707_1  -----------------------------------------------------------MKRALS-----------IMLIALILGLGFSAAVSQ---SFV 27
WP_068320152_1  ---------------------------------------------------------------------------------------------------- 0
WP_082781392_1  ---------------------------------------------------------------------------------------------------- 0
WP_010884703_1  -----------------------------------------------------------MRKYVG-----------YLFVILITGSIIGQVTVQ---GSY 27
WP_062388971_1  -----------------------------------------------------------MSYVAA----------------------------------A 7
WP_074631387_1  ---------------------------------------------------------------------------------------------------- 0
WP_010884704_1  ---------------------------------------------------------------------------------------------------- 0
WP_056934708_1  ---------------------------------------------------------------------------------------------------- 0
WP_048151448_1  ---------------------------------------------------------------------------------------------------- 0
WP_088863691_1  ---------------------------------------------------------------------------------------------------- 0
WP_048810998_1  ---------------------------------------------------------------------------------------------------- 0
WP_048148049_1  ------------------------------------------------------------MQSMR-----------KLMVPLVLGLIFLS---------I 20
WP_055428947_1  ---------------------------------------------------------------------------------------------------- 0
WP_099209381_1  ---------------------------------------------------------------------------------------------------- 0
WP_014122994_1  ---------------------------------------------------------------------------------------------------- 0
WP_048165381_1  ---------------------------------------------------------------------------------------------------- 0
WP_048165382_1* ---------------------------------------------------------------------------------------------------- 0
WP_068319940_1  ---------------------------------------------------------------------------------------------------- 0
WP_068320169_1  ---------------------------------------------------------------------------------------------------- 0
WP_068320161_1  -----------------------------------------------------------MRSETS-----------LVLLGGIVGMYVAV---------- 20
WP_048055992_1  ---------------------------------------------------------------------------------------------------- 0
WP_074631389_1  ---------------------------------------------------------------------------------------------------- 0
WP_014012910_1  ---------------------------------------------------------------------------------------------------- 0
WP_015857844_1  ----------------------------------------------------------MNKQLVF-----------I----------------------- 8
WP_099209388_1  ---------------------------------------------------------------------------------------------------- 0
WP_088865600_1  ---------------------------------------------------------------------------------------------------- 0
WP_048165388_1  ---------------------------------------------------------------------------------------------------- 0
WP_048055998_1  ---------------------------------------------------------------------------------------------------- 0
WP_088863688_1  ---------------------------------------------------------------------------------------------------- 0
WP_048151737_1  ---------------------------------------------------------------------------------------------------- 0
WP_048055995_1  ---------------------------------------------------------------------------------------------------- 0
WP_055430151_1  ---------------------------------------------------------------------------------------------------- 0
WP_048150566_1  ---------------------------------------------------------------------------------------------------- 0
WP_099209386_1  ---------------------------------------------------------------------------------------------------- 0
WP_088886110_1  -----------------------------------------------------------MKRWGG-----------IWLIFLIVGMSLSTAFSS-----S 25
WP_068320165_1  ---------------------------------------------------------------------------------------------------- 0
WP_055428943_1  ---------------------------------------------------------------------------------------------------- 0
WP_014122673_1  ---------------------------------------------------------------------------------------------------- 0
WP_088856137_1  -----------------------------------------------------------MRRAVG-----------LWLILLVLSMGISTV--------R 22
WP_048152596_1  ---------------------------------------------------------------------------------------------------- 0
WP_048151739_1  ---------------------------------------------------------------------------------------------------- 0
WP_088865604_1  ---------------------------------------------------------------------------------------------------- 0
WP_088865603_1  ---------------------------------------------------------------------------------------------------- 0
WP_048150577_1  ---------------------------------------------------------------------------------------------------- 0
WP_055430149_1  ---------------------------------------------------------------------------------------------------- 0
WP_068320157_1  -----------------------------------------------------------MK--------------------------------------- 2
WP_014788172_1* ---------------------------------------------------------------------------------------------------- 0
WP_048165379_1  ---------------------------------------------------------------------------------------------------- 0
WP_068320171_1  ---------------------------------------------------------------------------------------------------- 0
WP_088865607_1* ---------------------------------------------------------------------------------------------------- 0
WP_058939190_1* ---------------------------------------------------------------------------------------------------- 0
WP_068319946_1  ---------------------------------------------------------------------------------------------------- 0
WP_048150353_1  ---------------------------------------------------------------------------------------------------- 0
```

```
WP_048150344_1* ----------------------------------------------------------NVHSQIA-----------IVLLGAIIVVHIVT---------- 21
WP_048055999_1  ---------------------------------------------------------------------------------------------------- 0

WP_058939188_1  TSVDAQNDPYEKFWEILNRESELVVRLNATGNT-----TLVNELIQNSRLGAENAANISALIWQALEELKTSGVKTFYTAEELREMAQNISQNGLPQETV 122
WP_014788798_1  TSLETRNDPYEKFWEILNREAELVVQFNATGNT-----TLARELIQNSRLGAENAANISALIWQALEELKASGVKTYYTAEELREMAQNISRNGLPQETV 106
WP_048151755_1  TSLETQNDPYEKFWEILNKEAELVVQFNATRNT-----TLAQELIQNSRLGAENAANISALIWQALEELKASGVKTYYTAEELREMARNISRNGLPQETV 122
WP_088181398_1  PVAAATSDPYEAFWQILNREAELVVQFNSTGNA-----SLAQELIQNSHLGAENAANISALIWQALEELKASGVKTYYTAEELREMAQNISQNGLPDETV 120
WP_048165390_1  NALENENQPYEEFWNILNKEAELIIKLNQTADQ-----NTARELITNSQKGAENAVQISALIWQNLKELEQSGVKTYYTTQELRSIAEEIKQNGLPAETV 123
WP_088865610_1  PVMAVSNDPYQAFWEILNREAELVVQFNETGNA-----SLAVELIQNSFLGAENAANISALIWQSLDELKTSGIKTYYTAQELREMAQNISQNGLPEETV 120
WP_014012907_1  TTVEAQDDPYEKFWEILNKEAELVVQFNATGNI-----VLARELIQNSRIGAENAANISALIWQALEELKASGVKTHYTAEELREIARNISRNGLPQETV 122
WP_099209391_1  -----ENDAYAQFWNVLYREAYLVGVVNESANAGHINATAARELIDNSRAGEENAANISAQIWLALGELKKAGVKLKYSAEELRKMAEDIKKNGLPPETV 116
WP_088863686_1  -----------------------MVEVE-KGNL-----TLVPELIQNSRLGADNAANISALIWQALEELKASGVKTYYTAEELREMAQNISGNGLPQETV 71
WP_068319935_1  VLASAAEDPYKEFWDILYREADLVVQLNKTGDG-----NIAKELINNSRRGAENAANISSSIWIALQELKKSGVKLYYTEDELRKMAEDIKRNGLPQETV 113
WP_011013125_1  ---ASTQDPYEEFWEILWREARLVGEAE-SGNT-----TAINELIENSKAGAENAIIISTQTWQTLQELKSAGVKLYYTEDELKEMIEEIKAKGLPQETV 116
WP_042692137_1  ANEVNGDKAYDDFWRILNDEAYLVVAFNESLYSGNPNATLAQKLIENSRQGELNSANISAQIWLALEELKKSGVQLYYSADELRKMAEEIKKNGLPAETV 129
WP_052696221_1  TVQALSQDPYEDFWRILEKEANLIPEAG-SGNV-----TAINELFQNSHAGAENAALISAQTWQALQELKSAGVKLYYTEEELRRMALEIKSQGLPQETV 115
WP_014122995_1  ----------------------------------------------------------------------------------MSHLNVEFIPPGL----- 13
WP_088886144_1  ---------------------------------------------------------------------------------------------------- 0
WP_088856136_1  ---------------------------------------------------------------------------------------------------- 0
WP_015857921_1  ---------------------------------------------------------------------------MDYSEDELKDMAREIAQNGLPDEVV 25
WP_014733996_1  VVAQATEDPYNMFWEILSKEAELVVEANRTGNI-----TIIKELIRNSQLGAENAANLSALIWISIEELKKTGVKLYYTEAELKEMANEIKENGLPEETK 121
WP_088886109_1  ---------------------------------------------------------------------------------------------------- 0
WP_088882563_1  GTSTGTTSAYTEFWDILNREAELVVGVE-IGNL-----GLAPDLVQNSRLGAENAANLSALTWQALEELKSSGVKTYYSAEELREMSLNISENGLPPETV 124
WP_015859709_1  ---------------------------------------------------------------------------------------------------- 0
WP_014122675_1  ---------------------------------------------------------------------------------------------------- 0
WP_014122677_1  ---------------------------------------------------------------------------------------------------- 0
WP_056934707_1  LAGTQDDKAYEDFWNILNREAELIVELNSTTDVNEIQ-SLALQLIQNSQNGSSNAAQISALIWQSLEELENSGVKTYYTAEELKQMAEEIRQNGLPQETV 126
WP_068320152_1  ---------------------------------------------------------------------------------------------------- 0
WP_082781392_1  ---------------------------------------------------------------------------------------------------- 0
WP_010884703_1  AMAMDDSNPYEEFWKILYREAELVVKVE-NGNI-----SAIQELIENSRKGEENAANISSLTWTSLKELESSGVKMYYTEEELRKMAEDIAENGLPEDTV 121
WP_062388971_1  TDSEDTTSVYNAFWDILNREAMLVLEAQ-EGNT-----TAAQELITNSREGEKNAAQISALVWEALEELDSSGVKLYYTEEELRGMAQEIKQKGLPSETV 101
WP_074631387_1  ---------------------------------------------------------------------------------------------------- 0
WP_010884704_1  ---------------------------------------------------------------------------------------------------- 0
WP_056934708_1  ---------------------------------------------------------------------------------------------------- 0
WP_048151448_1  ---------------------------------------------------------------------------------------------------- 0
WP_088863691_1  ---------------------------------------------------------------------------------------------------- 0
WP_048810998_1  ---------------------------------------------------------------------------------------------------- 0
WP_048148049_1  PAGAESTDPYAEFWEILERTAGLIVEFNATANE-----TLAPALINSTLDGIDNAITISTLVWEAKEELEASGVQLYYTEEELRAMAEEVSQNGLPPETV 115
WP_055428947_1  ----------------------------------------------------------------------------------MPDPPTSACLSGVPLE-- 16
WP_099209381_1  ---------------------------------------------------------------------------------------------------- 0
WP_014122994_1  ---------------------------------------------------------------------------MYFTVKVRNYRPDKLDVRGFIED-- 23
WP_048165381_1  ---------------------------------------------------------------------------------------------------- 0
WP_048165382_1* ---------------------------------------------------------------------------------------------------- 0
WP_068319940_1  ---------------------------------------------------------------------------------------------------- 0
WP_068320169_1  ---------------------------------------------------------------------------------------------------- 0
WP_068320161_1  ---------------------------------------------------AKSDVRLEGIIFNGL---------------------------------- 35
WP_048055992_1  ---------------------------------------------------------------------------------------------------- 0
WP_074631389_1  ---------------------------------------------------------------------------------------------------- 0
WP_014012910_1  ---------------------------------------------------------------------------------------------------- 0
WP_015857844_1  ---------------------------------------------------------------------------------------------------- 8
WP_099209388_1  ---------------------------------------------------------------------------------------------------- 0
WP_088865600_1  ---------------------------------------------------------------------------------------------------- 0
WP_048165388_1  ---------------------------------------------------------------------------------------------------- 0
WP_048055998_1  ---------------------------------------------------------------------------------------------------- 0
WP_088863688_1  ---------------------------------------------------------------------------------------------------- 0
WP_048151737_1  ---------------------------------------------------------------------------------------------------- 0
WP_048055995_1  ---------------------------------------------------------------------------------------------------- 0
WP_055430151_1  ---------------------------------------------------------------------------------------------------- 0
WP_048150566_1  ---------------------------------------------------------------------------------------------------- 0
WP_099209386_1  ---------------------------------------------------------------------------------------------------- 0
WP_088886110_1  QVIAASSDPYQEFWDILNREAELVVGIE-NGNL-----SLAPELIQNSRLGAENAANISALIWQALEELKASGVKTYYTAEELREMAQNISENGLPQETV 119
WP_068320165_1  ---------------------------------------------------------------------------------------------------- 0
WP_055428943_1  ---------------------------------------------------------------------------------------------------- 0
WP_014122673_1  ---------------------------------------------------------------------------------------------------- 0
WP_088856137_1  PVKALSNDPYEAFWEILDRESTLVVEVE-DGNL-----SAALELIQNSRLGAENAANISALTWQALEELKASGVKTYYTAEELREMAQNISQNGLPQETM 116
WP_048152596_1  ---------------------------------------------------------------------------------------------------- 0
WP_048151739_1  ---------------------------------------------------------------------------------------------------- 0
WP_088865604_1  ---------------------------------------------------------------------------------------------------- 0
WP_088865603_1  ---------------------------------------------------------------------------------------------------- 0
WP_048150577_1  ---------------------------------------------------------------------------------------------------- 0
WP_055430149_1  ---------------------------------------------------------------------------------------------------- 0
WP_068320157_1  ---------------------------------------------------------------------------------------------------- 2
WP_014788172_1* ---------------------------------------------------------------------------------------------------- 0
WP_048165379_1  ---------------------------------------------------------------------------------------------------- 0
WP_068320171_1  ---------------------------------------------------------------------------------------------------- 0
WP_088865607_1* ---------------------------------------------------------------------------------------------------- 0
WP_058939190_1* ---------------------------------------------------------------------------------------------------- 0
WP_068319946_1  ---------------------------------------------------------------------------------------------------- 0
WP_048150353_1  ---------------------------------------------------------------------------------------------------- 0
WP_048150344_1* ---------------------------------------------------TKLGIQWEAITLNGL---------------------------------- 36
WP_048055999_1  ---------------------------------------------------------------------------------------------------- 0

WP_058939188_1  EALKAQGWTDEQIQALEEYIVKNADEINEDFNMTAFLEDFSMAFIDVAFKYNEYGAWALKTGYWKNIEEAPSTAGLSLQ-----VNPILKRSWIETYASY 217
WP_014788798_1  EALKAQGWTDEQIQALEEYIVKNADGINEDFNMTAFLEEFSMAFIDVAFKYNEYETWTLEKWKWTNPQEINMNLGNLL------INPVLADEWLDLYLGH 200
WP_048151755_1  EALKAQGWSDEQIQALEEYIVRNADEINEDFNMTAFLEEFSMAFIDVAFKYNEYETWTLEKWKWTQPTGITGGDN-RTL-----IHPLLLRGWVEFYRSY 216
WP_088181398_1  EALKEQGWTDEQIQALKEYIVQNADEINEDFNMTAFLEDFSMAFINVAFKYNEYEAWTLRKWKWTNAQKTPQANDNYL------LNPALGGEWTTLYFRY 214
WP_048165390_1  STLKEQGWSDSEIQALQNYIVQNADQITEDFNMTAFLEEFSTAFINVAFKYNEYETWAIEKWKWTNAGEAPQENHGIA------INPILAEEWALFYMRY 217
WP_088865610_1  QALKSQGWTDEQIQALEDYIAQNADNINEDFNMTAFLEDFSLAFIDVAFKYNHYESWTLEKWKWSQPAEPPQATNNEM------VNPILAKEWVEFYRAY 214
WP_014012907_1  EALKTQGWTDEQIQALEDYIAKNAGEINEDFNMTAFLEEFSMAFIDVAFKYNEYETWTLKKWKWIHSAEFQQTRDNEI------INPTLAKGWLEFYKAY 216
WP_099209391_1  KELKSQGWTEEEIKALQEYIAKNADNITTGFDMGSFLTNFSRAFVMVGFKYASYESWALERWKWAHPKETREQALKAYGSYPKKLLPGISRDWVSFYTAY 216
WP_088863686_1  NALKEQGWTDEQIQALEEYIAQNSGNITEDFNMTAFIGDFSTAFVKVSFKYNHYEAWALEKWKWAQPTKTPATNNKNEL-----INPVLANQWVSFYREY 166
WP_068319935_1  KALKEQGWTDSEIKALEEYIAENADKIRGDFDMGEFLLNMSRAFIAAGFKYNYYETWALKRWLWTNPKELPELKPGTET-----MNPLLAEEWMTFM--- 205
WP_011013125_1  QALKNEGWSDEEIEEFEQYLIEHADEVKGDFNMSKFLTDFSNAFIRIAFKYNSYEARALELKYWKDYQSKPGDTLITRE-----IPPQIAREWISFYRAY 211
WP_042692137_1  NELKAQGWTDDEIKALEEYIAKNADNITSGFDMGRFLTNFSQAFVMVGFKYADYETWALEKWKWPNAAKSREDAMRIYGSAKRQIVPDLSDQWSEFYHAY 229
WP_052696221_1  RALKEQGWSDEEVNNLQEYLIQHADEVSGDFNMSEFLTEFSNAFLRVAFKCNEYESKALEWKYWSNPGTF--SIRGEEV-----LLPAISSEWGAFYEVY 208
```

```
WP_014122995_1   -----------NLSNFPDF-----DSIAGSFSMSAFLQALSDSFIFTGWKYAKYETWAVEKEYWP--TSPSQAPSGTLT-----ANPLVSTS--SIWSAI 88
WP_088886144_1   ---------------------------------------------------------------------------------------------------- 0
WP_088856136_1   ---------------------------------------------------------------------------------------------------- 0
WP_015857921_1   DELRSSGWNDEEIKALEEYIKENTDSITGSFSMSAFLQALSDSFIFTGWKYAKYETWAVEREFWP--ASPSQAPSGTLT-----SNPLVSTS--SIRSAI 116
WP_014733996_1   SVLREQGWNEEEIKSLEDYIVRHSDDIKGDFNMTEFLEDLSLAFVEVGYKYNKYEAWALKRARWTNSLTPPSLSGDTKM-----INPLFYDIWLQLYREY 216
WP_088886109_1   ---------------------------------------------------------------MDPPAEAPTGNKGEI------INPALSGDWVILYLRY 31
WP_088882563_1   NALKEQGWTDEQIRALEDYIVQNGENITEGFNMTSFLEDLSTSFIRVGFKYNHYESWALEMWKWTRPIEAPTTNSEDRL-----INPLMAVRWVSFYREY 219
WP_015859709_1   ---------------------------------------------------------------------------------------------------- 0
WP_014122675_1   ---------------------------------------------------------------------------------------------------- 0
WP_014122677_1   ---------------------------------------------------------------------------------------------------- 0
WP_056934707_1   QTLKEQGWSDDEIQALQEYIAENTDEITEDFNLTKFLKDFSQAFIQVAFKYNQYEIWAIEKWKWRNPVDRNTPQGEKM------INPLLADEWINFYKTY 220
WP_068320152_1   ---------------------------------------------------------------------------------------------------- 0
WP_082781392_1   -----------------------MPHIQGEPKTKEI---------------------------------------------------------------- 13
WP_010884703_1   RALKEQGWSDQEIKALEDYIAKNKDNITGDFNMSEFLMNFSRAFVKVGFKYAEYESWALEKWKWEGKANFTGSLPSNEG-----LNPFLINELSDVRLAY 216
WP_062388971_1   KTLREQGWSDGEIQALQNYIIQNADNITGDFNMSEFLENFSTAFVKVGFKYAHYETFGLEKWKWGSTENFTGNLPQDVS-----INPLLAEEWVNFYKAY 196
WP_074631387_1   ---------------------------------------------MITARYTGSKYWNYD---------------------------------------- 15
WP_010884704_1   ---------------------------------------------------------------------------------------------------- 0
WP_056934708_1   ---------------------------------------------------------------------------------------------------- 0
WP_048151448_1   ---------------------------------------------------------------------------------------------------- 0
WP_088863691_1   ---------------------------------------------------------------------------------------------------- 0
WP_048810998_1   ---------------------------------------------------------------------------------------------------- 0
WP_048148049_1   ENFREQGWSESEILALQSYIANNVDEINGDFNMSEFLENFSIAFINVSFKYADYSAWALERWKWNGEGTVPEARARG-------IPPGLLSEWNSFYSAY 208
WP_055428947_1   ---------------------------------------------------------------------------------------------------- 16
WP_099209381_1   ---------------------------------------------------------------------------------------------------- 0
WP_014122994_1   ----EDGAVIVKMGGSGVYTIPARGEKSFSFSYTLY---------------------------------------------------------------- 55
WP_048165381_1   ---------------------------------------------------------------------------------------------------- 0
WP_048165382_1*  ---------------------------------------------------------------------------------------------------- 0
WP_068319940_1   ---------------------------------------------------------------------------------------------------- 0
WP_068320169_1   ---------------------------------------------------------------------------------------------------- 0
WP_068320161_1   ------------------------------------FAVAACEFIKLQLQSS---------WRFCGIA-------------------------------- 58
WP_048055992_1   ---------------------------------------------------------------------------------------------------- 0
WP_074631389_1   ---------------------------------------------------------------------------------------------------- 0
WP_014012910_1   ---------------------------------------------------------------------------------------------------- 0
WP_015857844_1   ------------------------------FTLLSF---------------------------------------------------------------- 14
WP_099209388_1   ---------------------------------------------------------------------------------------------------- 0
WP_088865600_1   ---------------------------------------------------------------------------------------------------- 0
WP_048165388_1   ---------------------------------------------------------------------------------------------------- 0
WP_048055998_1   ---------------------------------------------------------------------------------------------------- 0
WP_088863688_1   ---------------------------------------------------------------------------------------------------- 0
WP_048151737_1   ------------------------------------------------------------------------------------MKPT------------ 4
WP_048055995_1   ---------------------------------------------------------------------------------------------------- 0
WP_055430151_1   ---------------------------------------------------------------------------------------------------- 0
WP_048150566_1   ---------------------------------------------------------------------------------------------------- 0
WP_099209386_1   ---------------------------------------------------------------------------------------------------- 0
WP_088886110_1   DALKAQGWTDAQIRALEEYIVQNGANITEDFNMTAFLEEFSMAFISVAFKYNHYEAWALEKWKWTHPLKLQLGTKGR----------------------- 196
WP_068320165_1   ---------------------------------------------------------------------------------------------------- 0
WP_055428943_1   ---------------------------------------------------------------------------------------------------- 0
WP_014122673_1   ---------------------------------------------------------------------------------------------------- 0
WP_088856137_1   EALKAQGWTDEQIQALEEYIVQNADEIDEDFNMTAFLEKFSMAFIDVAFKYNQYEAWALEK--------------------------------------- 177
WP_048152596_1   ---------------------------------------------------------------------------------------------------- 0
WP_048151739_1   ---------------------------------------------------------------------------------------------------- 0
WP_088865604_1   ---------------------------------------------------------------------------------------------------- 0
WP_088865603_1   ---------------------------------------------------------------------------------------------------- 0
WP_048150577_1   ---------------------------------------------------------------------------------------------------- 0
WP_055430149_1   ---------------------------------------------------------------------------------------------------- 0
WP_068320157_1   ------------------------------------------------------------------------------------VRPV------------ 6
WP_014788172_1*  ---------------------------------------------------------------------------------------------------- 0
WP_048165379_1   ---------------------------------------------------------------------------------------------------- 0
WP_068320171_1   ---------------------------------------------------------------------------------------------------- 0
WP_088865607_1*  ---------------------------------------------------------------------------------------------------- 0
WP_058939190_1*  ---------------------------------------------------------------------------------------------------- 0
WP_068319946_1   ---------------------------------------------------------------------------------------------------- 0
WP_048150353_1   ---------------------------------------------------------------------------------------------------- 0
WP_048150344_1*  ------------------------------------FAVTTCIEFHSFSGES------------CSLA-------------------------------- 56
WP_048055999_1   ---------------------------------------------------------------------------------------------------- 0

WP_058939188_1   YNYSGDSMDIETFYSNVRGLNKLINALILDRIKPKEKEYEYTKFMPPHPIPRPPRNITLNGSISKYLHPPSKETEVTKVEKRAPNGGLVFTIERTKIVKV 317
WP_014788798_1   SEGN---------YSKMKSAAEKLRDKMYDMIVGKTLNKETLTELKEH--GDLWQLIELRRDV-------------YVMWW-LKNGGVVFRVYNSADNSS 275
WP_048151755_1   VEGD---------YKRMRDSIIPLRESMYELITYSSNSPKQVELAFFN----GESITITTGNL-------------RGVLW-GHDGSMAFRIEQEHFDRD 289
WP_088181398_1   LHSD---------YQRMEPAAKTLRQSMYNLLTGVGGNVNLLFYERGT------SPSKFQGSV-------------GSIVW-VEDGGLLFTVTTRRATAT 285
WP_048165390_1   SQKD---------YKNMSVGIKSLKDLTYKIITGSSGEQVTSMLYRTE------SILSKQGTL-------------ESIRW-IKNGGLVITFKSTQITPT 288
WP_088865610_1   LSGD---------YNNMKSKVYRLKGSMYKLLSNEKRNCTLTFLRDGN-------LVTQRGII-------------KGINH-TTNGSLVFTTLVTSSKGK 284
WP_014012907_1   SSRD---------YKGMEKKARQLRDSMYELLTGEKISGNLTFLKDEK-------VVTQTGMI-------------KSVNH-TTNGSLVFTVLVTIPDGD 286
WP_099209391_1   LKDN---------VSGMNSSLKNIIRYIHSIIMPSLYSSGTTF---------------------------------------VSGGGVVVQNVHRVGDSL 268
WP_088863686_1   SQGN---------YQKMEPAIKSLRDQTYDLLKGDWINIHKTPIVIYN----NSRLIQLTGRI-------------TSVQH-IENGGLIFTVTTTYATRD 239
WP_068319935_1   SSKT---------IEEKLLAIEKLQNKIIEIVSKGNVDY-------------------------------------------IENGGIVFEYRSTKERDQ 253
WP_011013125_1   LFEG---------IDEKLSTIERLKAKEVEVIKSNPKGRDGHVIIR------------------------------------NSNFSIVFNKFKRIPIIK 266
WP_042692137_1   LSGD---------VGGMSSALDSLAKGMDSILIHSKWTA-------------------------------------------VRDGGLIYLSGDVKSG-- 275
WP_052696221_1   RSKS---------IDDKLSALRRLKERELKLIESGGKLGDGFLVMKITTSPRKLTTLDETSTK-------------TSSVSSLPIRRVPIKPVPVKPKPC 286
WP_014122995_1   LNQN---------PGSFVYAVKATKNDVLDAISSAS-------------------------------------------AY-YSNGGIAFSTVTINSNGK 135
WP_088886144_1   --------------MGMEDSIEALRDSAYNFLTYQEKFVSLTFLRNGS-------VITKTGTL-------------GRVEW-TENGSMVFTTLVTSPSED 65
WP_088856136_1   ----------------MEVSIQNLRNEVYKLLLGQSGPITLMLLDD-------SGIISKRGQV-------------KNITW-VQNGGLIFTTISLTIGST 63
WP_015857921_1   INQN---------PNSLVYAVRSTKSDVVDTIYSAS-------------------------------------------IY-YSNGGIGFSMVTIDSRGK 163
WP_014733996_1   NSQN---------FDNAETLIKKLKRATYLLLFEKRVETPSGEY----------VLISPTYKL-------------ISLNW-TKDEGVVFTVIKIGSG-- 281
WP_088886109_1   SQKD---------YQGMDPAAETLKESMYRLIMGTGGSVNLLSYERTT------SLSQLPGSV-------------DSVKW-SKNGGLLFTVTARRMTTS 102
WP_088882563_1   SQQD---------YENMETAIKSLRDYVYLLMLGNSSGENRMLLYSPG----TDRLTSLTGKV-------------TNVEQ-TENGGLMFTVTLTRITGT 292
WP_015859709_1   ---------------------------------------------------------------------------------------------------- 0
WP_014122675_1   ---------------------------------------------------------------------------------------------------- 0
WP_014122677_1   -------------------------------------MIDRVKFND--------GSESKVLSV-------------GTIAY-TGPGSVESPEFIAPVGNS 41
WP_056934707_1   YTGT---------LEEQLSSLNKLQSRTYSLITGSLDRVNSALIPSAT--NSLQILPLNPLLV-------------KNITY-LNDNELTFETWEFIGQKT 295
WP_068320152_1   ---------------------------------------------------------------------------------------------------- 0
WP_082781392_1   ---------------------------------------------------------------------------------------------------- 13
WP_010884703_1   STNN---------MKGLLKAINELSSKMYEIFKYEMVEYSSLPSRFGQ--------LKNRGYV-------------TF----YKGGFVVITTRNIRYFPN 282
WP_062388971_1   LERN---------STELEESIEVLSSRTYALLTSHSSFKARAELLRNG------SLILRHETV-------------TGVDF-YSNGFAVIKTSSDYDDWR 267
WP_074631387_1   ---------------------------------------------------------------------------------------------------- 15
```

```
WP_010884704_1  ---------------------------------------------------------------------------------------------------- 0
WP_056934708_1  ---------------------------------------------------------------------------------------------------- 0
WP_048151448_1  ---------------------------------------------------------------------------------------------------- 0
WP_088863691_1  ---------------------------------------------------------------------------------------------------- 0
WP_048810998_1  ---------------------------------------------------------------------------------------------------- 0
WP_048148049_1  LEGD---------YEGALEGARTLRKRIYALSTAEDAER-------------------------------------------LGSGVLVMGLRWEHIPLN 256
WP_055428947_1  ---------------------------------------------------------------------------------------------------- 16
WP_099209381_1  ---------------------------------------------------------------------------------------------------- 0
WP_014122994_1  ---------------------------------------------------------------------------------------------------- 55
WP_048165381_1  ---------------------------------------------------------------------------------------------------- 0
WP_048165382_1* ---------------------------------------------------------------------------------------------------- 0
WP_068319940_1  ---------------------------------------------------------------------------------------------------- 0
WP_068320169_1  ---------------------------------------------------------------------------------------------------- 0
WP_068320161_1  ---------------------------------------------------------------------------------------------------- 58
WP_048055992_1  ---------------------------------------------------------------------------------------------------- 0
WP_074631389_1  ---------------------------------------------------------------------------------------------------- 0
WP_014012910_1  ---------------------------------------------------------------------------------------------------- 0
WP_015857844_1  ---------------------------------------------------------------------------------------LILSTPAVKA--- 24
WP_099209388_1  ---------------------------------------------------------------------------------------------------- 0
WP_088865600_1  ---------------------------------------------------------------------------------------------------- 0
WP_048165388_1  ---------------------------------------------------------------------------------------------------- 0
WP_048055998_1  ---------------------------------------------------------------------------------------------------- 0
WP_088863688_1  ---------------------------------------------------------------------------------------------------- 0
WP_048151737_1  ---------------------------------------------------------------------------------------------------- 4
WP_048055995_1  ---------------------------------------------------------------------------------------------------- 0
WP_055430151_1  ---------------------------------------------------------------------------------------------------- 0
WP_048150566_1  ---------------------------------------------------------------------------------------------------- 0
WP_099209386_1  ---------------------------------------------------------------------------------------------------- 0
WP_088886110_1  ---------------------------------------------------------------------------------------------------- 196
WP_068320165_1  ---------------------------------------------------------------------------------------------------- 0
WP_055428943_1  ---------------------------------------------------------------------------------------------------- 0
WP_014122673_1  ---------------------------------------------------------------------------------------------------- 0
WP_088856137_1  ---------------------------------------------------------------------------------------------------- 177
WP_048152596_1  ---------------------------------------------------------------------------------------------------- 0
WP_048151739_1  ---------------------------------------------------------------------------------------------------- 0
WP_088865604_1  ---------------------------------------------------------------------------------------------------- 0
WP_088865603_1  ---------------------------------------------------------------------------------------------------- 0
WP_048150577_1  ---------------------------------------------------------------------------------------------------- 0
WP_055430149_1  ---------------------------------------------------------------------------------------------------- 0
WP_068320157_1  ---------------------------------------------------------------------------------------------------- 6
WP_014788172_1* ---------------------------------------------------------------------------------------------------- 0
WP_048165379_1  ---------------------------------------------------------------------------------------------------- 0
WP_068320171_1  ---------------------------------------------------------------------------------------------------- 0
WP_088865607_1* ---------------------------------------------------------------------------------------------------- 0
WP_058939190_1* ---------------------------------------------------------------------------------------------------- 0
WP_068319946_1  ---------------------------------------------------------------------------------------------------- 0
WP_048150353_1  ---------------------------------------------------------------------------------------------------- 0
WP_048150344_1* ---------------------------------------------------------------------------------------------------- 56
WP_048055999_1  ---------------------------------------------------------------------------------------------------- 0

WP_058939188_1  YSDFLIKDIGRSGDADVKSTPEVWCPETWITINRTVYYWPSALEAYELSSNIMALLEAKKRGNNNPEIDKIINQKMAELKQSLGVYMI----YSTSEIKR 413
WP_014788798_1  YG---------------------------------VYYWPNATAAYELASNIEALLTAKELGNNNPEVQWRLNDKIERLKSSLGVYPVKSGNSSDIVTVP 342
WP_048151755_1  GF----------------------------EINLTTYYWPGALEAYKLTSDVLVLVNAMKQGNNNPKLPKMLNQKVAELKDALEVTVV----SREVITNP 357
WP_088181398_1  EV----------------------------ITETKTYYWPNALRAYKLVNNIYALIRARNRGNNNTMIDSILNQKIAELKEALKVEIV----SSKVTRTP 353
WP_048165390_1  EI----------------------------ITERTTYYWPNALKAYELMGNVYALIKTRNYGNNNPKVESMLNQKVAELKEALKVLIISSEIKRSPIKSP 360
WP_088865610_1  YI----------------------------IWNVTTYYWPDALYAYELSSNILALVTAIESGNENDELVSILNQKVAELKDTMKVLII----ENKVLKNP 352
WP_014012907_1  YL----------------------------IWNATTYYWHNALNAYELLDNVLTLATAIASGNENWETESILHQKVAELKDSLKVVIV----KSESYRTR 354
WP_099209391_1  FG--------------------------------YSCYWPSALKAYNLTRQVYVLVETMSMGNRNPELKGLLNQKVAELKDALVVYYH---DETSINPNP 333
WP_088863686_1  RY--------------------------VIMENKTTYYWPKALKAYELIGNVYALVKAKNYGNDNTEIDRMLNQKVAELKDALTIVVI----YSHVSPIK 309
WP_068319935_1  SI-----RQDSIGTITLPKPKIPSPIIPSPTPTTNKYYWPTALEAYRTAAEIRTLLLGIKYGNGDRKLEEMLRNRISDLRSELKVYKV----FTSPSLIS 344
WP_011013125_1  CT-----------------------------------YWWNAIKAYNLTLQVESILKAQKLGNSNPELNYLLNHKVVELARALTAECY--GDGTTPIPKP 329
WP_042692137_1  -----------------------------------SYYWPTAVEAYRQLRRVYVLVNAMKLGNNNLDVKAMLNREVAELKDALVV--------YSKTISEW 333
WP_052696221_1  TI---------------------------------IYYW-NALKAYNLTLQVESILTAQKFGNSNPELNYRLNEKVSELADALRARRV----VNGGDCSG 348
WP_014122995_1  TV--------------------------------RRYSWENALNAYRTLGELYTIAVAIEKGNSNPELWGLAREKYDELEKYMKVRLV----STSFTPNP 199
WP_088886144_1  GF----------------------------MLNTTTYYWPSALRAYELTSNIMTLVKARNCGNNNSEVQWMLNQKVAELRSALKVIIV----STSTSTVQ 133
WP_088856136_1  KV----------------------------KTTRTIYYWPNATDAYRLIEDINTLINARSRGNTNPEIDWILNQKVAELKEALNVIII----NTTTWEIP 131
WP_015857921_1  TV--------------------------------RRYSWENALKAYRTLGELYTIAVAIEKGNSNPELWGLARQKYDELEEYMKVRLV----STSFTPNP 227
WP_014733996_1  ------------------------------VGIRETYYWPNALKAYELMGNVYTLVKSINLGNTNPTLRRMLNQKMAELGDALNVYVI----YREIIKRP 347
WP_088886109_1  EV----------------------------ITEKKTYYWPNALRAYELISNVIALVKARNYGNNNAKIDSILNQKIAELKDALKVIEV----SSEVTKTP 170
WP_088882563_1  EE----------------------------VVNTTTYYWPDALKAYELIGNVYTLVKARNYGNGNAEIDRMLNQKVAELKNALKVVRV----SSEVTTRP 360
WP_015859709_1  ---------------------------------------------------------------------------------------------------- 0
WP_014122675_1  ---------------------------------------------------------------------------------------------------- 0
WP_014122677_1  -----------------------------------------------LQASVSGIVKIRYRSVTSPG--------------------------------- 61
WP_056934707_1  EN-------------------KEICTYIYNKYNVTRYYWKSPLKAYKLIREIHSLLLAKQFGNNDVELEWQLNEKIGELKDAPTVING----ESTITVVP 372
WP_068320152_1  ---------------------------------------------------------------------------------------------------- 0
WP_082781392_1  ---------------------------------------------------------------------------------------------------- 13
WP_010884703_1  YT-----------------------------EEVTEYYWPTALKAYNLTRQIYVIVKAISMGNGDQELRKMLKDKMEELREALVVYIN------------ 341
WP_062388971_1  NL--------------------------KKVTVTDTYYWPAALRTYNLTRQVYVIVMAMNQGNDNPELWWILNQKVDELKEALKVYV-----SESVTEKP 336
WP_074631387_1  ---------------------------------------------------------------------------------------------------- 15
WP_010884704_1  ---------------------------------------------------------------------------------------------------- 0
WP_056934708_1  ---------------------------------------------------------------------------------------------------- 0
WP_048151448_1  ---------------------------------------------------------------------------------------------------- 0
WP_088863691_1  ---------------------------------------------------------------------------------------------------- 0
WP_048810998_1  ---------------------------------------------------------------------------------------------------- 0
WP_048148049_1  GT----------------------------FVTIERRYYWNAIESYRLALEAEAVLAAKVGGNGSEKLNGMLAGKVEELRDSL----------------- 311
WP_055428947_1  ---------------------------------------------------------------------------------------------------- 16
WP_099209381_1  ---------------------------------------------------------------------------------------------------- 0
WP_014122994_1  ---------------------------------------------------------------------------------------------------- 55
WP_048165381_1  ---------------------------------------------------------------------------------------------------- 0
WP_048165382_1* ---------------------------------------------------------------------------------------------------- 0
WP_068319940_1  ---------------------------------------------------------------------------------------------------- 0
WP_068320169_1  ---------------------------------------------------------------------------------------------------- 0
WP_068320161_1  ---------------------------------------------------------------------------------------------------- 58
WP_048055992_1  ---------------------------------------------------------------------------------------------------- 0
WP_074631389_1  ---------------------------------------------------------------------------------------------------- 0
```

```
WP_014012910_1  ---------------------------------------------------------------------------------------------------- 0
WP_015857844_1  ---------------------------------------------------------------------------------------------------- 24
WP_099209388_1  ---------------------------------------------------------------------------------------------------- 0
WP_088865600_1  ---------------------------------------------------------------------------------------------------- 0
WP_048165388_1  ---------------------------------------------------------------------------------------------------- 0
WP_048055998_1  ---------------------------------------------------------------------------------------------------- 0
WP_088863688_1  ---------------------------------------------------------------------------------------------------- 0
WP_048151737_1  ---------------------------------------------------------------------------------------------------- 4
WP_048055995_1  ---------------------------------------------------------------------------------------------------- 0
WP_055430151_1  ---------------------------------------------------------------------------------------------------- 0
WP_048150566_1  ---------------------------------------------------------------------------------------------------- 0
WP_099209386_1  ---------------------------------------------------------------------------------------------------- 0
WP_088886110_1  ---------------------------------------------------------------------------------------------------- 196
WP_068320165_1  ---------------------------------------------------------------------------------------------------- 0
WP_055428943_1  ---------------------------------------------------------------------------------------------------- 0
WP_014122673_1  ---------------------------------------------------------------------------------------------------- 0
WP_088856137_1  ---------------------------------------------------------------------------------------------------- 177
WP_048152596_1  ---------------------------------------------------------------------------------------------------- 0
WP_048151739_1  ---------------------------------------------------------------------------------------------------- 0
WP_088865604_1  ---------------------------------------------------------------------------------------------------- 0
WP_088865603_1  ---------------------------------------------------------------------------------------------------- 0
WP_048150577_1  ---------------------------------------------------------------------------------------------------- 0
WP_055430149_1  ---------------------------------------------------------------------------------------------------- 0
WP_068320157_1  ---------------------------------------------------------------------------------------------------- 6
WP_014788172_1* ---------------------------------------------------------------------------------------------------- 0
WP_048165379_1  ---------------------------------------------------------------------------------------------------- 0
WP_068320171_1  ---------------------------------------------------------------------------------------------------- 0
WP_088865607_1* ---------------------------------------------------------------------------------------------------- 0
WP_058939190_1* ---------------------------------------------------------------------------------------------------- 0
WP_068319946_1  ---------------------------------------------------------------------------------------------------- 0
WP_048150353_1  ---------------------------------------------------------------------------------------------------- 0
WP_048150344_1* ---------------------------------------------------------------------------------------------------- 56
WP_048055999_1  ---------------------------------------------------------------------------------------------------- 0

WP_058939188_1  VSCYDNIKPGQPRPM---------------NGADIEETVPVGNTSNDFQEPLNLPEWLI-KEALDMENTPGVLSINGIHVVVDRNDASE-----IKYHVE 492
WP_014788798_1  QDSIENITLPIINPGEKYGGITDPVKKPAMDSSNILNSQSIEITPSESNSETFKEGSRI-LTALNP-DTTRSLKINNVMVIPVEVSEDY-----ATYKVS 435
WP_048151755_1  DYKKLPPSD-PITPI-----DPINPEPLKNSIKGVDGGTLTPTESSSNQIPVIPADEIM-KIASDPGSLEGILKVDDIEVKPLTSGPNY-----VNYRVI 445
WP_088181398_1  IKNPKPITPIPIKPGDPGIPDPRPKPPLSSTLGTGMTSTVSDEDTGITVDGVPIDQDVL-KEALDPQSSPGTLMITDIAVVVDKNVPGE-----VKYHVK 447
WP_048165390_1  IPIKPGDPRIPA------------PIPVSPKGYSVDGTQTFEENLGVEVNGVLIEPEII-REALDTQGNIGQLKITEVKVIVDKNQPGK-----VEYHVK 442
WP_088865610_1  IRDDPIRPIEPIYPI--------------NPKLPAQPVSSLHTSGDGSVETNHIPTEIL-NEALNTEDNTGQLKITEVKVIVDKNLPGK-----VEYHVK 432
WP_014012907_1  KKDEPIKPTEPIYPI-------NPKLPVQPVYAGSTEEPTISTEPFTSIVTITPADEIT-TIALNPEDSQRILEINHITITPVEVSTDY-----ATYKVS 441
WP_099209391_1  NPNPPHPCVGGK------------------CLWTGTSSKGSGSLKAPSGSFNDRKDLLK-KFALDVDSNYGYIVIEGVDVIPVEVTGTY-----ATYRVV 409
WP_088863686_1  GPIKPEPIPIKPEPI--------KPGDPVMLPLDSATSGESYDSSPDSSLTTKVPDEIE-ALALNPEDSWGRFVIDGVSVEAVDKGTDY-----ARYRVV 395
WP_068319935_1  PFNVEKEGKLSV----------------------------------------------------------------EVKAIADEITTSY-----VRYHLK 375
WP_011013125_1  IITIPKPPKDTDLP---------------------------------------LNDQIA-REALMVGESEGILVIKAVRVNPVEVTSEY-----ATYQVE 384
WP_042692137_1  KFRPPENPPIIIDPC------EKNPLKCRTITSGSSGNSDYPLVGSISNLQTSRLTSDY-DIALDVNSNYGYISIKNVSVVVDDVTSTK-----ATYHIE 421
WP_052696221_1  VSPPEPGILGGS--------------------------------------------------LTEEALTAGRLEVKSVRVEPVEVAEDY-----ATYRVV 393
WP_014122995_1  VPPKPSPMPIPLDIS--------TPSLP---KTSS------------------------------TTTQTPVFKINAIRVVRTTRDSNP-----IKYHVL 253
WP_088886144_1  VPNEPIYPIEPIDPI-----YPIDPKLPVLPVSTTSPEESTTAELSESTTVITPADETA-LLALDPNNEEGRLVVDSVNVEAVEVTSDY-----VTYKVA 222
WP_088856136_1  TESPEP------SPI--------NPKPPITILSTEISGGS-KMNLENLEATTSQDDWIRMLAIKTGEENEGTLEIKDIHVEPINITSDY-----VTYRVV 211
WP_015857921_1  SPPRPRPMPTPLHLS--------TPE-----PLTS------------------------------TSSQEPVFRINAVRVVRASHDANP-----IKYHVA 279
WP_014733996_1  IYYISISSPIR--------------------------NNIGSVPLDRRAQRTSSEPDEI-EKVASGEYSIGKLVIKDVQVLTKKIDNGR-----ISYNVK 415
WP_088886109_1  ILLLKSTSGTAT-------------------------TSSITGGGEVIVSGVPVDESVL-VEALDTQNDSGTLVITDVSVVVDKNVPSS-----VEYHVE 239
WP_088882563_1  IKDGSSSDSGSTK----------------------------------------IADEIE-RLALDPQSSWGIFRVDDVEVEVTHKGTAYGMYKFAMYRVV 419
WP_015859709_1  ---------------------------------------------------------------------------------------------------- 0
WP_014122675_1  ---------------------------------------------------------------------------------------------------- 0
WP_014122677_1  GDTPNS------KP------------------------GD-PVKTASVQAYTPADYSTPTAPVLVMESYHGTIDL--------------------TSKI- 109
WP_056934707_1  VFKSCTSPI----------------------------------------------------IKLPEPPTLPVLTSTSTPVTSIEDTTQQ----------- 409
WP_068320152_1  ---------------------------------------------------------------------------------------------------- 0
WP_082781392_1  ---------------------------------------------------------------------------------------------------- 13
WP_010884703_1  --------------------------------------------------KYERKPWRD-LILLRSSPVTRGRLIISKEVVIDEATPDE-----VKYRIK 385
WP_062388971_1  LKIKPSLPPISIEP------------PKQPIGTLSSSGGSLSTQSTSEAPTTLKPTEVE-RKTLDPYTQEGILNA-GVSVIPVEVTDSF-----IQYQVV 417
WP_074631387_1  ---------------------------------------------------------------------------------------------------- 15
WP_010884704_1  ---------------------------------------------------------------------------------------------------- 0
WP_056934708_1  ---------------------------------------------------------------------------------------------------- 0
WP_048151448_1  ---------------------------------------------------------------------------------------------------- 0
WP_088863691_1  ---------------------------------------------------------------------------------------------------- 0
WP_048810998_1  ---------------------------------------------------------------------------------------------------- 0
WP_048148049_1  ---------------------------------------------------------------------------------------------------- 311
WP_055428947_1  ---------------------------------------------------------------------------------------------------- 16
WP_099209381_1  ---------------------------------------------------------------------------------------------------- 0
WP_014122994_1  ---------------------------------------------------------------------------------------------------- 55
WP_048165381_1  ---------------------------------------------------------------------------------------------------- 0
WP_048165382_1* ---------------------------------------------------------------------------------------------------- 0
WP_068319940_1  ---------------------------------------------------------------------------------------------------- 0
WP_068320169_1  ---------------------------------------------------------------------------------------------------- 0
WP_068320161_1  ---------------------------------------------------------------------------------------------------- 58
WP_048055992_1  ---------------------------------------------------------------------------------------------------- 0
WP_074631389_1  ---------------------------------------------------------------------------------------------------- 0
WP_014012910_1  ---------------------------------------------------------------------------------------------------- 0
WP_015857844_1  ---------------------------------------------------------------------------------------------------- 24
WP_099209388_1  ---------------------------------------------------------------------------------------------------- 0
WP_088865600_1  ---------------------------------------------------------------------------------------------------- 0
WP_048165388_1  ---------------------------------------------------------------------------------------------------- 0
WP_048055998_1  ---------------------------------------------------------------------------------------------------- 0
WP_088863688_1  ---------------------------------------------------------------------------------------------------- 0
WP_048151737_1  ---------------------------------------------------------------------------------------------------- 4
WP_048055995_1  ---------------------------------------------------------------------------------------------------- 0
WP_055430151_1  ---------------------------------------------------------------------------------------------------- 0
WP_048150566_1  ---------------------------------------------------------------------------------------------------- 0
WP_099209386_1  ---------------------------------------------------------------------------------------------------- 0
WP_088886110_1  ---------------------------------------------------------------------------------------------------- 196
WP_068320165_1  ---------------------------------------------------------------------------------------------------- 0
WP_055428943_1  ---------------------------------------------------------------------------------------------------- 0
WP_014122673_1  ---------------------------------------------------------------------------------------------------- 0
```

```
WP_088856137_1  ---------------------------------------------------------------------------------------------------- 177
WP_048152596_1  ---------------------------------------------------------------------------------------------------- 0
WP_048151739_1  ---------------------------------------------------------------------------------------------------- 0
WP_088865604_1  ---------------------------------------------------------------------------------------------------- 0
WP_088865603_1  ---------------------------------------------------------------------------------------------------- 0
WP_048150577_1  ---------------------------------------------------------------------------------------------------- 0
WP_055430149_1  ---------------------------------------------------------------------------------------------------- 0
WP_068320157_1  ---------------------------------------------------------------------------------------------------- 6
WP_014788172_1* ---------------------------------------------------------------------------------------------------- 0
WP_048165379_1  ---------------------------------------------------------------------------------------------------- 0
WP_068320171_1  ---------------------------------------------------------------------------------------------------- 0
WP_088865607_1* ---------------------------------------------------------------------------------------------------- 0
WP_058939190_1* ---------------------------------------------------------------------------------------------------- 0
WP_068319946_1  ---------------------------------------------------------------------------------------------------- 0
WP_048150353_1  ---------------------------------------------------------------------------------------------------- 0
WP_048150344_1* ---------------------------------------------------------------------------------------------------- 56
WP_048055999_1  ---------------------------------------------------------------------------------------------------- 0

WP_058939188_1  ISMVAKYNSVSKIRVNVSDKTS-----GGTDTGTIS-YLGPDEE-ETWRSGKFSARVSG----SSIRITGNVKITYRPVC---SSP-----------PTS 567
WP_014788798_1  MDITVSGASATNVTVEIEG--T-----ELKYSKDIG-AIDNGET-ITVESTVSGRVYGD----YEVEVSGKIKVTYQFIT---EYH-----------TNS 508
WP_048151755_1  VHMHTENNAVKNVKINLHDYST-----GRSDSETIS-QIDPYID-YNWESKTFSTSIRNEN--GKLTATGEIEITYTPEC---DKS-----------PLG 522
WP_088181398_1  VSFKAENNAVNNIRVSVKDYTT-----GDSDSGTVP-FLNSGES-HTWNSKEFT-YTHSSG--GELTVSGRVEITYTPSC---NDV-----------PLS 523
WP_048165390_1  ISFKAENNAVDTIRINVKDYTT-----GDSDTGTLS-FLNSGQS-YAWNSKRFT-LTHSMD--GELTVSGKVEITYTPSC---GAV-----------PLS 518
WP_088865610_1  VLFKAENNAVNNIRIDVEDRTT-----GDSDSGTLS-FLNSGES-YIWNSKKFT-LTHNME--GELIVSGKVEITYTPSC---GDV-----------PLS 508
WP_014012907_1  MDITASKASAVNVKVKIEG--T-----ELEYSKNIG-AINNGET-ITVESTISGRVYGD----YEVEVSGKIKVTYQFIT---EYR-----------TNS 514
WP_099209391_1  VHFRAVNNVVNDVKVKFHG--S-----GLSYQVTVG-YVHPDDGVKTVSSIVSSRIYGNGA--STVSVAGTVTVTGKSVP---TPR-----------PTE 485
WP_088863686_1  VRYHVEDNAVSNVKVTFDG--T-----ELTDSTSLG-FLSPGDS-DSVESEVSGYVRGS----GEVHVSGTVKVTYTSSS---GPV-----------PTS 468
WP_068319935_1  IILRAKDNDVYVSKIKVNG--T-----GLSFEKDVD-LLVHNNEDEIIETEISKEVYGEIS--NTVKVKGKVEITYSPIY---GST-----------P-- 449
WP_011013125_1  VEIEAQNNAVYDINVEFDG--T-----SLEDTEKVG-MLNKDEV-KVIKSTVSGKVYGS----GSVTVAGTVKITYKSYR---GNA-----------PMA 457
WP_042692137_1  VSFRAEHNAVSDVKINLTD--S-----SGSDSVKYS-MVHPDDGTRLWKSKKFTVNFNGK---TQVTVSGTVKISYLSTP---TPL-----------PMS 496
WP_052696221_1  VEVQAVGNAVSSVNVEFKG--V-----ELSDAESGG-FLYENEV-KTVASGVSGRVYGS----GSVEVSGVVRVSYESHS---GPV-----------PLS 466
WP_014122995_1  IDYTVTGGQVVVYNVTFNT--G-----SEEQTITPG-ILDPGSG--TIESGNFTAPVGVLDGSLKVKVGGIVTVTYRPVV---TPDGGSIIIKSTSAPAS 340
WP_088886144_1  VHLHVEDNAVSNVNLEFQG--T-----ELSDSKSMG-FLYPGDS-FVVESTVSGRVYGS----DQVTVSGTVKITYTPSS---GQI-----------PNS 295
WP_088856136_1  VYVKNNMDDSTPVEVLVEG--T-----KLSDYQ-SI-FIYPKST-ITVTSVPSGKVYGD----KWVTVSGTIKVTYTLQK---DTQ-----------E-- 281
WP_015857921_1  IDYTVSGGQVVVYNVTFNT--G-----SEEQAVTPG-ILDPGSG--TIESGNLTTSVGVLDGSLKVKVSGIVTITYRPVT---GPGGENVVIKSTSSVES 366
WP_014733996_1  IVLVAESNIVKNIRVEIKD-SS-----GDKDEKYID-IIYPQDGETSIVSKEFTLNIPDNS--KKIRISGNVMVTYTPGC---GNL-----------PTS 492
WP_088886109_1  VSFKAEDNAVNNIRIKVKDYTA-----RDSDSGTVS-FLNAGES-YTWKSSRFT-YTHSSG--GELNLSGEVEITYTPSC---GAV-----------PLL 315
WP_088882563_1  VHYHVENNAVSNISVKFKG--A-----YLEDSKSMA-FLSPGEG-GIITSGESE-FISGS---GEIQVMGVVKITYTSSP---GPV-----------PTN 492
WP_015859709_1  ----------MIDRVKFND-G------SESKVLSMDAIVYTGPG--SVESPEFIAPVGN-A--LQASVSGIVKIRYRSVTSPGGDT-----------PNS 67
WP_014122675_1  ------------MTITIND-KLADYYFGERPWDPIDRWVFKGSS--KNRFEGY---------------SYLIKVKF------------------------ 46
WP_014122677_1  --------DPGKVDLKIQA--L-----KISEGK-VK-YV--------------ARISNS----NSVPIRGTLTFYFTILD---GDH-----------L-- 158
WP_056934707_1  --------------------QT-----VQLDELALR-ALSVGDE-------------------TELQVSGEIIVESVTPS---E---------------- 445
WP_068320152_1  ----------------------------MREGNKFE-VLYPGLN-------------------------------------------------------- 15
WP_082781392_1  ---------------------------------------------------------------------------------------------------- 13
WP_010884703_1  ITMEAHNNDVRDIEIEVDDTTS-----GSSDKGSVD-YLTMGES-KTWFSKQFSIKPSD----TTVKIEGKIKITYTPVC---GDI-----------PQR 460
WP_062388971_1  VSMKAKNNVVTDVRISVHG--T-----GLSDSGEIG-MIHPDDGEVDWTSSISDKVRGS----NQVIVSGKVKITYTSNS---GPT-----------PNS 491
WP_074631387_1  ---------------------------------------------------------------------------------------------------- 15
WP_010884704_1  ---------------------------------------------------------------------------------------------------- 0
WP_056934708_1  ---------------------------------------------------------------------------------------------------- 0
WP_048151448_1  ---------------------------------------------------------------------------------------------------- 0
WP_088863691_1  ---------------------------------------------------------------------------------------------------- 0
WP_048810998_1  ---------------------------------------------------------------------------------------------------- 0
WP_048148049_1  ---------------------------------------------------------------------------------------------------- 311
WP_055428947_1  ---------------------------------------------------------------------------------------------------- 16
WP_099209381_1  ---------------------------------------------------------------------------------------------------- 0
WP_014122994_1  ---------------------------------------------------------------------------------------------------- 55
WP_048165381_1  ---------------------------------------------------------------------------------------------------- 0
WP_048165382_1* ---------------------------------------------------------------------------------------------------- 0
WP_068319940_1  ---------------------------------------------------------------------------------------------------- 0
WP_068320169_1  ---------------------------------------------------------------------------------------------------- 0
WP_068320161_1  ---------------------------------------------------------------------------------------------------- 58
WP_048055992_1  ---------------------------------------------------------------------------------------------------- 0
WP_074631389_1  ---------------------------------------------------------------------------------------------------- 0
WP_014012910_1  ---------------------------------------------------------------------------------------------------- 0
WP_015857844_1  ---------------------------------------------------------------------------------------------------- 24
WP_099209388_1  ---------------------------------------------------------------------------------------------------- 0
WP_088865600_1  ---------------------------------------------------------------------------------------------------- 0
WP_048165388_1  ---------------------------------------------------------------------------------------------------- 0
WP_048055998_1  ---------------------------------------------------------------------------------------------------- 0
WP_088863688_1  ---------------------------------------------------------------------------------------------------- 0
WP_048151737_1  ---------------------------------------------------------------------------------------------------- 4
WP_048055995_1  ---------------------------------------------------------------------------------------------------- 0
WP_055430151_1  ---------------------------------------------------------------------------------------------------- 0
WP_048150566_1  ---------------------------------------------------------------------------------------------------- 0
WP_099209386_1  ---------------------------------------------------------------------------------------------------- 0
WP_088886110_1  ---------------------------------------------------------------------------------------------------- 196
WP_068320165_1  ---------------------------------------------------------------------------------------------------- 0
WP_055428943_1  ---------------------------------------------------------------------------------------------------- 0
WP_014122673_1  ---------------------------------------------------------------------------------------------------- 0
WP_088856137_1  ---------------------------------------------------------------------------------------------------- 177
WP_048152596_1  ---------------------------------------------------------------------------------------------------- 0
WP_048151739_1  ---------------------------------------------------------------------------------------------------- 0
WP_088865604_1  ---------------------------------------------------------------------------------------------------- 0
WP_088865603_1  ---------------------------------------------------------------------------------------------------- 0
WP_048150577_1  ---------------------------------------------------------------------------------------------------- 0
WP_055430149_1  ---------------------------------------------------------------------------------------------------- 0
WP_068320157_1  ---------------------------------------------------------------------------------------------------- 6
WP_014788172_1* ---------------------------------------------------------------------------------------------------- 0
WP_048165379_1  ---------------------------------------------------------------------------------------------------- 0
WP_068320171_1  ---------------------------------------------------------------------------------------------------- 0
WP_088865607_1* ---------------------------------------------------------------------------------------------------- 0
WP_058939190_1* ---------------------------------------------------------------------------------------------------- 0
WP_068319946_1  ---------------------------------------------------------------------------------------------------- 0
WP_048150353_1  ---------------------------------------------------------------------------------------------------- 0
WP_048150344_1* ---------------------------------------------------------------------------------------------------- 56
```

```
WP_048055999_1  ---------------------------------------------------------------------------------------------------- 0

WP_058939188_1  FKDGQ----------TLSR---ECQDRTI-TRSYSETIDLGFS--LDWSKVDVIVTA----SR----HNIVEGESVTYTVKVKNGNSLPVDG-------V 636
WP_014788798_1  ESSLN----------PDDTPP-TYSTLQA-VEEYSSTIKLQYDP-TKNIDVEVMTTP----QS----GDIKENDPVQFKIKVSNHNPFSITGSYYLKVEV 587
WP_048151755_1  SREDA----------PILS---SCSARTI-TEQYSATFDLS-SS-VDWNLVGIKIET----SS----DDITEGDSVTYKVVVENKNNVALNG-------V 591
WP_088181398_1  KNE------------PFSASL-SCQERTI-TKDYSATIDLTSP--VDWSKVNIRVEA----SR----DSITKGESVTYRVIVENRQSVKLDD-------V 592
WP_048165390_1  KNHE-----------TLSTSP-SCQERTI-TREYSSTINLESP--IDWSKVSIRMET----SR----DRIVEGESVTYRVVVENGNSAPLSD-------V 588
WP_088865610_1  KNHE-----------TLSTSP-NCQERTI-TREYSSTINLESP--VDWNKVRIRIEA----SK----ESVTAGESVTYRVVVENGNSVPLDD-------V 578
WP_014012907_1  ESGLN----------LDDTSS-KYSTRQA-VEEYSSTIKLQYDP-TKNIDVEVTTIP----QS----RNIEEGDSVQFKIKVSNLNPFSVTGNYSLKVEV 593
WP_099209391_1  GFNAS----------AIGAGI-RNVTV---TEDYHKVINLKSS--IDPSKVTFSVFT---DPA----SPVLVGTNVKFKIYVYNGNSKPVSFNYRLYVQC 562
WP_088863686_1  AGSEP----------TGGTKE-MEI-----TKEYSTTIKLDDV--IDPNKITVNVIP----GE----TTIHEGDNVVFKVHVFNGNGKALSGTCTLRATY 542
WP_068319935_1  ---------------TLSITS-QSSEEKVKIVEYSKDFELEEHD-IDPKNVSVKLVV----DP----NPARTWENVKLKLKIRNDNEKSISGVYQVKIAM 524
WP_011013125_1  IQT-----------------TSMSKKTTERTYEETISLKDP--VDPSKVSVTLSI-----D----GDRVEGSALTFKARVRNDNEVGISGSCTLSLEV 527
WP_042692137_1  NPSQP----------TVSSSS-SSSDVQVAVKSYSKTITANLS--IGPSKVFFEIVP----SK----TEITEGSSVSFTIKIRNENDVPISGQWSFHASY 575
WP_052696221_1  ASS----------------P-ETSMKLI-EVPYSGTITLN-DP-VDPQKVNVSIIPIDEDGD----STILEGESVKFMVKILNENGEFVKGNCYIDISY 541
WP_014122995_1  IGEPS----------ISGYSDDITAIRVI-KLEYTGTFDLISQ--LDPEKVKVKVIP----SK----DVAEVGDSITFKLRIKNGNTLPIRGYYTLYAQV 419
WP_088886144_1  VS-----------------P-ANDEREI-TESYSKTIPLEEG--IDPSKVYVSVLAQDENGD----GTVKTGEDVTFKVVVRNDNDVNIAGTCTIRIVY 369
WP_088856136_1  ---------------PNNVGP-TSEKREI-IKSYSKTIHLE-D--IDPNKIYLDIKP----QDANHDGIIETGEDVTFKIIVRNDNKANIGGTCTLNVEY 357
WP_015857921_1  VSEPA----------TLDYGDETTSIRVI-KLEYTGTFDLISQ--LDPEKVKVKVMP----SK----DVAEVGDSITFRLRIENGNTLPVRGYYTLYAQV 445
WP_014733996_1  IKDL---------------A-ACQDRTT-TREYSEIIDISRDPEIEYDKVKFEVVP----SP----STVEEGKTVLFKIKVKNDNSQGFTARYSLNIRL 566
WP_088886109_1  QESPE----------ASSTSP-SCGDRTI-TKDYSATANLT-SP-VDWSKVNVRVEA----SR----TSITEGESVTYRVIVENNQNVELDG-------V 386
WP_088882563_1  NSTG-----------------RKQEMEI-IREYSATIKLE-DA-IDPNKISVSI-P----GE----VEINEGDSVPFTVIVYNYNRKPVTGTCTLVATY 562
WP_015859709_1  KPGDPVETTSVQAYTPADYST-PTAPVLV-MESYHGTLDLISD--IDLSKITVKTIA----NT----TKVTEGQSVGFAVRLRNDNVKAVNGVLSLFVTV 155
WP_014122675_1  --SNGIAVSDV--------------------------FTVKEQ--TRLEKI-VEAGK----NA----TKITEGQSVGFAVRLRNDSPKAVNGTLSFFAAV 107
WP_014122677_1  ---------------PRQV---------------------------------------------VNHT-----------RVVIMPNSQISV---------- 178
WP_056934707_1  ---------------------------------------------------------------------------------------------------- 445
WP_068320152_1  -------------------PL-GCDKEKTIVERYSEDFEVKKQE-VDTDKIELSIYP----SP----EHPKTFQDVTFYVTITNDNDKEISGKCTVYIAL 86
WP_082781392_1  ------------------------------ILQYSTTIDLKED--INPAKVTVTVQP----SK----SQVKVDEPVYFTITVLNDNSKDVEGDYTFTIPC 73
WP_010884703_1  SGQ---------------SLD-ECTEPRTISDEYSKVIEVTDS--IDPSKVTFNIIA----SK----KKIREGDSVDFYIEVTNDNNIKIEGKYLLEVSV 534
WP_062388971_1  IRD------------------------------------------------------------------------------------------------- 494
WP_074631387_1  ------------------------------------------------------------AN----TGIAIGATALLTIAV------------------- 32
WP_010884704_1  ---------------------------------------------------------------------------------------------------- 0
WP_056934708_1  ----------------------------------------------------------------------------MLKL-------------------- 4
WP_048151448_1  ---------------------------------------------------------------------------------------------------- 0
WP_088863691_1  ---------------------------------------------------------------------------------------------------- 0
WP_048810998_1  ---------------------------------------------------------------------------------------------------- 0
WP_048148049_1  ---------------------------------------------------------------------------------------------------- 311
WP_055428947_1  ---------------------------------------------------------------------------------------------------- 16
WP_099209381_1  ---------------------------------------------------------------------------------------------------- 0
WP_014122994_1  ---------------------------------------------------------------------------------------------------- 55
WP_048165381_1  ---------------------------------------------------------------------------------------------------- 0
WP_048165382_1* ---------------------------------------------------------------------------------------------------- 0
WP_068319940_1  ---------------------------------------------------------------------------------------------------- 0
WP_068320169_1  ---------------------------------------------------------------------------------------------------- 0
WP_068320161_1  ---------------------------------------------------------------------------------------------------- 58
WP_048055992_1  ---------------------------------------------------------------------------------------------------- 0
WP_074631389_1  ---------------------------------------------------------------------------------------------------- 0
WP_014012910_1  ---------------------------------------------------------------------------------------------------- 0
WP_015857844_1  ---------------------------------------------------------------------------------------------------- 24
WP_099209388_1  ---------------------------------------------------------------------------------------------------- 0
WP_088865600_1  ---------------------------------------------------------------------------------------------------- 0
WP_048165388_1  ---------------------------------------------------------------------------------------------------- 0
WP_048055998_1  ---------------------------------------------------------------------------------------------------- 0
WP_088863688_1  ---------------------------------------------------------------------------------------------------- 0
WP_048151737_1  ---------------------------------------------------------------------------------------------------- 4
WP_048055995_1  ---------------------------------------------------------------------------------------------------- 0
WP_055430151_1  ---------------------------------------------------------------------------------------------------- 0
WP_048150566_1  ---------------------------------------------------------------------------------------------------- 0
WP_099209386_1  ---------------------------------------------------------------------------------------------------- 0
WP_088886110_1  ---------------------------------------------------------------------------------------------------- 196
WP_068320165_1  ---------------------------------------------------------------------------------------------------- 0
WP_055428943_1  ---------------------------------------------------------------------------------------------------- 0
WP_014122673_1  ---------------------------------------------------------------------------------------------------- 0
WP_088856137_1  ---------------------------------------------------------------------------------------------------- 177
WP_048152596_1  ---------------------------------------------------------------------------------------------------- 0
WP_048151739_1  ---------------------------------------------------------------------------------------------------- 0
WP_088865604_1  ---------------------------------------------------------------------------------------------------- 0
WP_088865603_1  ---------------------------------------------------------------------------------------------------- 0
WP_048150577_1  ---------------------------------------------------------------------------------------------------- 0
WP_055430149_1  ---------------------------------------------------------------------------------------------------- 0
WP_068320157_1  ---------------------------------------------------------------------------------------------------- 6
WP_014788172_1* ---------------------------------------------------------------------------------------------------- 0
WP_048165379_1  ---------------------------------------------------------------------------------------------------- 0
WP_068320171_1  ---------------------------------------------------------------------------------------------------- 0
WP_088865607_1* ---------------------------------------------------------------------------------------------------- 0
WP_058939190_1* ---------------------------------------------------------------------------------------------------- 0
WP_068319946_1  ---------------------------------------------------------------------------------------------------- 0
WP_048150353_1  ---------------------------------------------------------------------------------------------------- 0
WP_048150344_1* ---------------------------------------------------------------------------------------------------- 56
WP_048055999_1  ---------------------------------------------------------------------------------------------------- 0

WP_058939188_1  KYRVTVSLPQFDPKNYSGTVTIPANGEKTILT-KTVAYPDSGTYLARATISWNG----HTKSASDKVIV------------TTGTLVISDVDSPRNPDD 719
WP_014788798_1  PYIVSENIMESKFKEFTGSLSLQGFDDSTVYV-GSITYPKQGTYKYEGYFKVGQ----YYKKFNGTIEV-------KPRPDGESPRIKITPLDWPSTARR 675
WP_048151755_1  DYSVVIPYSDSGSWKYSGTVDVPANEEVTIIK-KTVTYEEAGTYVASASI-KWD---GNSKSVKKSVTV-------------NSETISMNVDFSPNNPTE 673
WP_088181398_1  KYSVTIPFSPTNSRTYSGTVDVQQDGEQVILE-RTVTYTDAGTYVVHASI-YWN---GHSKSAEKSVTV------------TSGTLSITGVDVSPVNPTH 675
WP_048165390_1  EYSISIPFSPTNSKTYSDTVNVPANGAVTLLE-QTITYEDASTYVAHASI-YWN---GNSKSASKSVTV------------TSGTLTITGVDVSPANPTH 671
WP_088865610_1  EYSISIPFSPTNSRTYSDTVNVPANGEKTLLE-QTVTYEDASTYVAHASI-YWN---GHSKTTSKSVTV------------TSGTLTITCVDISPATPTH 661
WP_014012907_1  PYMVENNLIESKFKEFTGSLSLQGFDDSTVYV-GSVTYPKHGTYKYEGYFKVGQ----YYKKFNGTIEVKPR-------PDDDSPSIKITPLDWPSTARR 681
WP_099209391_1  P----TSPGNSFTKTVTGSLSLGPNSGRTITV-FSNTFSSTGSFGYSGTLYFGQ----YSKTSQGTLDVRAT-------PVSGDSVKILGVSVSPNPAKV 646
WP_088863686_1  P----ETSTSVGTVQLNTEISVPAEDWVTETI-GTVNYPNSGTFGYSGECDFGN---GISKSFSGSVTVVRDDNPD---PPAQGALKIVSLTPEPDHARV 631
WP_068319935_1  P--------DSGTIEYTGSVTVNGGTEKVLLL-EPIEYSKPGMYSYLVSFKFGD----FLVMDNGSIKVIQDSG-----DSSSQTLEIMSVEFSPSIPRV 606
WP_011013125_1  P----TSGTSRSSYQKSESFNAEGKSTALVNF-GSVTYSRSGTYSYVLTCKFGQ----HSKSVDGEFTV---------RKKSSGSLGITTVTITPNLPRA 609
WP_042692137_1  K----DSSYKTHSFDRSGSVTVQAHDTVSISV-GPVVYPVHGTYGYSGIFKFGPNLMYSRDNSSEIVVVASSGTPSPSPTPSAGSLRIVSVSPEPAHPKA 670
WP_052696221_1  P----ITSTETSIAKFQSSFSVSAGGSVSETM-GELTYEYPGVFNYLGKC-YFN---QYSKTVSGSVTVISAERGS---RSISGAFGI-----YPATIYV 624
WP_014122995_1  P----DGPGKRTVKLGGDRVTLGANKETEITV-STVPYSEPGRYEYFAVFSYAG----LQATDRGSVLVQR--------EDDPGKVYIEGVEVNPDLPKE 502
```

```
WP_088886144_1  P----ISSSDTSARSFSVSVNAPAGGSATETF-GSVHYAWSGTFTYTGKCTFDQ----YEKSFSGYVTVEEDDST----DPTVVKMIDVVPTSWPSTARL 456
WP_088856136_1  P----ISSSDRETKDYITSINVPAGQVVEKEFEERTSYHWEGTFHYEGHCVFGQ----YFKNMTGSLTVVE--------DGISGGMDI-KVRHDPESPQE 440
WP_015857921_1  P----DGSGKRTVRLGGGKLTLGANKETEVTV-STVPYPEPGRYEYFAVFSYAG----FQAEDRGSVLVQR--------EDDPGKVYIEGVEVDPDLPKE 528
WP_014733996_1  P---------EGEKSCSGEIFIPGNGEFEKGT-CKINYSKPGTYGYLGELSYKN----YIWDYKGYITVIR--------ESPKGTIRINSILWEPEVPKV 644
WP_088886109_1  EYSVTIPFSPTNSRTYSGKVDVQPNGELVILE-KTVTYTDASTYTTHASI-YWN---GHSKSAETSVTVIS--------DQNDQKSINGAFSFSPSVVYI 473
WP_088882563_1  P----ETGTQVGTVRLNKEIGVGASGYVVERI-GNVSYSSPGTFGYSGKCDFGN---GISKSFSGRVKV------------DSRTVRIAGVDVSPETPVS 642
WP_015859709_1  P----EENSERLVLLGRKTVILNPDENVVIQV-GNVTYPQAGTYEYFATFSFKG----MEVKDSGSVEVRR--------LVLQKNLAIKSLQILPRNPEE 238
WP_014122675_1  P----EENSERLVLLNRKTVTLNPDENVVIQV-GNVTYLKAGTYEYFATFSFKG----MEVKDSGSVEVTR--------LVLQKNLAIKGLQIFPANPEE 190
WP_014122677_1  ---------DLGTVEYLGS----------------------GTYGFTGILDFGG----FEKEYGGEVTVLI--------PVDGGELKISKVEVGPVPIFE 235
WP_056934707_1  ---------------------------------------------------------------------------------------------------- 445
WP_068320152_1  P---------EGTGRYSKDVIVPASSSKTFEI-AKVRYGKPGTYGYGGVFKFGQ----FEVSASGSVTVSQDS------SGSSGSLKIKQVTFEPSLPKV 166
WP_082781392_1  ----------IGRRPIKGHTVSPAGSYGEDTI-GPITCPEHGEYGYHVTFYFGP----YNRTSGGRLVVVNRDNGGN--DDSSGSLKIKDARFSPETPKD 156
WP_010884703_1  ---------------------------------------------------------------------------------------------------- 534
WP_062388971_1  ---------------------------------------------------------------------------------------------------- 494
WP_074631387_1  -------------------------------------------------------------------------------TVTLGTLSITGVDVSPVNPTH 53
WP_010884704_1  -------------------------------M-ATVSYSKSGTYSYSGSFSFGQ---GYSKTDSGEIVVARRD------TGTTGSLKIAGLTYIPKNPRE 59
WP_056934708_1  --------------------------------------------------------------------VVR--------DKN------------------ 10
WP_048151448_1  ---------------------------------------------------------------------------------------------------- 0
WP_088863691_1  ---------------------------------------------------------------------------------------------------- 0
WP_048810998_1  ---------------------------------------------------------------------------------------------------- 0
WP_048148049_1  ---------------------------------------------------------------------------------------------------- 311
WP_055428947_1  ----------------------------------------------------------------------------------------GHLEVYPEWVEA 28
WP_099209381_1  ---------------------------------------------------------------------------------------------------- 0
WP_014122994_1  ---------------------------------------------------------------------------------------------------- 55
WP_048165381_1  ---------------------------------------------------------------------------------------------------- 0
WP_048165382_1* ---------------------------------------------------------------------------------------------------- 0
WP_068319940_1  ---------------------------------------------------------------------------------------------------- 0
WP_068320169_1  ---------------------------------------------------------------------------------------------------- 0
WP_068320161_1  ---------------------------------------------------------------------------------------------------- 58
WP_048055992_1  ---------------------------------------------------------------------------------------------------- 0
WP_074631389_1  ---------------------------------------------------------------------------------------------------- 0
WP_014012910_1  ---------------------------------------------------------------------------------------------------- 0
WP_015857844_1  ---------------------------------------------------------------------------------------------------- 24
WP_099209388_1  ----------------------------------------------------------------------------------------MTVEVKPT---- 8
WP_088865600_1  ---------------------------------------------------------------------------------------------------- 0
WP_048165388_1  ---------------------------------------------------------------------------------------------------- 0
WP_048055998_1  ------------------------------------------------------------------------------------------MEVRPT---- 6
WP_088863688_1  ---------------------------------------------------------------------------------------------------- 0
WP_048151737_1  ---------------------------------------------------------------------------------------------------- 4
WP_048055995_1  ---------------------------------------------------------------------------------------------------- 0
WP_055430151_1  ---------------------------------------------------------------------------------------------------- 0
WP_048150566_1  ---------------------------------------------------------------------------------------------------- 0
WP_099209386_1  -------------------------------------MGDDTMWAKMSRAGFWR---------------------------------------------- 17
WP_088886110_1  ---------------------------------------------------------------------------------------------------- 196
WP_068320165_1  ---------------------------------------------------------------------------------------------------- 0
WP_055428943_1  ---------------------------------------------------------------------------------------------------- 0
WP_014122673_1  ---------------------------------------------------------------------------------------------------- 0
WP_088856137_1  ---------------------------------------------------------------------------------------------------- 177
WP_048152596_1  ---------------------------------------------------------------------------------------------------- 0
WP_048151739_1  ---------------------------------------------------------------------------------------------------- 0
WP_088865604_1  ---------------------------------------------------------------------------------------------------- 0
WP_088865603_1  ---------------------------------------------------------------------------------------------------- 0
WP_048150577_1  ---------------------------------------------------------------------------------------------------- 0
WP_055430149_1  ---------------------------------------------------------------------------------------------------- 0
WP_068320157_1  ---------------------------------------------------------------------------------------------------- 6
WP_014788172_1* ---------------------------------------------------------------------------------------------------- 0
WP_048165379_1  ---------------------------------------------------------------------------------------------------- 0
WP_068320171_1  ---------------------------------------------------------------------------------------------------- 0
WP_088865607_1* ---------------------------------------------------------------------------------------------------- 0
WP_058939190_1* ---------------------------------------------------------------------------------------------------- 0
WP_068319946_1  ---------------------------------------------------------------------------------------------------- 0
WP_048150353_1  ---------------------------------------------------------------------------------------------------- 0
WP_048150344_1* ---------------------------------------------------------------------------------------------------- 56
WP_048055999_1  ---------------------------------------------------------------------------------------------------- 0

WP_058939188_1  GDRVRFDVS-----------------IRNPSSRSRSLNVKLFID-------GVKESSRTV--SIGG--NDEST------------TTLTW----TATAGN 775
WP_014788798_1  GNTVSFKVQ-----------------LDSTHHNDREARVELIID-------NELPPVQTVETSVKA--GSSTT------------VTLTWYVPSSIPLGE 737
WP_048151755_1  GDSVEFDVS-----------------LKNPTSTSRTVTVKLFID-------GVEKSSRTV--TLHG--GDSMI------------VTLTW----TAQAGE 729
WP_088181398_1  GDTVSFDVS-----------------LKNPVSRSRALTIKLFID-------GVEKSSKSL--TIGA--EGSRV------------VSLTW----TAQAGD 731
WP_048165390_1  GDSVSFDVS-----------------VKNPVSRSRSVMVKLFID-------DAEKASKTI--TIGS--GGSGV------------VSLTW----TAVAGT 727
WP_088865610_1  GDTVSFDVS-----------------VRNPISRSRIVTVKLFID-------GVEKSSKTI--TIGG--GSSKE------------VTLTW----AARAGE 717
WP_014012907_1  GNTVRFKVQ-----------------LDSTYKDDREAKIELIID-------DELSPVHTVETSVKG--GSSTM------------VTLSWNVPSAFPIGE 743
WP_099209391_1  GKPVKFTVK-----------------LKNNYKEKKNVDVSISVG-------SSWRKYRTV--GVPS--GGTVT------------VTFTW---VPSSAGD 703
WP_088863686_1  GDTVWFSVR-----------------VKSSYPSTRSLKIKLLID-------GELIKTVSG--SIDG--NSEKT------------FTLAW---KANKLGP 688
WP_068319935_1  SNLANVIVK-----------------VKT--STAKTVKVELMID-------GEKVASQEG----QV--NGERN------------FILPW---RPQASGE 659
WP_011013125_1  GEIVTFKVE-----------------VKNSYSSGRNVSVKLFID-------DVEVDSTSA--YIPP--DSTKT------------LTLQW----SAEGGE 665
WP_042692137_1  GDTVKFNVK-----------------IDSSYSSTQRALVKLYID-------DELKDSKEL---NVG--TSGAS------------VTLHW----LAVAGE 725
WP_052696221_1  GDVAMFHYT-----------------VIPLEDSISNPTVTLESD-------DN-----------------------------------------SILILK 659
WP_014122995_1  YETVNFTVT-----------------LGNTYPTAEDLKIALYID-------GRVVDEKNV--GISA--NSENK------------VVLRW---SSAEKGD 559
WP_088886144_1  GGSVEFSVD-----------------LKNTYPVDKSVKVELKID-------GTVVDVVEV--TVIA--GRSTG------------ILLHWNVPDDFPVGE 516
WP_088856136_1  DQVVKFVVD-----------------VSNNYASSKEVEVRLLID-------NTLIDSVKG--KVDP--QKERT------------FVLEW----SAVAGD 496
WP_015857921_1  YETVNFTVT-----------------LGNTYPTAEDLKIALYID-------GRVVDQKSV--EILA--NSENK------------VVLRW---YSAEKGD 585
WP_014733996_1  NRQVNFKVE-----------------VKNEYDTSKTLLVKLFID-------GKEIDSAQK--VVSG--NSAEE------------FSLTW---VPSTSGE 701
WP_088886109_1  GDTVTFHYV-----------------ITTSKGQLSDPTVTLELD-------NGQKIVEKT---------------------------------------- 509
WP_088882563_1  GDIVNFDVM-----------------IKNPFSVSKNVTVKLFID-------GNEKSRENL--WLGG--GSSEL------------VTLTW----TAQAGG 698
WP_015859709_1  HDDVDIIVYPYYVFRLVPESNVESSVFKNNFGPDALVPVSLNSEQGNIELSVVVSDVKTS--DIVF--GDSIKISPENIR---PFYVSHW---RNAKAGD 328
WP_014122675_1  HDDVVIIVYPYYVYSFVPDSVAKNTVFRNNFGPDALVPVSVNSEQEDLELSVVVRDVKTS--SLVF--GNSVKLDSGNIKNIDPFYVFRW---RNAKAGD 283
WP_014122677_1  SDSVEFNVT-----------------VVNSYRSTKDISLKLIVDGKYG---SELAYWVNG--TVES--HSMKT------------FTLYW----DGLEGS 295
WP_056934707_1  ---VEYKVK-----------------VTVKAENAQATNINIYID-------GNKEY-------------------------------------------- 474
WP_068320152_1  QDNVKVRVK-----------------VSNSYSSKKTVKVELVVD-------GKTVNQVDG--VVSG--GKERE------------FILSW---TPTGAGE 223
WP_082781392_1  GDTVTFQVK-----------------VYNNYTDDKNVKLTLYID-------GSEADSVAG--SVSA--GSTGE------------FSLHW----GAEAGE 212
WP_010884703_1  ---------------------------------------------------------------------------------------------------- 534
WP_062388971_1  ---------------------------------------------------------------------------------------------------- 494
WP_074631387_1  GDSVSFDVS-----------------IKNPVSRSRSVTVKLFID-------GVEKSSRSL--TIGG--GSTGS------------VALTW----TAQAGE 109
WP_010884704_1  GEFISFIVN-----------------VTNSYSSSQKAKVILYID-------KEKIGEASG--EVGV--NSWST------------FKLKW----KAKGGK 115
```

```
WP_056934708_1  -------------------------------------------S-------GEVVYSEEM--AITPEYLDDSE------------YTFTW---SNVKAGE 43
WP_048151448_1  ---------------------------------------------------------------------------------------------------- 0
WP_088863691_1  -------------------------------------------------------------------------------------------------MPP 3
WP_048810998_1  ---------------------------------------------------------------------------------------------------- 0
WP_048148049_1  ---------------------------------------------------------------------------------------------------- 311
WP_055428947_1  GDSVSVHIT-----------------VTNLDLNCAQGPFDIELV-------D------------------------------------------------ 56
WP_099209381_1  ---------------------------------------------------------------------------------------------------- 0
WP_014122994_1  ---------------------------------------------------------------------------------------------------- 55
WP_048165381_1  ---------------------------------------------------------------------------------------------------- 0
WP_048165382_1* ------------------------------------------------------------------------------------MVTLTW----AAVAGE 12
WP_068319940_1  ---------------------------------------------------------------------------------------------------- 0
WP_068320169_1  ------------------------------------MPLFFYLK-------GRIRDIA----------------------------VLDD---YSTEAGE 26
WP_068320161_1  ---------------------------------------------------------------------------------------------------- 58
WP_048055992_1  ---------------------------------------------------------------------------------------------------- 0
WP_074631389_1  ---------------------------------------------------------------------------------------------------- 0
WP_014012910_1  ---------------------------------------------------------------------------------------------------- 0
WP_015857844_1  ---------------------------------------------------------------------------------------------------- 24
WP_099209388_1  ---------------------------------------------------------------------------------------------------- 8
WP_088865600_1  ---------------------------------------------------------------------------------------------------- 0
WP_048165388_1  ---------------------------------------------------------------------------------------------------- 0
WP_048055998_1  ---------------------------------------------------------------------------------------------------- 6
WP_088863688_1  ---------------------------------------------------------------------------------------------------- 0
WP_048151737_1  ---------------------------------------------------------------------------------------------------- 4
WP_048055995_1  ---------------------------------------------------------------------------------------------------- 0
WP_055430151_1  ------------------------------------------------------------------------M------------VTLTW----TAQAGE 12
WP_048150566_1  ---------------------------------------------------------------------------------------------------- 0
WP_099209386_1  ---------------------------------------------------------------------------------------------------- 17
WP_088886110_1  ---------------------------------------------------------------------------------------------------- 196
WP_068320165_1  ---------------------------------------------------------------------------------------------------- 0
WP_055428943_1  ---------------------------------------------------------------------------------------------------- 0
WP_014122673_1  ---------------------------------------------------------------------------------------------------- 0
WP_088856137_1  ---------------------------------------------------------------------------------------------------- 177
WP_048152596_1  ---------------------------------------------------------------------------------------------------- 0
WP_048151739_1  ---------------------------------------------------------------------------------------------------- 0
WP_088865604_1  ---------------------------------------------------------------------------------------------------- 0
WP_088865603_1  ---------------------------------------------------------------------------------------------------- 0
WP_048150577_1  ---------------------------------------------------------------------------------------------------- 0
WP_055430149_1  ---------------------------------------------------------------------------------------------------- 0
WP_068320157_1  ---------------------------------------------------------------------------------------------------- 6
WP_014788172_1* ---------------------------------------------------------------------------------------------------- 0
WP_048165379_1  ---------------------------------------------------------------------------------------------------- 0
WP_068320171_1  ---------------------------------------------------------------------------------------------------- 0
WP_088865607_1* ---------------------------------------------------------------------------------------------------- 0
WP_058939190_1* ---------------------------------------------------------------------------------------------------- 0
WP_068319946_1  ---------------------------------------------------------------------------------------------------- 0
WP_048150353_1  ---------------------------------------------------------------------------------------------------- 0
WP_048150344_1* ---------------------------------------------------------------------------------------------------- 56
WP_048055999_1  ---------------------------------------------------------------------------------------------------- 0

WP_058939188_1  HNWRVEVWEGGKLEDSRS------GSIE-VNSDESDPSAPC--------------------------------------------------SN-GEYWTA 817
WP_014788798_1  HKVTVRVWSRDSTTSNSEYIADAITLSN-PDNPDDEKDHTI--------------------------------------------------NI-GGLFNI 785
WP_048151755_1  HDWRIEAWEDGKLED---------SRSG-TIRVISES-DPCP-------------------------------------------------EG-E-YWTA 767
WP_088181398_1  HEWRIEVWEGGKLEA---------SRSG-GITVSDPPTSAC--------------------------------------------------LS-GVPLEG 770
WP_048165390_1  HKYRIEVWEGGK---------------L-EASRSGGITVGG--------------------------------------------------AP-SDSFNV 760
WP_088865610_1  HDWRIEAWEDGKLED---------SRSG-TIRVISES-DPCP-------------------------------------------------EG-E-YWTA 755
WP_014012907_1  HSVKVKVMSRHVNSND--------PWDL-EASETKSITIQN--------------------------------------------------LN-D-RFGI 782
WP_099209391_1  YAWNVQAHTSNNG-----------NSIL-EDSRTGDVDVLG--------------------------------------------------PS-H-EFAV 739
WP_088863686_1  VNVITQLLYLNS------------DEEE-KPEDEKNTTVHV--------------------------------------------------SG-DNLFGR 724
WP_068319935_1  HEWVVYLYERVANN----------KYLQ-RNSESGKILVAS--------------------------------------------------AD-S-QFAI 696
WP_011013125_1  HSYEIKLYNQGV---------------K-VEEKKGELNVSS--------------------------------------------------VE-Y-GLSG 697
WP_042692137_1  HSYTVKVYRLVNG-----------QELW-EDSRSGKINIIS--------------------------------------------------GS-NTS--- 759
WP_052696221_1  KTYQGAVITRDK---------------------------------------------------------------------------------------- 671
WP_014122995_1  HSYRIEVNRTLGNV----------NLGK-VDTYSGNITAF------------------------------------------------------DGDFGV 594
WP_088886144_1  HSVKIQAFSRNLDSND--------SWNP-EDSETGSINVER--------------------------------------------------PK-DLLFGK 556
WP_088856136_1  HDWRIEVWEDGK---------------L-EASRSEGITVSG--------------------------------------------------APSNPLFNV 530
WP_015857921_1  HHYYIEVNRTLGDI----------NLGK-VDTYSGNITAF------------------------------------------------------DGDFGV 620
WP_014733996_1  HDVLVKVYEVIG------------NQKL-QRAERRDTVAVV--------------------------------------------------SG-REGFLP 737
WP_088886109_1  ---------------------------------------------------------------------------------------------------- 509
WP_088882563_1  HGWRIEVLEDG----------------------------------------------------------------------------------------- 709
WP_015859709_1  LEVTVGLISGTE---------------V-IDSKT--VRLHI--------------------------------------------------NE-GDGYTG 359
WP_014122675_1  YEVTVGLIRGTE---------------V-LDKKT--VYLHI--------------------------------------------------SE-GDDYTG 314
WP_014122677_1  YTYRIELYSYGG---------------Y-VDSKVGELLVLP--------------------------------------------------YSARDKLKV 329
WP_056934707_1  ---------------------------------------------------------------------------------------------------- 474
WP_068320152_1  YGWIVYLYEKSANN----------VYLK-KDSKSGRIEVTR--------------------------------------------------IN-S-PFSV 260
WP_082781392_1  HSYEVKLYSVDEKNG---------KEAL-EDSEDGSIEVSR--------------------------------------------------VV-N-GLSA 250
WP_010884703_1  ---------------------------------------------------------------------------------------------------- 534
WP_062388971_1  ---------------------------------------------------------------------------------------------------- 494
WP_074631387_1  HEYRIEVWESGK---------------L-EASRSGRLSVSG--------------------------------------------------AP-GSSFNV 142
WP_010884704_1  HYYRVVIYRVNGD-----------AEEY-EDGETGELEVAS--------------------------------------------------LD-Q-QFAV 151
WP_056934708_1  YTAEAKVLYNGE---------------V-KDSEIRDLTVE----------------------------------------------------A-DNDFTG 74
WP_048151448_1  ----MQTWLED-------------------RSLGGKLEVSKRRSVPAVSTRGFSLQWTAVQGEHSYGVKLFEVLGGQELEVDSREGEVSVASK-NQLFAV 76
WP_088863691_1  AIGPTRVWCIT-------------------RTRPGKREECGELDVGNHNWRVEVLDGT--------------------LKASKSGSLSVSKQA-ANLFDV 63
WP_048810998_1  ---------------------------------------------------------------------------------------------------- 0
WP_048148049_1  ---------------------------------------------------------------------------------------------------- 311
WP_055428947_1  ---------------------------------------------------------------------------------------------------- 56
WP_099209381_1  ---MVDTWEWD-------------------NAFPRAVNKCVR-----------------------------------------------CYGL-SHEFAV 30
WP_014122994_1  ---------------------------------------------------------------------------------------------------- 55
WP_048165381_1  ---------------------------------------------------------------------------------------------------- 0
WP_048165382_1* HEWRIEVWESEKLEA---------SKSG-SIRVNSNGDSQK--------------------------------------------------SG-SNTINA 51
WP_068319940_1  ---------------------------------------------------------------------------------------------------- 0
WP_068320169_1  HGWIVYLYEKIAN-----------NRFVRRDSADGIVEVSE--------------------------------------------------E--NEGMSG 63
WP_068320161_1  ---------------------------------------------------------------------------------------------------- 58
WP_048055992_1  ---------------------------------------------------------------------------------------------------- 0
WP_074631389_1  ---------------------------------------------------------------------------------------------------- 0
WP_014012910_1  ---------------------------------------------------------------------------------------------------- 0
```

```
WP_015857844_1  ---------------------------------------------------------------------------------------------------- 24
WP_099209388_1  ---------------------------------------------------------------------------------------------------- 8
WP_088865600_1  ----MKKSYNSN---------------L-QQ--------------------------------------------------------------------- 11
WP_048165388_1  ---------------------------------------------------------------------------------------------------- 0
WP_048055998_1  ---------------------------------------------------------------------------------------------------- 6
WP_088863688_1  ---------------------------------------------------------------------------------------------------- 0
WP_048151737_1  ---------------------------------------------------------------------------------------------------- 4
WP_048055995_1  ---------------------------------------------------------------------------------------------------- 0
WP_055430151_1  HEYRIEAWESGK---------------L-EASRSGSV--------------------------------------------------------------- 33
WP_048150566_1  ---------------------------------------------------------------------------------------------------- 0
WP_099209386_1  ---------------------------------------------------------------------------------------------------- 17
WP_088886110_1  ---------------------------------------------------------------------------------------------------- 196
WP_068320165_1  ---------------------------------------------------------------------------------------------------- 0
WP_055428943_1  ---------------------------------------------------------------------------------------------------- 0
WP_014122673_1  ---------------------------M-EN--------------------------------------------------------------------- 3
WP_088856137_1  ---------------------------------------------------------------------------------------------------- 177
WP_048152596_1  ---------------------------------------------------------------------------------------------------- 0
WP_048151739_1  ---------------------------------------------------------------------------------------------------- 0
WP_088865604_1  ---------------------------------------------------------------------------------------------------- 0
WP_088865603_1  ---------------------------------------------------------------------------------------------------- 0
WP_048150577_1  ---------------------------------------------------------------------------------------------------- 0
WP_055430149_1  ---------------------------------------------------------------------------------------------------- 0
WP_068320157_1  ---------------------------------------------------------------------------------------------------- 6
WP_014788172_1* ---------------------------------------------------------------------------------------------------- 0
WP_048165379_1  ---------------------------------------------------------------------------------------------------- 0
WP_068320171_1  ---------------------------------------------------------------------------------------------------- 0
WP_088865607_1* ---------------------------------------------------------------------------------------------------- 0
WP_058939190_1* ---------------------------------------------------------------------------------------------------- 0
WP_068319946_1  ---------------------------------------------------------------------------------------------------- 0
WP_048150353_1  ---------------------------------------------------------------------------------------------------- 0
WP_048150344_1* ---------------------------------------------------------------------------------------------------- 56
WP_048055999_1  ---------------------------------------------------------------------------------------------------- 0

WP_058939188_1  --WLE-----VSPT-KMVGEG---------------K--VHVKIMASY---------------------------------------------------C 841
WP_014788798_1  --KLL-----AYPT-ELEGGG---------------E--VYLQVKAWN----------------YDG----------------SL------I------PV 816
WP_048151755_1  --WLE-----VSPT-EMVGEG---------------K--VHVKVMASY---------------------------------------------------C 791
WP_088181398_1  --HLE-----VYPE-GVEAGD---------------S--VSVHITVTNLDPNCAQGPFNIELVDDSG----------------AL------W------WP 817
WP_048165390_1  --VLT-----AYPT-ELEGSG---------------T--VYFTIKAWN----------------YGN----------------TA------I------PL 791
WP_088865610_1  --WLG-----VSPT-EMVGEG---------------K--VHVKVMASY---------------------------------------------------C 779
WP_014012907_1  --KLV-----AYPT-ELEGGG---------------E--VYLQVKAWN----------------YDG----------------SM------I------PV 813
WP_099209391_1  --KLE-----ATPT-ELKGGG---------------T--VYFTVKAWN----------------FAS----------------DG------L------NV 770
WP_088863686_1  --LVP-----NVAR-EVTQGT---------------T--VHFDVIVEN----------RGNYPAYDV----------------PI------R------VY 761
WP_068319935_1  --RLY-----ASPR-ELNGGG---------------E--VIFSVKVWN----------------YGN----------------DR------V------SL 727
WP_011013125_1  --WLE-----VVPN-PIEAGN---------------N--VSVIITVKN------SENYKQTLPIELV----------------DD------T------GN 738
WP_042692137_1  ---------------------------------------------------------------------------------------------------- 759
WP_052696221_1  ---------------------------------------------------------------------------------------------------- 671
WP_014122995_1  --SFT-----VWPR-TLDGGG---------------K--VWFTVKVRN----------------YRP----------------DK------L------DV 625
WP_088886144_1  --LVP-----NVNG-TVVQGT---------------V--VHFDVIVEN----------KEDYPAYNV----------------PI------R------VF 593
WP_088856136_1  --EWE-----VYPQ-ILYGGG---------------T--VFFKIYAWN----------------FME----------------NP------L------NV 561
WP_015857921_1  --SFT-----VWPR-TLDGGG---------------R--VWFTLKLRV----------------YHS-----------------K------Y------DF 650
WP_014733996_1  --KLE-----AFPQ-ELEGGG---------------E--VTFLVEVWN---------------------------------------------------- 760
WP_088886109_1  ---------------------------------------------------------------------------------------------------- 509
WP_088882563_1  ---------------------------------------------------------------------------------------------------- 709
WP_015859709_1  --RFLTGESQAHNL-TVTQGTFMNITLAADYYGELKN--EYVPVKVWYL---------------YDS----------------PS------E------GI 411
WP_014122675_1  --RFLTGGSQANHL-TVTQGTFLNITLAADYYGKLKN--EYVPITVRYT---------------YQS----------------PR-------------GI 365
WP_014122677_1  LGRLE-----LSPS-TAYIGK---------------TEKVAVKIKLWD----------------TRP----------------KS------I------GI 364
WP_056934707_1  ---------------------------------------------------------------------------------------------------- 474
WP_068320152_1  --RLY-----AYPT-ELEGGG---------------E--VTFLVKVWN----------------YGN----------------DR------V------SL 291
WP_082781392_1  --WFE-----VVPN-PVEAGN---------------N--VTVYITVKN------SENYKQTWPIELV----------------GN------T------GN 291
WP_010884703_1  ---------------------------------------------------------------------------------------------------- 534
WP_062388971_1  ---------------------------------------------------------------------------------------------------- 494
WP_074631387_1  --VLT-----AFPR-ELEGGG---------------E--VYLQVKAWN----------------YDG----------------SL------I------PV 173
WP_010884704_1  --SLD-----VFPA-ELEGGG---------------R--VFFTVGLRN----------------FDY----------------NA------I------PV 182
WP_056934708_1  --ILS-----ANITNDVSVGV---------------K--ARFTLTVRN----------------DEN----------------APRYSIPVR------VY 112
WP_048151448_1  --VMR-----AFPT-ELEGGG---------------T--VYLTVKVRN----------------FDA----------------GA------L------SL 107
WP_088863691_1  --ALT-----AWPV-ELEGGG---------------L--VFLQVHVWN----------------HAN----------------SA------I------SV 94
WP_048810998_1  ------------------------------------M--VYFTVKVRN----------------YRP----------------DK------L------DV 18
WP_048148049_1  ---------------------------------------------------------------------------------------------------- 311
WP_055428947_1  ----------------------------------------------------------------DSG----------------AL------W------WP 64
WP_099209381_1  --KLE-----VTPT-ELKGGG---------------T--VYFTVKAWN----------------FAS----------------DG------L------NI 61
WP_014122994_1  ---------------------------------------------------------------------------------------------------- 55
WP_048165381_1  ---------------------------------------MYFTIKAWN----------------YGN----------------TA------I------PL 17
WP_048165382_1* --NLEITDFACMPK-EVGLYEWRLDRD---------K--VSCTVTIQNKGPEIQIAPRVV--IDDIE--------------------------------- 102
WP_068319940_1  ---------------------------------------------------------------------------------------------------- 0
WP_068320169_1  --KLV-----VGNR-E---GS---------------E--VTFDVIVEN----------------RGQ----------------EI------VYYPVVRVY 97
WP_068320161_1  ---------------------------------------------------------------------------------------------------- 58
WP_048055992_1  ---------------------------------------------------------------------------------------------------- 0
WP_074631389_1  ---------------------------------------------------------------------------------------------------- 0
WP_014012910_1  ---------------------------------------------------------------------------------------------------- 0
WP_015857844_1  ---------------------------------------------------------------------------------------------------- 24
WP_099209388_1  ---------------------------------------------------------------------------------------------------- 8
WP_088865600_1  ---------------------------------------------------------------------------------------------------- 11
WP_048165388_1  ---------------------------------------------------------------------------------------------------- 0
WP_048055998_1  ---------------------------------------------------------------------------------------------------- 6
WP_088863688_1  ---------------------------------------------------------------------------------------------------- 0
WP_048151737_1  ---------------------------------------------------------------------------------------------------- 4
WP_048055995_1  ---------------------------------------------------------------------------------------------------- 0
WP_055430151_1  ---------------------------------------------------------------------------------------------------- 33
WP_048150566_1  ---------------------------------------------------------------------------------------------------- 0
WP_099209386_1  ---------------------------------------------------------------------------------------------------- 17
WP_088886110_1  ---------------------------------------------------------------------------------------------------- 196
WP_068320165_1  ---------------------------------------------------------------------------------------------------- 0
WP_055428943_1  ---------------------------------------------------------------------------------------------------- 0
WP_014122673_1  ---------------------------------------------------------------------------------------------------- 3
WP_088856137_1  ---------------------------------------------------------------------------------------------------- 177
```

```
WP_048152596_1   ---------------------------------------------------------------------------------------------------- 0
WP_048151739_1   ---ME-----VYPK-WVEAGD---------------S--VSVYITVTN----------------LDPNCAHGPFNIELVDDSGAK------W------WP 46
WP_088865604_1   ---------------------------------------MTVHITVTN----------------LDPKCAQGPFNIKLVDDSGAK------W------WP 33
WP_088865603_1   ---------------------------------------------------------------------------------------------------- 0
WP_048150577_1   ---------------------------------------------------------------------------------------------------- 0
WP_055430149_1   ---------------------------------------------------------------------------------------------------- 0
WP_068320157_1   ---------------------------------------------------------------------------------------------------- 6
WP_014788172_1*  ---------------------------------------------------------------------------------------------------- 0
WP_048165379_1   ---------------------------------------------------------------------------------------------------- 0
WP_068320171_1   ---------------------------------------------------------------------------------------------------- 0
WP_088865607_1*  ---------------------------------------------------------------------------------------------------- 0
WP_058939190_1*  ---------------------------------------------------------------------------------------------------- 0
WP_068319946_1   ---------------------------------------------------------------------------------------------------- 0
WP_048150353_1   ---------------------------------------------------------------------------------------------------- 0
WP_048150344_1*  ---------------------------------------------------------------------------------------------------- 56
WP_048055999_1   ---------------------------------------------------------------------------------------------------- 0

WP_058939188_1   NA--------L--PDVGGG-KINLLYLE--G-SVY------LDGKEIH---TFD-TN---TNGNYLLVD---ETKVIE------------EFDWPVSVG- 898
WP_014788798_1   KG--------F--IEVDGD-VIKNIEAK--I-PAS------ANGDQIL---KLR-YN---FA-------------GVG------------THTIKVFLD- 863
WP_048151755_1   SA--------L--PDVGGD-TIDLLYLG--G-SVY------LDGKEIH---SFD-TN---ANGNYLLVG---RTRVID------------EFDWPVSVG- 848
WP_088181398_1   KK--------D--MDYGCS-GCGYVIQE--R-ALK------IYAGKTM---SEW-TD---FNNIDQATT---LYLKVG------------GKTLASAKI- 874
WP_048165390_1   SG--------F--VEDEDG-AVVKRIDGFAG-RLP------ANAQNYT---LTA-FS---LD-----------VYGIG------------NHTFKLFLD- 842
WP_088865610_1   SA--------L--PDVGGD-TIDLLYLG--G-SVY------LDGREIH---SFD-TN---ANGNYLLVG---KTRVID------------EFDWPVSVG- 836
WP_014012907_1   RG--------F--IEINGK-RENIDAK---I-PIS------ANGDPII---TLR-RN----------------IVGVG------------VHTIKVFLD- 859
WP_099209391_1   HF--------K--VTDGDG-AVIKEFN---R-FVP------KGAGNYS---VNV-FS---LK-----------VYGVG------------KHTYTLTAS- 818
WP_088863686_1   YA--------Y--QTPEGE-FSDSIVFE--R-SPR------EIEGGAQ---YYA-FN---SIWSDGFNI---TLSNVG------------AYYFTLYVN- 818
WP_068319935_1   KG--------F--VSDGDG-AVVKNIDW--T-GVP------ANAQNYT---VTT-FT---LT-----------VYGVG------------EHKYKLFLD- 776
WP_011013125_1   VW--------W--PHEDYY-GVNYSISN--G-YLT------IYANKTA---RIT-AT---IGPLTQNTT---LILKIG------------GRQMASEDV- 795
WP_042692137_1   ---------------------------------------------------------------------------------------------------- 759
WP_052696221_1   ------------------------------------------YLEGYA---YYK-FD---RAGTYRITL---KVNGLP------------IFSKNIKVN- 706
WP_014122995_1   RG--------F--IEDEDG-AVIVKMGG--S-GVYTIPARREKSF------SFS-YTV----------------YGVA------------NHTFKLFID- 675
WP_088886144_1   YT--------Y--QTPNGG-FSDPIRVFERN-PRQ------IDGLGVN---LTW-FN---MT-----------LTNLG------------KYYFTLYVN- 644
WP_088856136_1   SFTSADTIHFK--VITNEE-TVIKEFN---S-YVP------EGGVKYN---VAN-FT----YT----------FYGVG------------NYTFILTAD- 617
WP_015857921_1   KD--------N--SNDPLP-DILMSTSP--T-VTYHIKIVDQNGIVIK---NFN-YDIPDKAGVYTLWISGPILYRTG------------NYTFKLVVDD 720
WP_014733996_1   ---------------------------------------------------------------------------------------------------- 760
WP_088886109_1   -----------------------YYGVT--I-SPD------SHLEDRT---TYV-FN---KAGTYRITL---KVNGLP------------IYSKTIEVK- 554
WP_088882563_1   ---------------------------------------------------------------------------------------------------- 709
WP_015859709_1   HWTSPIRV--F--EKD-IK------FDN--V-PPT------YVEGQYAQEFRGW-FG---FH-----------FDNPG------------VYHFYLYVN- 463
WP_014122675_1   FYSG-VWV--F--NRNGIK------FND--L-PLV------WVGNQYAFEFRDW-FG---LH-----------FNDPG------------MYYLYFDVN- 417
WP_014122677_1   -WEGHDV---E--IVDERG-VVWWPYK---G-RAF------EGPRDVK---TGEGYM-----YTDGILR---LHYGFNATLSTLLPVVNHNVTFYLEVD- 435
WP_056934707_1   ------------------------------G-PFD------LGAGESI---TKT-LP---VG-------------------------------------- 492
WP_068320152_1   KG--------F--VSDGDG-AIVKNINW--T-GVP------ANAQNYT---VTT-FT---LT-----------VYGVG------------EHKYKLFLD- 340
WP_082781392_1   VW--------W--PREDYY-GMNYSISD--G-DLT------IYANKTA---RIT-AT---IGPITQNTT---LTLKIG------------GYQMASKYV- 348
WP_010884703_1   ---------------------------------------------------------------------------------------------------- 534
WP_062388971_1   ---------------------------------------------------------------------------------------------------- 494
WP_074631387_1   KG--------F--IEVDGD-RIKNIETK--I-PAN------ANGDSIL---ALS-YN----------------LVGVG------------THSIKLFLD- 220
WP_010884704_1   RG--------F--VVDEDG-AIIKNIED------R------IPAKGVK---NIT-FS---YL-----------VYGVG------------NHTFKLFLD- 228
WP_056934708_1   YV--------Y--TSPDGVWHSVINVKE--A-EVD------FDPFGEY---QGW-FD---FT-----------FANPG------------TYKFYLVVN- 162
WP_048151448_1   SG--------F--VEDESG-AIIKRI-D--GFEGR------IPANGVK---NIT-FG---YT-----------VYGVG------------AHAFRVFLD- 156
WP_088863691_1   KG--------F--IEDEEG-AVIKRI-D--GYEGR------IPAKGYK---NLT-FT---YN-----------LAGVG------------NHTFKLFLD- 143
WP_048810998_1   RG--------F--IEDEAG-AVIVKMGD--SGVYT------IPAREEK---SFS-FS---YT-----------LYGIA------------NHTFKLFID- 68
WP_048148049_1   ---------------------------------------------------------------------------------------------------- 311
WP_055428947_1   RK--------D--MDYGCS-GCGYIIQN--G-ALK------IYAGKTM---SEW-TD---FNNIGQATT---LYLKVG------------GKPLASAKI- 121
WP_099209381_1   HF--------K--VTDGEG-AVVKEF-N--RFVPK-------GAGNYS---VDA-FS---LK-----------VYGVG------------KHTYTL---- 106
WP_014122994_1   ---------------------------------------------------------------------------GVA------------NHTFKLFID- 67
WP_048165381_1   SG--------F--VEDEDG-AVVKRIDGFAG-RLP------ANAQNYT---LTA-FS---LD-----------VYGIG------------NHTFKLFLD- 68
WP_048165382_1*  ---------------------------------------------------------------------------------------------------- 102
WP_068319940_1   --------------------------------------------MERK---SE---------------------CSDG------------ILLYKLFLD- 19
WP_068320169_1   YS--------YEAPDGEFG-KPILVFER--Q-PKE------LFPGETY---KDW-FK---MT-----------LTNPG------------KHHFTLYVN- 148
WP_068320161_1   ---------------------------------------------------------------------------------------------------- 58
WP_048055992_1   ----------------------------------------------------------------------------MG------------NHTFKLFLD- 11
WP_074631389_1   -----------------------MKTGD--Q--------------------------------------------------------------------- 6
WP_014012910_1   ---------------------------------------------------------------------------------------------------- 0
WP_015857844_1   ---------------------------------------------------------------------------------------------------- 24
WP_099209388_1   ---------------------------------------------------------------------------------------------------- 8
WP_088865600_1   -------------------WRDGVE--------------------------------------------------------------------------- 17
WP_048165388_1   ---------------------------------------------------------------------------------------------------- 0
WP_048055998_1   ---------------------------------------------------------------------------------------------------- 6
WP_088863688_1   ---------------------------------------------------------------------------------------------------- 0
WP_048151737_1   ---------------------------------------------------------------------------------------------------- 4
WP_048055995_1   ---------------------------------------------------------------------------------------------------- 0
WP_055430151_1   ---------------------------------------------------------------------------------------------------- 33
WP_048150566_1   ---------------------------------------------------------------------------------------------------- 0
WP_099209386_1   ---------------------------------------------------------------------------------------------------- 17
WP_088886110_1   ---------------------------------------------------------------------------------------------------- 196
WP_068320165_1   ---------------------------------------------------------------------------------------------------- 0
WP_055428943_1   ---------------------------------------------------------------------------------------------------- 0
WP_014122673_1   -------------------W-------------------------------------------------------------------------------- 4
WP_088856137_1   ---------------------------------------------------------------------------------------------------- 177
WP_048152596_1   ---------------------------------------------------------------------------------------------------- 0
WP_048151739_1   EN--------D--TEYGDS-MRGYEITN--G-VLK------IYAGKTL---SEW-SK---PKPVTQNTV---LYLKIS------------GKV------- 97
WP_088865604_1   KK--------D--TEYGCS-GCGYIIQD--G-TIK------LYAGKTM---SEW-VN---FSKISQGTT---LYLKVG------------GKVLASTKI- 90
WP_088865603_1   ---------------------------------------------------------------------------------------------------- 0
WP_048150577_1   ---------------------------------------------------------------------------------------------------- 0
WP_055430149_1   ---------------------------------------------------------------------------------------------------- 0
WP_068320157_1   ---------------------------------------------------------------------------------------------------- 6
WP_014788172_1*  ---------------------------------------------------------------------------------------------------- 0
WP_048165379_1   ---------------------------------------------------------------------------MRR------------KYTYKLFLD- 12
WP_068320171_1   ---------------------------------------------------------------------------------------------------- 0
WP_088865607_1*  ---------------------------------------------------------------------------------------------------- 0
WP_058939190_1*  ---------------------------------------------------------------------------------------------------- 0
WP_068319946_1   ---------------------------------------------------------------------------------------------------- 0
WP_048150353_1   ---------------------------------------------------------------------------------------------------- 0
WP_048150344_1*  ---------------------------------------------------------------------------------------------------- 56
WP_048055999_1   ---------------------------------------------------------------------------------------------------- 0
```

```
WP_058939188_1  ---NHNIKLKIKNT----------------N---GLLDYIKTKT--D-SVSV----------R----V-----LPSEYSLELTEIS-CSN-LNF------ 946
WP_014788798_1  ---NHDGEPNGEGEEYWSEVRVEVKG----K---MASSYLDCTR--E-VYPG----------E----K-------VTCVASWNPHI--TL-QSL------ 920
WP_048151755_1  ---NHNITLKIKNI----------------S---GLLDYIKTKT--D-SVSV----------R----V-----SPLEYSLELTEIS-CSG-LNF------ 896
WP_088181398_1  ---NVKQD----------------------G---PIKASMECKP--T-VIPL----------D----G------STTCTVHFELKS-VDT-VTL------ 915
WP_048165390_1  ---NYDGKPNGAGEEHWSEVKVEVKP----W---KADLKLECDD--G-VVLG----------E----N---DVDDLDCTLYLLRTNPDDS-VQV------ 905
WP_088865610_1  ---NHNITLKIKNT----------------G---GLLDYIKTKT--D-SVSV----------R----G-----LPLEYPLELTEIS-CGN-LNF------ 884
WP_014012907_1  ---NHDGEPNGAGEEHWSEATVEVKSMNGTE---LKQVSFECDN--P-KFNW----------N----G-IEYKATLVCKAFVYNPT-------------- 921
WP_099209391_1  ---ISGEVEK-------STATVEVEPP---K---VQKASMDCSN--M-VLSA----------K----K-GNLVLDMTCEVYFTNPT---D-VGW------ 874
WP_088863686_1  ---GEIEDDVKITVEPN-------------G---SVAAWMECNP--E-FVSE----------N----D-----DSVTCNFKVKNLG-NAP-LNF------ 869
WP_068319935_1  ---NYDGQPNGKGEEHWSEVTVEVRPMNGTG---LKQISSECDD--V-YFDF----------K----D-HAYRATLSCRVYLHNSNSRDE-IRIRDIAVL 851
WP_011013125_1  ---KVLGRVN--------------------W---LELSRVSCND--L-VLKV----------G----GTLDYKGTATCVVELRNPT-RSG-VAI------ 844
WP_042692137_1  ------------------------------H---VEIISIECPE--K-VSIP----------G----E-------LWCSVIVNNPL-SAN-IDV------ 794
WP_052696221_1  ---NL-------------------------P---ELTLHLECSN--E-PVLL----------G----E------TEVCNIIVKKNTLSAD-VPY------ 745
WP_014122995_1  ---NTDGKPNGNGEEHWDEGTMEVNPV---G---NIIANMSCKP--A-VISY----------T----G------STSCRITLLNQG-DSD-VAI------ 735
WP_088886144_1  ---GEIEDEKIITVTSN-------------S---SVMAWMECED--T-IPLY----------D----E-------LSCHIHILNNS-SDD-VSI------ 693
WP_088856136_1  ---WGS-------ETHTRTARVEVKPT---G---NILASMDCNP--T-TVPL----------D----S------STTCTIHLGLKN-ADT-VTL------ 670
WP_015857921_1  FIYNEEGKPLGLGTEEQREAKVEVTP-------------------------------------------------------------------------- 746
WP_014733996_1  -----------------------------------------YNS--D-RINL----------K----G-------------------------------- 770
WP_088886109_1  ---TP-------------------------P---ELALSLECPN--E-PVLL----------G----E------TVICNIIVKKNVLPSD-VPY------ 593
WP_088882563_1  ---------------------------------------------------------------------------------------------------- 709
WP_015859709_1  ---GEFED----------FLTVNVTFD---G---PVLAWMEC--EDAVPLY----------------------DEIDCYVHILNNA-LED-IPV------ 512
WP_014122675_1  ---NKNRD----------VLTVNTTPG---G---PVWAWMECNSQDNGIVYV----------G----E---TGNHLYCTVYVKNDG-PVD-VPV------ 473
WP_014122677_1  ---GHK-------VAASKLTVIDDNP---------VRAVMKCDP--A-VVPL----------G----R------STRCTVHFELGN-ADT-VTL------ 485
WP_056934707_1  ------------------------------S---YFTESIPSNA--Q-EVTL----------S----KTIKVTYTKWCNGINSAPGGEEV-LSA------ 535
WP_068320152_1  ---NYDGKPNGKGEEHWSEVRVEVKP----W---QASLKLECDG--K-VILG----------K----D---DVDDLDCTLYLFRTNPSDS-VQV------ 403
WP_082781392_1  ---EVNSNS---------------------Q---LIMSKASCED--I-KFDF----------S----D-YAYRATVSCEVYLHNLR--NQ-EVYIEHISV 400
WP_010884703_1  ---------------------------------------------------------------------------------------------------- 534
WP_062388971_1  ---------------------------------------------------------------------------------------------------- 494
WP_074631387_1  ---NYDGKPNGKGEEHWSEVGVEVKG----S---VVSPNLDCTR--E-VFPG----------E----K-------VTCIVSWNPKTTLSS-LTL------ 279
WP_010884704_1  ---NYDGEANGKGEEHWSKATIEVKPV---G---DVVASMSCYP--T-TVPL----------D----S------STTCTVSFELKS-PAT-VTL------ 288
WP_056934708_1  ---GQEED----------EKVITVNSQ---G---SISAVMTCSP--E-FVTE----------D----V-----NSVSCRVDVENLG-ETT-EDI------ 213
WP_048151448_1  ---NYDGEPNGKGEEHWAEVTMEVKPV---NGRELKQVGFECDD--P-EFNW----------N--GIE---YKAPIVCRASVYNPS---K---------- 219
WP_088863691_1  ---NYDGEPNGAGEEHWSNVAVEVKPM---KGSELKQVGFDCND--P-EFNW----------K--GTE---YKASLVCRAFIYNPT---Q---------- 206
WP_048810998_1  ---NTDGKPNGNGDEKWKKVKVEVKPI---KGIGLKQVGVECDN--L-YFKY----------D--MGN---YKAKLACKFVIYNGY---N-FSL------ 134
WP_048148049_1  ---------------------------------------------------------------------------------------------------- 311
WP_055428947_1  ---NIKQN----------------------G---PVNASMECKP--A-IVPL----------D----G------STTCTVYFELKS-ADT-VTL------ 162
WP_099209381_1  -------TASIPGEEEKSTVTVGVEPT---NGTKLEQVGFECDN--P-EFNW----------N--GIE---YKATVVCRAFIYNPT---Q---------- 165
WP_014122994_1  ---NTDGKPNGNGEEKWRKVKVEVKPI---S---SVYATLDCPQ--S-VPQN----------D----E-----RPTQCKIKVWNTG-NKV-FST------ 128
WP_048165381_1  ---NYDGKPNGAGEEHWSEVRVEVKSV---G---NVIASMECEP--V-IVPL----------D----E------STTCTVHLELKS-ADT-VRR------ 128
WP_048165382_1* ---------------------------------------------------------------------------------------------------- 102
WP_068319940_1  ---NYDGQPNGKGEEHWSEVRVEVEPP---S---VQTAFMDCSN--L-VLSA----------E--RGN---LILDMSCEVYFTNPT---D-VEW------ 82
WP_068320169_1  ---GEWED----------EETITTTSK---A---SIEAWMECAP--E-FVSD----------D----V-----NSVTCEINVKNSG-SAS-VEV------ 199
WP_068320161_1  ------------------------------W---MVALLYSGLA--F-AISK----------K----H--------------------SL-LIV------ 81
WP_048055992_1  ---NYDGEPNGKGEEHWSEVRVEVKPM---NGTELKQISFECSD--L-YFDF----------R--DYA---YRATLSCRVYLYNPA---S-IKA------ 77
WP_074631389_1  ----------------------------------LKHVTSECDP--I-HFKY----------N--MGN---YEATLTCRLTIYNGY---S-YDL------ 44
WP_014012910_1  ---------------------MEVKS----W---KADLKLECDD--E-VVLG----------E----N---DVDDLDCTLYLFRTNPEES-VQV------ 45
WP_015857844_1  ------------------------------D---KVMASMHCIP--T-SIEP----------G----G------AVICDIDFKLLNPNET-ATL------ 61
WP_099209388_1  ------------------------------G---SVIANMTCMD--S-AIPY----------D----G------STTCKITLLNTG-KRS-ISV------ 44
WP_088865600_1  ---------------------------------------MTCSP--E-FVSE----------D----V-----DSVSCRIEVENLD-SMP-VDI------ 48
WP_048165388_1  ---------------------------------------MNCPE--Q-PILL----------G----E------AGVCEVVVRKNT-LSTAVGY------ 31
WP_048055998_1  ------------------------------S---SIIASMECKP--S-VVAL----------D----G------STTCTVHLELRQ-PDT-VTL------ 42
WP_088863688_1  ---------------------------------------MVCDP--A-IVPL----------D----S------STVCTVHLGLRS-ANT-VTL------ 30
WP_048151737_1  ------------------------------S---NTIASMECKP--T-VIPW----------D----G------STTCTAHFDLKS-TDT-VTL------ 40
WP_048055995_1  -----------------------MKPM---DGTGLKQVGFECDD--V-YFDF----------K--NGE---YIAPITCKAILYNPY---S---------- 43
WP_055430151_1  ---------------------------------------------------------------------------------------------------- 33
WP_048150566_1  ---------------------------------------MDCPL--E-VFPG----------D----K-------ITCVASWNPKTTLSS---L------ 28
WP_099209386_1  -----------------------------------------CLS--L-CFPF----------G------------------------------------- 26
WP_088886110_1  ---------------------------------------------------------------------------------------------------- 196
WP_068320165_1  --------------------------M---NGTELKQVGFECND--P-EFHW----------D--NIE---YKAMLVCKAFIYNPA---Q---------- 40
WP_055428943_1  --------------------------M---NGKELKQVAFECSD--L-YFTKIVTGVNLESAKNILLN---YQAEVKCIATIYNPT---D-KKI------ 55
WP_014122673_1  -----------------------------------------------------------------------------AVMQIDNFG-D------------ 14
WP_088856137_1  ---------------------------------------------------------------------------------------------------- 177
WP_048152596_1  ----------------------------------------------------------------------------------MHLTKNES-VNL------ 11
WP_048151739_1  --------------------------------------KMDSKT--V-AADY----------I----L------SGTAAI---VDP-NTA-ISF------ 125
WP_088865604_1  ---NVRQE----------------------G---PVKASMECKP--T-VIPL----------D----G------STTCTVHFELKE-PTT-ITV------ 131
WP_088865603_1  ---------------------------------------------------------------------------------------------------- 0
WP_048150577_1  ---------------------------------------------------------------------------------------------------- 0
WP_055430149_1  ---------------------------------------------------------------------------------------------------- 0
WP_068320157_1  ------------------------------S---NVIASMECMD--S-AIPY----------D----G------STTCKVTLVNKE-KTS-VTI------ 42
WP_014788172_1* ---------------------------------------------------------------------------------------------------- 0
WP_048165379_1  ---NYDGEPNGAGEEHWSEVKVEVKAM---NGTELKQVSFECDD--P-EFNW----------N--GIE---YKATLVCKAFVYNPA---Q---------- 75
WP_068320171_1  ---------------------------------------------------------------------------------------------------- 0
WP_088865607_1* ---------------------------------------------------------------------------------------------------- 0
WP_058939190_1* ---------------------------------------------------------------------------------------------------- 0
WP_068319946_1  -------------------MRVEVKPV---S---NVVASMECEP--A-VVPL----------D----E------STTCTVHLELKR-SDT-VTL------ 44
WP_048150353_1  ---------------------------------------------------------------------------------------------------- 0
WP_048150344_1* ------------------------------W---IVALLYSSLA--L-IILK----------R----H--------------------SQ-HNG------ 79
WP_048055999_1  ---------------------MDVKPP---S---IQTAFMECSD--L-ALSA----------K--RGD---LVLDMSCEVYFTNPT---D-IEW------ 45

WP_058939188_1  ------NFEASNYYT---------------QGSY-VSPIECTLKFRNAGDTQVYVK---E--L----S---AD-ISVSPPDLEKTIPDHIAVSVNE---- 1007
WP_014788798_1  ------TLKEVWFGT---------------KKVW------------DVDLSSVKMV---K--G----P--------------TLTPTSTIQVEIRL---- 960
WP_048151755_1  ------NFKASRLYT---------------QGSY-VSSLECTLKFRNVNDVPVHIT---E--L----S---TE-ISVSPPDLEEAVPDHTTVSVNE---- 957
WP_088181398_1  ------NLEEVDFGG---------------KKVW------------PNGPSSVTVN---K--R----T---VT-LT------PTNMQEDLTITINI---- 959
WP_048165390_1  ------KISGIDVGG---------------AKVW------------PSDFKEVTLS---K--D----T---LK-VT------PNDPDDETHIKITI---- 949
WP_088865610_1  ------NFEASNYYT---------------QGNY-VSPLECTLKFRNIESARIYVK---E--L----S---TE-ISVSPPNLEEVIQDHIAVPVNE---- 945
WP_014012907_1  -------QENVFLNAVS-------------VSEW-HTD--------NSDFTRSLGS---S--W----T---VE-Y-------PTVIQSSETATIIF---- 968
WP_099209391_1  ------KVTRVDMDAYI-------------LKNH-YTKEIT-----PEFTEEVLTK---E--V----P---SK-R-------TGVIRLEFHDTLST---- 925
WP_088863686_1  ------EVTDVYFAG---------------GQIY------------DSGKSDNFIV---P--K----P---RS-LDL-----GPNSLGVFAFTIPI---- 914
WP_068319935_1  NSYSLGDLENMPAGSLN-------------TGEW------------SINPSSFTIP---A--Q----T---TK-IV------HFNLPLEISPWIPI---- 903
WP_011013125_1  ------ELDESRVLSID-------------F---------------PMDHSNGVIT---L--D----T---TT---------TIKAGETGNVYITF---- 885
WP_042692137_1  -------TARIRMDG-------------------------------ELIDLSRTLG---N--T----N---PT-LN------PGRKEFRIGVNINN---- 833
WP_052696221_1  ----------------------------------------------AITTKMLDSG---E--D----T---SR-AI------STNIITDV-IPAKF---- 775
WP_014122995_1  ------SVASVDFGTL--------------REVW-N----------GANTKGINVD---L--A----S---VV-L-------PPNDDKTIVVHIDL---- 780
WP_088886144_1  ------QVTKVMFGG---------------YEVW-N----------GENWKAVMVN---Y--I----T---GT-TI------KRHSEGIIYIGIDV---- 738
```

```
WP_088856136_1   ------NLEEVDFGG---------------RKVW------------PTGPSSVRVN---K--Q----T---MT-LT------PTTMQGDLTITINI----  714
WP_015857921_1   -------------PTT--------------KKAF-M----------GCDNMKLSAE---K--G----N---LV-L-------DMKCEVYFTNPTDV----  784
WP_014733996_1   ----------------------------------------------------------------------------------------------------  770
WP_088886109_1   ----------------------------------------------AITAKTLDDD---K--D----T---SR-II------STNIIKDI-IPARF----  623
WP_088882563_1   -------------------------------KLW------------DSKIGTISVS---R----------------------------------------  723
WP_015859709_1   ------QVTKVTFSG---------------YEVW-D----------GENWEAVKVN---Y--V----T---GT-SV------KGNSEGIVGIDIDV----  557
WP_014122675_1   ------RLTKVMLGG---------------YEIWGE----------DRHYDAVDID---Y--VDGGLT---GM-TV------KRQSMGHISVHVDL----  523
WP_014122677_1   ------NLKEIDFGG---------------RRVW------------PDGPSSVGVE---A--Q----R---IT-LS------PTNMRETFTITLTI----  529
WP_056934707_1   ----------------------------------------------PAGCGETAEE---T--E----T---VT-KTIE----LFDVDVSVSDTTVK----  568
WP_068320152_1   ------KISEIYVGG---------------AKVW------------PSNFEGIIVS---K--E----V---LK-VT------PYDPDDETHIELTI----  447
WP_082781392_1   EDYSLGQLEKIPLGSLG-------------VEKW------------LISPQEFTIK---P--W----E---TR-RI------VFSLPIYVSSYIPI----  452
WP_010884703_1   ----------------------------------------------PQGRSST-----------------------------PYPKKFHGNVSVDP----  555
WP_062388971_1   ----------------------------------------------PPYPR-------------------------------------------------  499
WP_074631387_1   --------EEVWFGT---------------KKVW------------DIGLSSVELV---K--G----P--------------TLTPSGTIQLEIKL----  317
WP_010884704_1   ------NLEEVDFGG---------------KRVW------------PEGPSSVIVK---E--K----T---IT-LT------PTNMQDDLTITITI----  332
WP_056934708_1   ------WITNIYFAG---------------GEIY------------DKSSADNLVI---PSPG----S---LT-L-------NPYSTGTFTFTIPI----  258
WP_048151448_1   ---------------------NNVEISIVNVKTW------------TIDNQALENS---L--G----HTWEVE--Y------PNVIHGSETATII-----  264
WP_088863691_1   ---------------------NDISLNAVAVKEW------------HTDNTDFTNS---L--G----PWVVVD--Y------PDTIRSSETSTIT-----  251
WP_048810998_1   ------EVNGKIMAKMNASVLN----------KY------------LQ-------------------SDSLVTSIN------PHTISAGQYFAINSTAYF  181
WP_048148049_1   -----------------------------------------------YAPMEFSIV---R--E----P---LR-NR------------------------  327
WP_055428947_1   ------NLEEVDFGG---------------KKVW------------PNGPSSVTVN---K--Q----T---VI-LT------PTNMQEDLTITLDI----  206
WP_099209381_1   ---------------------KTIYLNSVSVKEW------------RTDNSDFTQS---L--S----SSWVVE--Y------PTVIKSSETISIT-----  210
WP_014122994_1   ------RITKVVFGN---------------NVIW-N----------GTDTVAATID---Q--V----S---IT-V-------PPHGVNNVSLTFTV----  172
WP_048165381_1   ------NLVEVEFGR---------------KRVW------------PDGPSSVTVN---K--Q----T---IT-LT------PTNMQEDLTITIDI----  172
WP_048165382_1*  ----------------------------------------------------------------------------------------------------  102
WP_068319940_1   ------RITDVVT-E--ARILK----------------------------GQCTQE---I--T----PEFSDTL-L------TPTVPAKGTGRLQVNF--  127
WP_068320169_1   ------WVTNIYFAG---------------GEVY------------DRSSDNNLVN---P--N----P---SS-LSL-----NPALQEHSRSQFQL----  244
WP_068320161_1   ------KLNLREFGG---------------KKVW------------PNGPSSVTVN---K--Q----T---VT-LT------PTNMQEDLTVTITV----  125
WP_048055992_1   ------KVKDIAVLEYSLGELEDVPAGSLGTGKW------------SINPSSFTLS---P--Q----ETKIIT--F------NLPLEISSQIPIS-----  137
WP_074631389_1   ------KVTGNIVSDINVSILN----------KY------------LQLPSGS--------------SSPVVSYID------PKTIPAGGYATITSTAHF  96
WP_014012910_1   ------RISGIDVGG---------------AKVW------------PSEFKGLTLS---K--D----T---LR-VT------PNDPDDEIHIKITI----  89
WP_015857844_1   ------KLKKVYLED---------------KEIW------------PRGPSRGTVI---V--P----N---SK-FHLG----PHDIEGSIAITLTF----  107
WP_099209388_1   ------SVSSVDFGT-V-------------HNAW-A----------KSYQRGIEVK---P--I----K---IT-L-------PPHDDKEIDVKIDL----  89
WP_088865600_1   ------WITDVYFAG---------------GKIY------------DRTEGDNLIA---PNPE----S---LM-L-------NPDYSEIFTFTIPI----  93
WP_048165388_1   ------SVVTKLLDD-------------------------------DMDTSSV---------I----S---VT------------MVKDV-IPSQF----  61
WP_048055998_1   ------SLQEVDFGG---------------KKVW------------PDGPSSVTVN---K--Q----T---VT-LS------PTNMREDLTVTITI----  86
WP_088863688_1   ------NLKEVDFGG---------------KKVW------------PDGPSSVRVN---R--Q----T---VT-LT------PTNMQEDLTIIITI----  74
WP_048151737_1   ------NLEEVDFGG---------------KKVW------------PNGPKSVTVN---K--Q----T---VT-LT------PTNMQEDLTITIDI----  84
WP_048055995_1   ---------------------EDASITKITMNA-------------RVDSQDLERA---L--S----VPWSAS--A------PEAIPAGGYGTVI-----  87
WP_055430151_1   -------LEEIYFAG---------------IPVW-T----------GVNDDSVKIPITSK--R----T---VT-VL------ANSENDDMGFEIIF----  80
WP_048150566_1   ------TLKEVWFGT---------------KKVW------------DVDLSSVNLV---K--G----P--------------TLTPSRTVKLEFRL----  68
WP_099209386_1   ------------FWG---------------KKVW------------PNGPSSVTVS---G--Q----T---VT-LS------PTNMQDDLTITITI----  64
WP_088886110_1   ----------------------------------------------------------------------------------------------------  196
WP_068320165_1   ---------------------EAVSLNSVSVKEW------------HTDNSEFMSS---L--D----SSWTVE--Y------PEVIQSSKIATIV-----  85
WP_055428943_1   ------TIEELIIGQP----IATPTALDVGLKE-------------------FNGE---I--S----S-NVVT--------------PHNYVIIK-----  97
WP_014122673_1   ------------FAG---------------GKVY------------DSSEGGNLVV---PAPD----V---LS-L-------NPGSSGTFTFTIPI----  53
WP_088856137_1   ----------------------------------------------------------------------------------------------------  177
WP_048152596_1   ------KLEGIYLNG---------------KLVW-SQ---------NISSEAVRVI---N--P----Q---VS-LG------PTNVEGSVTIVITL----  57
WP_048151739_1   ------QLGSIRISG---------------IRL--------------SSKLSFDP---------------------------------------------  145
WP_088865604_1   ------NLKAVKFGG---------------KKVW------------PVDENGEDP---------------------------------------------  153
WP_088865603_1   -------------------------------------------------MGTVNIP---K--P----K---AT-LD------LTNMREDLTITITL----  28
WP_048150577_1   -----------------------------------------------MSHSRAVIS---D--K----S---TT-TF------PAHRSGYLHVKFS-----  29
WP_055430149_1   ----------------------------------------------------------------------------------------------------  0
WP_068320157_1   ------SVTSVDFGT-V-------------QNAW-A----------RSYQWGIKVD---P--I----T---IT-L-------PYNGDKEIDVKIDL----  87
WP_014788172_1*  ----------------------------------------------------------------------------------------------------  0
WP_048165379_1   ---------------------ENVLLNSLSVNEW------------HTDNPDFTRS---L--A----PRGRLS--I------PA----------------  109
WP_068320171_1   ----------------------------------------------------------------------------------------------------  0
WP_088865607_1*  ----------------------------------------------------------------------------------------------------  0
WP_058939190_1*  ----------------------------------------------------------------------------------------------------  0
WP_068319946_1   ------SLQEVDFGG---------------KKVW------------PNGPSSVTVN---K--H----Y---QN------------PVGILVLKIIS----  83
WP_048150353_1   ----------------------------------------------------------------------------------------------------  0
WP_048150344_1*  ------N---------------------------------------------------------------------------------------------  80
WP_048055999_1   ------HITDVIT-E--ARILI----------------------------------------Y----PRDYT--------------------RV------  66

WP_058939188_1   ----NVNPGE-------AI-------P---------VQIQSKAT-TTD-RQML-LRVDGTM------------ATLYI-GYMI-------EGIINGANKI  1057
WP_014788798_1   ----DDDFAN-------YYFG---ERP---------WDPVDRVF-SSY-KDRL-AGYTYKV------------KFIFE-NNVI-------AEDLVMVK--  1012
WP_048151755_1   ----DVKPGE-------FI-------T---------IQIQDRTV-TTD-RQML-LRVDGTT------------ATLYL-DYTV-------EGMINGVSKI  1007
WP_088181398_1   ----DDELAD-------YYFG---K----------------PIY-RTY-IDKF-AGHSYLI------------KAEFS-EF-------------------  994
WP_048165390_1   ----DENFAE-------FYFG---IDP---------L----FPW-HSY-VDKL-ATEQPWE------------IVIHF-E--------------------  986
WP_088865610_1   ----NVNPGE-------II-------P---------IQIRGKAL-TTD-RQML-LRVDRTT------------ATLYL-DYTI-------DGIINGVSKI  995
WP_014012907_1   ----KNTAHT-------GLIT---LER--------------DLF-GDY-ISIS-LKYVVSP-------Q--NGNDIIF-TGI------------------  1009
WP_099209391_1   ----SGVLGK-------GIKL---LEL---------DGVTVPVQ-ANF-TILV-DGKPTVI-------D--SHGREWY-L--------------------  969
WP_088863686_1   ----NDELQK-------RI-------P---------LDLQDINL-GTP-VAIK-ARLHPPG-------K--PVMCVVQ-MNPR-------PVT-------  960
WP_068319935_1   ----SVADAT-------EVV---------------------LKD-GEF-ITVE-IPYTLGY-------S--YDENTWT-H--------------------  938
WP_011013125_1   ----KAWTSD-------FTAF---ARY---------W------L-RGY-YHLP-VTIEFEL-------K--MSRGNNF-EYS------------------  925
WP_042692137_1   ----DLAQNE-------FNYG---------------------SF-LDF-LDSE-IVTKENC-------G--HRVSKCQ-VFKW-------LPTPHAFSIE  881
WP_052696221_1   ----SGKIAE-------VFVP---LDE---------RMTEALKL-SDV-RDLT-PRTGVWS-------C--EDGTCYY-TLKR-------ADTPHYLKVT  832
WP_014122995_1   ----EG-LAQ-------TIWG---DKY--------------------Y-ARNF-LEHESYM------------ITVNF-DN-------------------  811
WP_088886144_1   ----DGWLSE-------RVFG---DDY---------YIYLFLDG-GQP-KEHI-INVDLRL-------N--DTRRVL-----------------------  780
WP_088856136_1   ----NDELAD-------YYFG---H------------------W-G-Y-THKLLETHSYRI------------E-AYL-EGL------------------  747
WP_015857921_1   ----E--------------WN---------------------------------------I------------TRIDI-D--------------------  794
WP_014733996_1   -----------------------------------------------F-CER------------------------------------------------  774
WP_088886109_1   ----TGKIAE-------VFVP---LDE---------RMAEALGL-NDV-RDLT-PRINTWV-------C--ENGYCHY-TLER-------VDTPHYLKLK  680
WP_088882563_1   -------------------------------------------Y-STH-SFRL-L---------------------------------------------  732
WP_015859709_1   ----DGWLAE-------RVYG---DDY---------YIYLFLDG-GQP-MAHM-ITVDLQV------------ANKHH-TL-------------------  599
WP_014122675_1   ----SK-LSE-------EIYG---RVD---------YKYLFYDDEGKP-LEYM-VSVGLQL------------GNKTQ-NV-------------------  565
WP_014122677_1   ----NDTLAN-------YYFD---H------------------W-G-Y-THKLLENPSYLV------------E-VHL-TNL------------------  562
WP_056934707_1   ----VNEPVG-------FTF----------------------TI-TNY-MTSS-KTFNLLV---------------------------------------  593
WP_068320152_1   ----DENFAE-------FYFG---IDP---------L----FPW-HSY-VDKL-ATDRPWE------------IVLHF-E--------------------  484
WP_082781392_1   ----SGSDAT-------EVV---------------------LKN-GEY-TNIK-FEYTLGY-------S--IDRKVWH-H--------------------  487
WP_010884703_1   ----RRR---------------------------------------------------------------------------------------------  558
WP_062388971_1   ----------------------------------------------------------------------------------------------------  499
WP_074631387_1   ----DDNFAN-------YYFG---ERP---------WDPIDRVF-SSY-KDRL-AGYTYKV------------KFIFD-DNVT-------AEDLIMVK--  369
WP_010884704_1   ----NDELAN-------YYFG---ERP---------W----LST-GSY-KDRL-VGYSYLV------------KAEFN----------------------  368
WP_056934708_1   ----NDELQE-------RV-------P---------VDLSDINP-GTP-VAVK-AYLNTLQ-------E--PIMFVIQ-MNPR-----------------  301
```

```
WP_048151448_1  --------------------F---KNTART----GLLALEKDLF-GSY-ISVS-LRYIISP-------Q--NGDD--I-AF----------------T-- 304
WP_088863691_1  --------------------F---RNTAHT----SLHTLEMDLF-GVY-ASIS-LTYIISP-------Q--HGDD--V-HF----------------T-- 291
WP_048810998_1  EISKSSLPRLIRVLSGAGLIFPYWEDAAN------DIRVETEFY-GAT-IPIE-ITYDVPF-------D--RIG--IQ-RF----------------T-- 242
WP_048148049_1  ---------------------------------------------------------------------------------------------------- 327
WP_055428947_1  ----NKEFVN-------YYLD--------------------YPL-LNY-GYKM-TKYSYLI------------VAKIS-DGLR----------------- 242
WP_099209381_1  --------------------F---KNTAHT----GLTTLEKDLF-GAY-VTMY-LKYIISP-------Q--DGND--I-IF----------------T-- 250
WP_014122994_1  ----NG-IAT-------LLFG---D--------------------PFF-NYKL-PEYSPYL------------VKVYF-T-------------------- 202
WP_048165381_1  ----NDELAN-------YYFG---K----------------PVY-RTY-IDKF-AGHSYLI------------KTKFS-EL------------------- 207
WP_048165382_1* ---------------------------------------------------------------------------------------------------- 102
WP_068319940_1  ---HDTLPAS-------GFLG---KS-----------VSIGDLA-GVS-VPVE-VDLTLEVNGKTYVVD--SQGNTWSLKY----------------K-- 180
WP_068320169_1  ----MMN-------------------------------------------------------------C--RRGFRWI---------------------- 255
WP_068320161_1  ----NDELAD-------YYFG---H------------------W-GYT-HRLL-ETPSYLI------------EVHLA-GL------------------- 158
WP_048055992_1  --------------------G---ADATEV----VLQ------Y-GRY-VSVE-FNYTLGY-------A--YDEDTWI-HF----------------S-- 173
WP_074631389_1  KISKPDIFLI-------GDIFPYWEDARN------DIQVE-GIY-GAR-VPVK-IIHDVPF-------D--KLG--IR-RF----------------T-- 149
WP_014012910_1  ----DENFAE-------FYFG---IDP---------R----FPW-HSY-VDKL-AMEQPWE------------IVIHF-E-------------------- 126
WP_015857844_1  ----NNEMAD-------CYFG---K----------------PVL-DDY-RDKF-GGRTYKI-------T--AVV-------------------------- 139
WP_099209388_1  ----RKLAQD-------FW-----------------------GN-DHF-AYKF-AEHKSYM------------ITVHF-D-------------------- 119
WP_088865600_1  ----NGELQK-------RV-------P---------VDLSDIDL-GTP-IAIK-AYLNRLK-------E--PVTYVVK-MNPR----------------- 136
WP_048165388_1  ----TGKIAE-------VYVP---LDE---------KMAKALEL-NDV-RDLT-PKTNTWV-------C--ENGDCHY-TLER-------VDT----P-- 112
WP_048055998_1  ----NKELAN-------YYFG---K----------------PFY-RTY-IDEF-ADHSYLI------------EAKFS-EI------------------- 121
WP_088863688_1  ----NDELAN-------YYFE---KKP--------------LGW-GSY-SDRL-VGYSYLI------------KAVFS-GGVA-------SSDIVEIR-- 121
WP_048151737_1  ----DDSLAN-------YYFG---K----------------PIY-KTY-IDEF-AGHSYLI------------EAKFS-EL------------------- 119
WP_048055995_1  --------------------F---STTAKVYGTLDGINLIKDLW-GLS-TTII-ILYDVHW-------E--GTSEEIS-SF----------------E-- 133
WP_055430151_1  ----NDNLAE-------KLFT---ADY---------F--LS-PG-GSY-YKYL-LTPGGQP-------AIYRVAVKLS-T-------------------- 123
WP_048150566_1  ----DDDFAN-------YYFE---ERP---------WDPVDRFL-SSY-KDRF-AGYTYKV------------RFIFD-NNVT-------SETLVMVK-- 120
WP_099209386_1  ----NDALAN-------YYFG---ERP---------W----LSD-GSY-KDRF-VGYAYYI------------KAVFS-NNIV-------AGDIVEIT-- 112
WP_088886110_1  ---------------------------------------------------------------------------------------------------- 196
WP_068320165_1  --------------------F---KNTAHT----GLITLEKDLF-GAH-VLIS-LRYVISP-------Q--NGND--I-IF----------------T-- 125
WP_055428943_1  --------------------F---TSSAEV----PLTVLELGLW-GSV-YSLQ-LPYTLKT-------S--QGT---F-EF----------------R-- 136
WP_014122673_1  ----KDELQK-------KV-------P---------VDLSDMNF-GRP-VAIK-VYINQKR-------E--PVMHVIE-MNPM---------D------- 97
WP_088856137_1  ---------------------------------------------------------------------------------------------------- 177
WP_048152596_1  ----DNELAD-------SYSG---K----------------PVF-DSY-LNKL-AGRTFTI------------TATID-G-------------------- 91
WP_048151739_1  --------LA-------FL------------------------W-NAFIKPGI-LEHT------------------------------------------ 162
WP_088865604_1  ------------------L------------------------W-EL----------------------------------------------------- 157
WP_088865603_1  ----NRQFAN-------YYLD---YPP---------L---------DY-SYKL-TGYSYMV------------IVDFA-EIPQVSDVVYLTGS----A-- 75
WP_048150577_1  -----ASISD-------FRAL---ANY---------W-----VY-G-Y-YDLP-VTMRLHL--------------VEF-MNNA-------PKS----Y-- 68
WP_055430149_1  ------------------------------------MDLSDINP-GTP-IAIK-AYLNQLK-------E--PITCVVE-MNPR-------PVT------- 37
WP_068320157_1  ----RMLAQD-------FW-----------------------GS-SHY-VYKF-AEYTSYG--------------------------------------- 111
WP_014788172_1* ---------------------------------------------------------------------------------------------------- 0
WP_048165379_1  ---------------------------------------------------------------------------------------------------- 109
WP_068320171_1  ------------------------------------MDLSNINP-GTP-IAIK-AYINQLK-------E--PVMSVVE-MNPI---------D------- 35
WP_088865607_1* ---------------------------------------------------------------------------------------------------- 0
WP_058939190_1* ---------------------------------------------------ML-LRADGTT------------ATLYI-DYTI-------EGMINGANKI 28
WP_068319946_1  ---------------------------------------------------------------------------------------------------- 83
WP_048150353_1  -------------------------------------------------------------------------------MDPM----------------- 4
WP_048150344_1* ---------------------------------------------------------------------------------------------------- 80
WP_048055999_1  ---H------------------------------------------------------------------------------------------------ 67

WP_058939188_1  V-----G------------KGVTEITIGINVDT-RTAWADYGYD-TAT-------IFISFVKITKAT----K------IAK---IMKTAGKLTG-----V 1113
WP_014788798_1  ------D------------RGIIDEVKNFYNDI-KDDIDAGKYV-AAT----------YIFLKGSGK----L------ATK---TAKRVGGLLS-----I 1064
WP_048151755_1  V-----V------------KGIAESTLRLSVNQ-GIVYADVTAD-LLL-------LIEGPKVSSKLT----K------WFP---VIDKIPGGDA-----I 1063
WP_088181398_1  ------N------------YSVSDVIKIIYKDT-RSGTEKAIDY-GST----------TGEIISTAQ----L------VVE---GSNPVGWIAF-----A 1046
WP_048165390_1  ------N------------APPISTTFQILKNS-GTTIEDAVEY-GGSGTFVVTEILEKAGYITTEA----T------GSG---QLAVFGSRLF-----G 1048
WP_088865610_1  V-----A------------KGVTQSTIGINVDT-KTVWADYGYD-TVT-------IFISFVKITKAT----K------IAK---ITKTAGKLGG-----V 1051
WP_014012907_1  ------------------------DTINIGQDN-KDVIVDVGTN-VIL----------VGADVKAGI----T------ILK---LARNGEVVSA-----F 1055
WP_099209391_1  ------D------------YTAYADVTIVVDGG-KVTEDIATDL-VAN----------VGVDIAVGA----T------IGT---LILPGAGTVA-----G 1021
WP_088863686_1  ------A------------GDVARDAYHAVKII-KDEVIGTFTD-GAT---------AGGYLLRYVG----V------PPE---VGGPIGGAMA-----L 1013
WP_068319935_1  ------F------------SGEIYDTIQVKMDT-KTVATDYVLS-GVM-----------------AV----I------DPN---TAISFSVGSF-----K 983
WP_011013125_1  ------K------------YEKYKISTQIQVKT-SDTIYLAVSA-DIG----------SFFTGGEGL----R------GIV---VAFKEGKRAK-----T 977
WP_042692137_1  IYNDNQL------------LDSTETTVTLTYGN-YYEKKFMEYY-VAP--------LASGATAGAAT----A------LAE---VSVSAGISAA-----K 941
WP_052696221_1  VRLGSDQ------------YRNLEGIIMARYTG-SKYWNEDADE-GIK--------------KGALM----G------IAP---VGLAYAPVEY-----L 886
WP_014122995_1  ------I------------Y-PVSDTVSVFYEESSIEKHSREL---GS--------IIGGFIGGVIAGAITEGSATEEGIV---TGAALGAILGDKISSG 878
WP_088886144_1  ------G------------YTVTVYSGDDKEGG-KIETADYILK-SAQ--------VAGGASTVLGF----V------LAI---GGNPTGLIIA-----G 834
WP_088856136_1  ------S------------Y-PVSDVITITGKE--EDWKDKATS-LGA-------DFVGGSIVAVAA----G------S-------NPISL-TF-----V 795
WP_015857921_1  --------------------------------------PSRIL-----------------------------ATERTKDVT---IGIPAETIITRKVAPG 824
WP_014733996_1  ---------------------------------------------------------------------------------------------------- 774
WP_088886109_1  IRLNSGE------------YMSLEGVITARYTG-SKYWNEDANK-G----------------IGTAS----T------MIT---LPIAVSWKPA-----W 732
WP_088882563_1  ---------------------------------------------------------------------------------------------------- 732
WP_015859709_1  ------G------------YVTMIYPGDDKEGA-KKELPDYIIT-GAE--------LGGAFLTVAGF----V------I-G---SGEIISTGGV-----L 652
WP_014122675_1  ------G------------QVVTMIP----VGI-KKDSLDYLSI-GLS--------VIGGIITVISF----V------L-V---LSFGLEAIGV-----I 614
WP_014122677_1  ------S------------Y-PVSDVITLTEKK--EDWLDRLPS-LFA-------DFIGGAIVAFAT----A------S-------NPLSL-PF-----V 610
WP_056934707_1  ----TAG------------GKSKEDTLTVPTGE-TKNQDSAVNF-------------------------------------------------------- 620
WP_068320152_1  ------N------------APPISATFRILKNS-GISPEDVVEY-GGSGTFIATEILERAGYITTEA----T------GLG---KVAVVGSKLF-----G 546
WP_082781392_1  ------Y------------FNSVSQHVKITMDT-KTVVADYVLS-GIA-----------------AI----T------DPN---TAISISIGSV-----R 532
WP_010884703_1  ---------------------------------------------------------------------------------------------------- 558
WP_062388971_1  ---------------------------------------------------------------------------------------------------- 499
WP_074631387_1  ------D------------RGIMDEVKNFYNDF-KDNIDAGNYA-AAM----------YIFLKGGEK----L------GKQ---GAKRAGGLLT-----A 421
WP_010884704_1  ------E------------SITITDAITVSSGD-SSTIEDLETA-VKV----------TGVGVSALE----E------VAS---HAGQLEIVAS-----G 420
WP_056934708_1  -------------------------EKGVLKEV-KDYIVNKVEN-DPW-----------GTSNKVAV----I------IIT---GKISVEINPA------ 344
WP_048151448_1  ------G------------T----DTINIRQDN-KDVIVDVGTN-LVL-------IGADVKAGITIA----K------LAKNGEVIKALKNAWP-----Y 358
WP_088863691_1  ------G------------Y----DTINIRQDD-KDVVVDGGTQ-AIL----------DFIGGEELV----V------AVRTKDALKTL----P-----F 338
WP_048810998_1  ------F------------I----ESTYIEVDQ-IAVVTDVATD-V----------FLTYELKTKTI----P------LADIEFIQNALNKLPP-----S 293
WP_048148049_1  ---------------------------------------------------------------------------------------------------- 327
WP_055428947_1  ------V------------SDVVQLSDPVNVQT-FEDKARKAYG-IAKTIKSAYDDAKSGIIGILET----V------TVK---GGKELAWILH-----Y 304
WP_099209381_1  ------G------------T----GLIHIRQDN-SDVAIDVGTN-LVL-------IGGEVKVIVISV----K------LIRMGEMIKGLSELAP-----V 304
WP_014122994_1  ------N------------LPPASDIVEITEAN-SIVQTIYIYG-TAAISGATIGMAVAYFGGGGIL----A------GAL---SGGASGVAGQ-----A 264
WP_048165381_1  ------N------------YPVSDVITIIYKDT-RSETEKAIDY-GST----------AGEIISTAQ----L------VVE---GSNPVGWIAF-----A 259
WP_048165382_1* ---------------------------------------------------------------------------------------------------- 102
WP_068319940_1  ------A------------I----KMVKIKTDT-DNVTRDLVGE-LTL-------GLAGAVAGT--I----A------GAK---IGAALGSVVP-----G 229
WP_068320169_1  ---------------------------------------------------------------------------------------------------- 255
WP_068320161_1  ------T------------FPISDVITIVEDE---KDWKDVAVG-LAADFAGGAVVVVIAGTNPISL----S------FVA---TFLAGAATTE-----Y 218
WP_048055992_1  ------GSV----------Y----GNVLVKMDT-KTIAADYMLT-VAM-------AYLSAWTLIKAP----G------KVKIGKVEFNPAGILI-----G 229
WP_074631389_1  ------L------------Q----SSAYVEVDH-IAVAADIAAD-T----------YLTYKLGSKVQ----P------IANIKFIEDALNKLPP-----G 200
WP_014012910_1  ------N------------APPISTTFQILKNS-GTTIEDAVEY-GGSGTFVVTEILEKAGYITTEA----T------GPG---SLAVVGSKLF-----G 188
WP_015857844_1  ------D------------GISTPVSAQIKIKN-TGLWDSFIWV-LEY-------LIVLIYLISIAV----S------LST---KNFPIGSLLL-----V 194
```

```
WP_099209388_1  ------N------------GVVVSDILTIKDTS-ENQNAPLIVE-AGG------IVGGILGGVISAV----I------TKN---PEVTLKGAVA-----G 175
WP_088865600_1  -------------------------EKGITEEV-GDYIAGKVSE-DSW-----------GTAETIAT----I------IVT---GKIAVETNPS------ 179
WP_048165388_1  ------HYLKLKIRLDSGEYMNLEGIITARYTG-SKYWNEDANG-GIK--------------KGALM----I------IAP---LGLAYAPVEY-----M 172
WP_048055998_1  ------S------------YPVSDVITVVYKDT-RSGSEKAIDY-GST----------AGEIASVAL----L------VVK---GSNPVGWGVF-----I 173
WP_088863688_1  ------D------------RSPSLGEKVETLTK-GGSIAQYVLE-KVAKHAGQLETVASGSLGKATV----V------VSK---FFGVLGWLLT-----A 183
WP_048151737_1  ------N------------YPVSDVITVIPYNG--DETDNAINH-GLT-------LIEIGGFVRTAI----S------SDP---VGILITLGVY-----G 173
WP_048055995_1  ------G------------S----DIVTIHADW-NDVVLDYGGDALIA-------IALAYYAPEAVI----V------SATGWKGAAKKAGVSI-----L 188
WP_055430151_1  ------G------------QIITYDICLLP---------NTVNV-TGY-------EIVGGSSSGAGV----I------LAF---LNIDVGTLLT-----V 170
WP_048150566_1  ------D------------RGIINEVKNFYNDI-KDDIDAGKYT-AVA----------YIFLKGSGK----L------GTK---MAKRAGGILS-----I 172
WP_099209386_1  ------D------------TSPSTGEKIETAVK-VTGIANSILQ-KVAEHAGQLETVASGPLGKVAC----K------VTP---ILNLVSWFLT-----A 174
WP_088886110_1  ---------------------------------------------------------------------------------------------------- 196
WP_068320165_1  ------G------------T----DTVNIKQDN-KDVTVDVGTN-LAL-------IGLDVKAGITII----R------LVRSRETMKALKSAWP-----Y 179
WP_055428943_1  ------G------------Y----GSGRITQSN-KVVTADITIT-LVF-------L----KGSNKVI----V------WTRNA-VEY-----SR-----R 180
WP_014122673_1  ------L------------GDFSLPEKEIAVGI-GSGIAGALTY-FAS----------NEEATLAGA----L------TAA---GIEALSAYVS------ 148
WP_088856137_1  ---------------------------------------------------------------------------------------------------- 177
WP_048152596_1  ------M------------KNPIAGTFRVKKLG-YWDLLKGTLL-YMS----------MLGIGIVFA----F------GPT---WGHSLGAGIF-----S 143
WP_048151739_1  ---------------------------------------------------------------------------------------------------- 162
WP_088865604_1  ---------------------------------------------------------------------------------------------------- 157
WP_088865603_1  ------G------------LDNLETKVRVAYNT-ITTIKNTFED------------IKSGIVGVLET----S------TAK---GIKELAWILK-----Y 126
WP_048150577_1  ------N------------YTVR-SHVILKTS--NTVYGVVVID-IGS-------FIVSFIAGGESL----K------EVI---IAAKAGKRAK-----T 121
WP_055430149_1  ------A------------GDVAKAMYSGAKWV-KDELVGTFTD-VRS--------IAGGAGATKGI----V------KFL---AKKGIEISSE-----S 91
WP_068320157_1  --------------------------IVVHLNN-GITVSR------------------------------------------------------------ 124
WP_014788172_1* --------------------MNLEGIITARYTG-SKYWNSDANA-GIM--------------KGTAM----A------FSP---LLLSGLATFS------ 45
WP_048165379_1  ---------------------------------------------------------------------------------------------------- 109
WP_068320171_1  ------L------------GDFSLPEKEIATGI-GGGIAGALTY-FIT----------NEGATLVEA----L------TAA---GMGSFGRICF------ 86
WP_088865607_1* -----------------------------------------------M--------------KGTAM----T------FSP---LLLSGLATFS------ 20
WP_058939190_1* V-----G------------KGVAKMDISVSVYQ-GTVYADVNQV-------------------------------------------------------- 54
WP_068319946_1  ---------------------------------------------------------------------------------------------------- 83
WP_048150353_1  -------------------------ERGIIKEV-KDYVVNEIEN-DPW-----------GTSSKVAA----M------IIT---GKISVETNPF------ 47
WP_048150344_1* ---------------------------------------------------------------------------------------------------- 80
WP_048055999_1  ---------------------------------------------------------------------------------------------------- 67

WP_058939188_1  LT----------------FL-------------------------------DKFVNGIPNIP------K-N-IVLEIISNT--- 1139
WP_014788798_1  LL----------------IGADIH---------------------------DWLFGP--NPD------T-G-LDNNNVVGG--- 1092
WP_048151755_1  LS---------------------------------------------------FISG--QPI------K-N-FILSKISEHQG- 1085
WP_088181398_1  LT----------------SLPKLG--KA------------A--SEIQWIF-EWGVNG---FP------Q-E-NDNNAVVGG--- 1083
WP_048165390_1  YA----------------GLLLFL--AD------------I--TEEAYFY-GWKQPR-------------D-FDNDGFVG---- 1081
WP_088865610_1  LT----------------FL-------------------------------DKFVNDIPNIP------K-N-IVLEIISNV--- 1077
WP_014012907_1  KN-------------------------------------------------SWPYLVSFFRW------A-W-PKLHN------- 1075
WP_099209391_1  AT----------------A--------------------------------GAVVGLFTNFI------G-G-YVLHNIFGIP-- 1047
WP_088863686_1  YL----------------KL-------------------------------WWDVLHSPVPV------Y-H-GDNNQIIGD--- 1039
WP_068319935_1  FS----------------GIKITM---------------------------KLGFDPWAFVW------N-G-FLKPYILEHT-- 1014
WP_011013125_1  VW----------------EIFKVA---------------------------QAFIRGILKKW---------------------- 996
WP_042692137_1  VT----------------AITSVI-----------------------------------VEV------Y-N-ILKQHLGGDSN- 965
WP_052696221_1  GA----------------MIVEMA--PA------------S--EPVKTIT-QIFLFAKDICQ------G-I-WKEINFGG---- 925
WP_014122995_1  VV----------------SIL------------------------------DFFIDISNFKLQDIPNDS-V-QDNNSVNGG--- 911
WP_088886144_1  IG----------------FGALVL--TT------------V--GYSTY---KGITAPTPTCS------Q-E-SDNNLICGG--- 872
WP_088856136_1  AT----------------FIGGVA--TTEYVVKPLGTFLWD--LWGEY---YWGIHE--PGE------E-E-NDNNSIIGG--- 843
WP_015857921_1  -------------------------------------------------------GVGEF------------------------ 829
WP_014733996_1  ------------------------------------------------------------------------------------ 774
WP_088886109_1  FL------------------VSVG--SA------------N--SNFSDLV-EKVSFLKDFID------F-T-KNAYKSIWG--- 770
WP_088882563_1  ------------------------------------------------------------------------------------ 732
WP_015859709_1  LL----------------LV-------P------------I--VVNGY---RGATAPTPICS------V-E-DDNNLICGG--- 685
WP_014122675_1  VL----------------GL-------G------------I--AIDEY---RTFSAPPRICS------LYE-GDNNLICGG--- 648
WP_014122677_1  AI----------------FIAGVA--TTEYVVKPLGTFLWD--LWGEY---Y---------------------------GE--- 641
WP_056934707_1  ------------------------------------------------------------------------------------ 620
WP_068320152_1  YV----------------GLLFFL--TD------------I--V-------EEAYFYGCKQP------R-D-SDNDGFVG---- 579
WP_082781392_1  IL----------------GLKVNG---------------------------KLGLDPWAFLW------N-G-LIKPYILEHT-- 563
WP_010884703_1  ------------------------------------------------------------------------------------ 558
WP_062388971_1  ------------------------------------------------------------------------------------ 499
WP_074631387_1  AL----------------LVLDIH---------------------------DWLFGP--KPN------T-G-TDNNDVVGG--- 449
WP_010884704_1  ML----------------GK---------------------------------------VAR------V-G-SKFLGFVGS--- 438
WP_056934708_1  --------------------VWIF--TI------------W--LWSAY---ITFTTP--INE------D-V-GDNNLIVGDEQ- 378
WP_048151448_1  GV----------------SFFR------------------W--GWSQI---NN------------------------------- 372
WP_088863691_1  VV----------------QFFR----EA------------W--GWIND------------------------------------ 352
WP_048810998_1  IR----------------QKVSPITNPL------------L--QQISI---NW-------LA------N-W-MKEKLITEWRG- 328
WP_048148049_1  ------------------------------------------------------------------------------------ 327
WP_055428947_1  HT----------------L----------------------------------------LED------Q-E-TPNNNIIGG--- 321
WP_099209381_1  IA----------------DFIR----RA------------Y--GWR-------------------------------------- 316
WP_014122994_1  LV----------------DSESLN--WF------------K--LLIEKVYLRYKHKTGITPD------N-G-SDGNDVTGG--- 305
WP_048165381_1  LT----------------SLPKLG--KA------------A--SEIQWIF-EWGVNG---FS------Q-E-NDNNAVVGG--- 296
WP_048165382_1* ------------------------------------------------------------------------------------ 102
WP_068319940_1  AGTVTGAIVGGIVGFIGGLLLH----EK------------L--GWP-------------------------------------- 257
WP_068320169_1  ------------------------------------------------------------------------------------ 255
WP_068320161_1  VI----------------KPLGTF--IW------------D--LWGNIAW-GFSNPG----A------G-E-NDNNSVAGG--- 254
WP_048055992_1  VI----------------LYKV----KE------------Y--ILELT------------------------------------ 243
WP_074631389_1  VK----------------EKVKPI----------------I--GQISV---NW-------LA------N-W-MKEYAITWWRG- 231
WP_014012910_1  YV----------------GLLLFV--SD------------I--AEEAY---FYGW----RQP------R-D-FDNDGFVG---- 221
WP_015857844_1  VF----------------S--------------------------------GFVGVSIARIA------S-G-LQWNLKYGY--- 219
WP_099209388_1  WA----------------IGTATT--WA------------I--VSLSWKI-YALYEGVTGIS------G-D-GDNNAVVGG--- 215
WP_088865600_1  --------------------VWFL--TI------------Y--TWALI---ESMK-P--LPP------D-TPRDNNLIVGDET- 213
WP_048165388_1  GT----------------VIVEMT--PA------------PKPVKTII---QVFLFVKSIGQ------S-I-WKEINFGG---- 211
WP_048055998_1  LT----------------SLPKAG--KA------------V--SKIQWIF-EWGVSR---FP------E-E-NDNNAVVGG--- 210
WP_088863688_1  RD----------------VI-------------------------------NWLRAP---TP------S-D-GDNNNVIGG--- 206
WP_048151737_1  VT----------------HENDIQ--WI-----------------------LEEIIGNEHFP------Q-E-QDNNNVVGG--- 205
WP_048055995_1  AA----------------FLLN----EA------------K--NWIIT---NW-------S----------------------- 205
WP_055430151_1  GG----------------LLLYFY---------------------------PSIKAPSLLGA------T-E-TDNNLIGGG--- 200
WP_048150566_1  LL----------------IGADLH---------------------------DWLFGP--NPD------K-G-NDNNDVVGG--- 200
WP_099209386_1  RD----------------FI-------------------------------NWLRAP---TP------S-D-GDNNNVIGG--- 197
WP_088886110_1  ------------------------------------------------------------------------------------ 196
WP_068320165_1  VV----------------SFFR------------------W--AWPKL---DS------------------------------- 193
WP_055428943_1  VF----------------GYLDYI----------------T--G---------------------------------------- 190
WP_014122673_1  --------------------LGII--IG------------F--LIYLA---YEGTKP--IPP------S-DSGDNNLIVGDES- 183
WP_088856137_1  ------------------------------------------------------------------------------------ 177
WP_048152596_1  FG----------------LVVYLA-----------------------------------KMA------S-E-FQWILRYGG--- 165
```

```
WP_048151739_1  ------------------------------------------------------------------------------------ 162
WP_088865604_1  ------------------------------------------------------------------------------------ 157
WP_088865603_1  HR----------------LLED-------------------------------------RDV------P-E-YDENNIIGG--- 146
WP_048150577_1  AW----------------EILKMA---------------------------QGFIRGITKRW---------------------- 140
WP_055430149_1  AG----------------MLFGAF--SL------------Y--VKLWI---YVLENP--IPE------N-APGDNNLIIGD--- 128
WP_068320157_1  ------------------------------------------------------------------------------------ 124
WP_014788172_1* ------------------------------------------------------------------------------------ 45
WP_048165379_1  ------------------------------------------------------------------------------------ 109
WP_068320171_1  FG----------------LHSWIL--DL------------L--GL--------------------------------------- 99
WP_088865607_1* ------------------------------------------------------------------------------------ 20
WP_058939190_1* ------------------------------------------------------------------------------------ 54
WP_068319946_1  ------------------------------------------------------------------------------------ 83
WP_048150353_1  --------------------VLLL--TI------------Y--GWALI---ESMK-P--LPS------D-NLGDNNLIVGDEP- 81
WP_048150344_1* ------------------------------------------------------------------------------------ 80
WP_048055999_1  ------------------------------------------------------------------------------------ 67
```

B. arCOG14201 family proteins. Alignments were colored using http://www.bioinformatics.org/sms2/color_align_cons.html server with default amino acid groups with 100% consensus. Conserved histidines are highlighted by red.

```
WP_068320155_1 MDWFRVHNILTFYLPVLIFVSLVYGFITKNSKMLIYSLGYLVAYFSIRLEIHHYQNKLSLHGDRRFVRALIVLDLFAVGFLLPMVLSYTNRANFIRNIIL 100
WP_014012911_1 MDELRVHNILTFYLPLLILTSFMYEFLNKNSRALIYVVGYLIAYLAIRLEIHHYTHKWSAHRDPEVIKILLIYNLLAVGFLLPTLLAYSTKATLIRNIMI 100
WP_068319950_1 MDEARVHNLLTFYLPFLILAGFVYEFLNRNSKALVYILGYLIAYLAIRLEIHHYTHKWSAHRDPEFVKILLIYNLFTAGFLLPTLLAYSTEETIIRNILI 100
WP_014733997_1 MDEARVHNILTFYLPILIFGSFVYGFLNGNSQMLIYAIGYLVTYFAIRLEIHHQEHKWGAHRDTRFVKALVISNLVVVGFLLPTILAHSTKANFNRNLVM 100
WP_048165389_1 MNKVRVHNILTFYLPILIFGSLLYGFLNENSQMLIYAVGYLVAYSAIRLEIHHYHHKWSAHGNTRFVKTLVISDLVVVGFLLPTILAYSTMTDFSRNLMI 100

WP_068320155_1 YLGVGVLIYAMAWKLIEKLTERRLLIISLGLSLVIGMTTGGILEPLIFALLALWTYLVVKHNLVPYAEKNNG- 172
WP_014012911_1 YIIVVLVLYVPISKMIVRLLGRGLFILSFGSSLVIFIITQNILEPTIFALLSLWTYLVLKHDLVAYTQERSVS 173
WP_068319950_1 YLIVAFLMYAPISKMVGKLS-RGLLVLSIVSSFVIFIITQNILEPMIFMLLSLWTYFGTK----TYTKY---- 164
WP_014733997_1 FFIAGAFIYATTWRIIDKLSEDRVGIFLLVLSLLVLIKTKSLLEPLLFALLSLWACLILKHSLAAYATKGL-- 171
WP_048165389_1 FFIVGAFIYVTIWKIVDKISEHGLLVVSLVLSVLILITTKSILEPTIFALLSLWTYLVLKHDLVSYAK----- 168
```

C. Alpha 2-macroglobulin family proteins. Alignments were colored using http://www.bioinformatics.org/sms2/color_align_cons.html server with default amino acid groups with 90% consensus. Conserved motif involved in thiol bond formation is highlighted by red. Proteins were truncated before the alignment, for each accession the starting amino acid of the respective sequence fragment is indicated above the alignment

```
WP_083755773_1    776
WP_013799567_1    1991
WP_018154498_1    307
WP_013866661_1    674
PKL61212_1        910
WP_048104849_1    931
CVK34051_1        946
WP_015591433_1    1290
WP_004075953_1    233
WP_004040593_1    238
WP_042684179_1    763
WP_050459351_1    1367
WP_008096231_1    1354
WP_004967133_1    1367
WP_007275935_1    1367
WP_081457886_1    1272
WP_058566553_1    1217
WP_058826100_1    1217
WP_050459478_1    1217
WP_008095939_1    1217
WP_049967566_1    1217
OLS27023_1        700
OLS16413_1        1041
WP_011499743_1    717
WP_048206237_1    717
WP_015053894_1    719
WP_023844962_1    719
```

```
WP_083755773_1 --------PVNVSNPGSNNLTVKVLSINTI-----DKNISNNEKTVQFNVKGIAADISRVSGIPKN--NVLASGGEYQITAFYNV-SIPETYTSKIVT-- 82
WP_013799567_1 ----------TFNESGTHNITITITPIGTK-----DRNVADNTKTISVTVKEIKADIKEVYGLPYN--NVLQADGEYWIDVYYNV-SVPSYYTIKINT-- 80
WP_018154498_1 ----------TFNESGAHNITITITPIGTK-----DRNMADNTKTISVTVKEAKADIEKVYGLPYN--NILKADGEYWIDVRYNV-SVPSYYTIKINT-- 80
WP_013866661_1 ----------------------VIDPYNHIDEQNENNNKLVRKVIVENPYFKITGK-----YCPTV----VKEGNYFYVGIYYKT-NYKTNYTLYLNYNH 68
PKL61212_1     -------------------------DDVT------DGNVASRSIVIDNYDLELTNQNKEYWGYWYGYNNSVLKNEYFVLGTYFTA-NQAGMVNATVAITY 68
WP_048104849_1 ----------------------ITGDNVT------GNNIASRPIVIDSYELNVTEQNKRYWNAWYGYDRSVLRDEHLWLGTYFTA-NQSGMVDATLAITY 71
CVK34051_1     -----------------------TGDNVT------GNNIASRPIVIDSYELNVTEQNKRYWNAWYGYDRSVLRDEHLWLGTYFTA-NQSGMVDATLAITY 70
WP_015591433_1 -------------------------DTITA-----NNVYTTHPITLKEFHLD-------AWRMYYP--RKVMMGREFWIGSYVNV-TAPGKVNATIKV-- 58
WP_004075953_1 ---------WTPGAEGLYLVNITVTSEDAVK----GRPSDEKSINAIDYQLDIIG------DHAYG-YSSARVDNWFYFYFRVEA-NKAGNVILNVNA-- 77
WP_004040593_1 -----------PATAGTYTLKVNVTSDTGLPVV--GTTTAERSVTARTYTLEVY-------DYVYG-YSRAAVGDWFYLSWYVKP-SESGNVQLNLTA-- 76
WP_042684179_1 --------------------HSNTTDPYP------ANNVYTRTIRVREYSID-------AWYMIYP-TYGVYEGRYFRVGARLNT-TAPGYVNATLTVDP 65
WP_050459351_1 RETAVFTRNHTFATGRHTLVASVADPDAP------RGNADRDVTTSQPYELR---------GRLSA-ADPVQVGRNVTVYGRGYS-NYESNVTANLSL-- 81
WP_008096231_1 RETAVFTRNHTFATGRHTLVASVADPDAP------RGNADRDVTTSQPYELR---------GRLSA-ADPVQVGRNVTVYGRGYS-NYESNVTANLSL-- 81
WP_004967133_1 RESVVLTRNHTFATGRHAVVATVDDPDAP------RGNSDRDTTTSQPYELS---------GRLSA-ADPVQAGQNVTVYGRGYG-NYESNVTANLSL-- 81
WP_007275935_1 RESVVLTRNHTFAAGRHAVVASVDDPDAP------RGNSDGDATTSQPYELS---------GRLSA-ADPVQAGQNVTVYGRGYS-NYESNVTANLSL-- 81
WP_081457886_1 ------RAPHTYNSGGDYTVTATVDDAVPF-----DNNEAAKTIEVKAFDLNLDAE---DISVPTD----ATNGTTFTATARFQT-NYPTTVNATLEL-- 79
```

```
WP_058566553_1 --------THNFATGGDKVVRASVSDGVPF-----DNNESTKDVTVKPYTLSIEDD---DVSVPST----VVNNSTVTVLAEFNT-NYPSNVNATVSL-- 77
WP_058826100_1 --------THRFATGGDKVVSASVSDGVPF-----DNNVSTKDVRVKPYSLSIENG---DVSVPST----VVNNSTVTVLAEFNA-NHPSDVNATVSL-- 77
WP_050459478_1 --------THSFATGGDKVVTASVSDGVPF-----DNNESTKDVRVKPYTLSIDDE---DVSVPST----VVNNSTVTVIAEFNA-NYPSDVNATVSL-- 77
WP_008095939_1 --------THSFATGGDKVVTASVSDGVPF-----DNNESTKNVTVKPYTLSIDDE---DVSVPST----VVNNSTVTVLAEFDA-NYPSDVNATVSL-- 77
WP_049967566_1 --------THSFATGGDKVVTASVSDGVPF-----DNNESTKNVTVKPYTLSIDDE---DVSVPST----VVNNSTVTVLAEFDA-NYPSDVNATVSL-- 77
OLS27023_1     ---------------GDHKFEIKSPDSITRYKIF-VIACNETHFGVEERSIVVKNPIFTSTLNPSD----MTLGDRITLPTVLENLSNEILKDITISINS 80
OLS16413_1     ---------------TGWEQEIVLPDNIGEWVVRLTVTKGGSGAVIKS-SFNTQLPFFVEISKPAI----LMQDDIVGIKGVFYN-YHDELVRANVTI-F 78
WP_011499743_1 ------------DDNGLATLDLNAPDSITKWRLH-AVSSSPEGIGISEAGLTVFQDFFIDPDLPYA----VIRGEEFPVQVQVYN-YLDMPQNVKLTLSG 82
WP_048206237_1 ------------DEDGLASLDLNAPDSITTWRLH-AVSSSPEGIGISEAGLTVFQEFFIDPDLPYA----VIRGEEFPVQVQVYN-YLDKPQEVQLTLSG 82
WP_015053894_1 ------------DEEGLASLELTAPDSITTWRLH-AVSSGPEGIGISEAKLRVFQDFFIDPDLPYS----VIRGEEFPVQIQVYN-YLDREQNVRLTLSG 82
WP_023844962_1 ------------DEDGLASVDLNAPDSITTWRLH-AVSTGPEGIGISESSLTVFQDFFVDPDLPYS----VTRGEEFPVQVQVYN-YLDTEQDVKLTLSG 82

WP_083755773_1 SNG----------IEVIS------NNETKSYVYSLRSEIFTI-RAKNVSKNE-------NISIEIYQ-NEKLLKKETIP------------------LKI 139
WP_013799567_1 TKG----------IQIIG------DSEKTRYTWRYGWTYFKI-KAVDISNDE-------NISIEIFDNNNKLLANKTLTG-----------------IKI 139
WP_018154498_1 TKG----------IQIIG------DSEKTRYTWRHGWTYFKI-KAVDISNNE-------SISIEIFDHNNKLLANETLTG-----------------IKI 139
WP_013866661_1 -------------TIFNSSQWGIKNDTLKGYTYPTGWWSRSI-WIKMKALHNTDNKECINGTLILNTLNGTLIKNLSCC------------------IEV 136
PKL61212_1     PNGTPVDLTDNSVFEFYYYY----PAQYPVYAYDSEWNSVSWYYIQPKQLGTF------NYSITLEA-KGQSAY-VNGT------------------IKV 138
WP_048104849_1 PNGTPD---GESAFEFHHYY----PAQNPVYAYDSEWNSVAWYYVTPRQLGDF------NYSITLKA-RGESAY-VNGT------------------IKV 138
CVK34051_1     PDGTPV---DESAFEFHPYY----PAQNPVYAYDSEWNSVAWYYVTPRQLGDF------NYSITLKA-RGESAY-VNGT------------------IKV 137
WP_015591433_1 PDG----------LLVWNGTGWNDTAKIERFLWGGKWNWFGF-RLKAVKVGEYGNGTTKEINITFTG-RDKVDE-INSSSAVDQVPDY---------KLIV 137
WP_004075953_1 PEG----------FEIYGG-----ENQTK-YLYNSNYNYVYV-HMRTSNPGNYAGD---DFTFNMSA-NGKSDS-IKGRD-----------------IRI 138
WP_004040593_1 PAG----------VEVWG------GANVTQWMSGGYWNYVSY-WMRSSRPGTLAAG---SISLTASA-NGKTASKASTHD-----------------VLI 138
WP_042684179_1 ASG----------ITVY-------HATKRAYTRGSPYDTVYW-LCKGDVAGLY------NMTMTFEA-YGKSAT-ISTTDAQWNVSPYGTRYSPHGPVQI 139
WP_050459351_1 PAG----------LEFAPG-----ETATKY--DTDDRTYFEW-TVVATANRDATY----DLELNLSA-RGENET-ATER-------------------LTV 139
WP_008096231_1 PAG----------LEFAPG-----ETATKY--DTDDRTYFEW-TVVATANRDATY----DLELNLSA-RGENET-ATER-------------------LTV 139
WP_004967133_1 PAG----------LEFADG-----ETATKH--DTDDRTYFEW-TVVAAENRDATY----DLELNLSA-RGETET-ATER-------------------LTV 139
WP_007275935_1 PAG----------LEFAAG-----ETATKH--DTDDRSYFEW-TVVAAANRDATY----DLELNLSA-RGETET-ATER-------------------LTV 139
WP_081457886_1 PDG----------LELVAS-----ENASKDVPASATGGTAAW-KVRVNTTEDVSDA---QVNVTTKA-HGQTSS-ASTT-------------------LNL 140
WP_058566553_1 PSG----------LELVDG-----QTAEKQVRATGNGGSAAW-KVRGNTTADIDDA---QIDVTVEA-FGESDT-ASAT-------------------TNV 138
WP_058826100_1 PAG----------LELAAG-----QSAEKQVRSSAKGGSVAW-KVRGNTTADIEDA---QIDVTVEA-FGQSDT-ASAT-------------------TNV 138
WP_050459478_1 PAG----------LELVDG-----QRAEKQVRSSAKGGSVAW-KVRGNTTADIDDA---QLDVTVEA-FGQSDT-ASAT-------------------TNV 138
WP_008095939_1 PAG----------LELVDG-----QRAEKQVRSSAKGGSVAW-KVRGNTTADIDDA---QLDVTVEA-FGQSDT-ASAT-------------------TNV 138
WP_049967566_1 PAG----------LELVDG-----QRAEKQVRSSAKGGSVAW-KVRGNTTADIDDA---QLDVTVEA-FGQSDT-ASAT-------------------TNV 138
OLS27023_1     NEN----------LKIHSN-----LIQKLKELKANEKVNVYW-DIEASKVGDV------DLSTLLESKYFTEYSELQTP------------------LYI 140
OLS16413_1     VDG----------VEILA------KNEQNVVIPSNFLTQVTW-AVYAKEPGTH------NVTITAVG-TGVITGNIYTDGIQKT-------------IRI 141
WP_011499743_1 AEW----------FELVG------DDVVEVGVDANSVTHVSF-TIRPTRVGVQ------TVELTGQT-TEKADA-IRKT-------------------IIV 139
WP_048206237_1 ADW----------FDLVG------EDVVMVSVDANSVTHASF-TIRPTEVGVQ------EVELTGQT-TEKADA-VRKT-------------------VIV 139
WP_015053894_1 AEW----------FDIVN------DDVQEVTVEANSVGYASF-TIRPTEVGRK------IIEITGQT-TEKADA-VRKD-------------------IIV 139
WP_023844962_1 ANW----------FKIIG------DDVQTVTVDGNGAGYISF-TISPTKVGKQ------IVEITGQT-TERADA-VRKD-------------------IIV 139

WP_083755773_1 MDNPVVIKYSNITTTNESV----TLDFQIFETTEYDIERRLDYIAMIGESGRTLNGIDYLARYP--HGCIEQITSPMVASIYVKEYC-NDNNIFTNVN-- 230
WP_013799567_1 TDHPVEIKYSNITCTNSSA----NLTFMVFNTTKYDVDRHIEYMVMVGEQGRILKGIEYLAEYP--HGCIEQTTSPMVASIYVKKYC-EQNNISTNID-- 230
WP_018154498_1 TDHPVEIKYSNITYTNSSA----NLTFRVFNTTEYDIDRHVEYVVMIGEQGRVLKGIEYLTGYP--HGCIEQTTSPMVASIYVKKYC-DQNNISTTID-- 230
WP_013866661_1 QPQGVIMKSSNITKLNGND--NKSFNLTVYNATPIR-KVKGTIELDGGSDGAIFKGLYFLNKIPNAWGCIEQIASPTLANIYQKKYYYDMNNIDDGGA-L 232
PKL61212_1     REPNVDIKVMNTTMVTNEP--SKTLAFPVFVRTPSE-GQNVKLLLAAGADGRTLQGLTSLIGYP--HGCPEQTMSPAFVALRVKQYYAERGALTPEIN-- 231
WP_048104849_1 REPNVDIKVMNTTLVTDES--SKTMTFPVFNRTPSE-GRNVQILLSAGADGRTLQGLESLIGYP--HGCPEQVMSPALAALRVKQYYEQRGALNDDIN-- 231
CVK34051_1     REPNVDIKVMNTTILVTNET-GDVTMTFPVFNRTPSE-GRNVQILLSAGADGRTLQGLESLIGYP--HGCPEQVMSPALAALRVKQYYEERGALNNKTN-- 231
WP_015591433_1 WVPTIKLRSYNTTILSDTSNEMTFKTIDTSNVTTFS--QKISLVVLAGQEGRVLRGLDYLVQYP--YGCVEQTTSRLLGALHTDWYY-RTKGYPDVYD-- 230
WP_004075953_1 YVPSMEVNSIGTEVVSDGS-SADFIFNTLHTNNTNR--NSTVIVAQLGAQGRTLSGLGYLVGYP--YGCVEQTTSKMMASLNVKNYYLGRDDPPDDFDRI 233
WP_004040593_1 WIPSIQVKSVDSVSADSDS-TFDMTYNTLHTNNTYD--NRTTIVTAQSGARGRTLTGLGYLVRYP--YGCVEQTTSQMLASLNVKNYYLDRPDKPSDFASI 233
WP_042684179_1 IVQTVEVKDDNSTTLQNTQ--SKNMTYQVFNVTTFD--QSGKIDIIAGGLQRMLTGLTYLRGYP--HGCVEQTASPLIAALRIAEYYDNYGYLDAANN-- 231
WP_050459351_1 ERPKTRVVDTAAVNFTGTG--SDSATLTLRNETTYE--HGVTISTHLGTDGRTLAGLDYLVEYP--HACVEQTTSPMLGALYTGQYY-RRNPAPGGYD-- 230
WP_008096231_1 ERPKTRVVDTAAVNFTGTG--SDSATLTLRNETTYE--HGVTISTHLGTDGRTLAGLDYLVEYP--HACVEQTTSPMLGALYTGQYY-RRNPAPGGYD-- 230
WP_004967133_1 ERPKTRVVDTTAVNFTGTG--GDSTTLALRNETTYE--HDLTITTHLGTDGRTLAGLDYLVQYP--HACVEQTTSPMLGALYTGQYY-RRNPAPGGYD-- 230
WP_007275935_1 ERPKTRVVDTTAVNFTGTG--GDSTTLALRNETTYE--HDLTITTHLGTDGRTLAGLDYLVRYP--HACVEQTTSPMLGALYTGQYY-RRNPAPGGYD-- 230
WP_081457886_1 SVPKIRLTDTNATTLSSGS---DNVTVDLSSATTYD--HTVNVTVQAGSQGRTLAGLEYLFTYP--YGCPEQTTSQMMGALRTDQYY-RSGDIPNNYN-- 230
WP_058566553_1 TVPKVRYTTTNATTLESAG-ETQSLALNISDATTYE--HTLNVTVQAGTDGRTLQGLEYLFSYP--YWCVEQTTTQMMSALRTDQYY-RDGDIPSNYD-- 230
WP_058826100_1 TVPKVRYTTTNATTLESAG-ETQSLALNVSDATTYE--HTLNVTVQSGTDGRTLQGLEYLFSYP--YWCVEQTTTQMMSALRTDQYY-RNGDIPSNYN-- 230
WP_050459478_1 TVPKVRYTTTNATTLESAG-ETQSLALNISDATTYE--HTLNVTVQAGTDGRTLQGLEYLFSYP--YWCVEQTTTQMMSALRTDQYY-RDGDIPSNYD-- 230
WP_008095939_1 TVPKVRYTTTNATTLESAG-ETQSLALNISDATTYE--HTLNVTVQSGTDGRTLQGLEYLFSYP--YWCVEQTTTQMMSALRTDQYY-RNGDLPSNYD-- 230
WP_049967566_1 TVPKVRYTTTNATTLESAG-ETQSLALNISDATTYE--HTLNVTVQSGTDGRTLQGLEYLFSYP--YWCVEQTTTQMMSALRTDQYY-RNGDLPSNYD-- 230
OLS27023_1     SPPGIPHPELFRSKLQANKPWIQELEITGEEAFTLG-----IVNFLPGVEEAAIEGVESLAAYP--YGCVEQTSASTIPNAIAYRYLESNDKLTDDIK-- 231
OLS16413_1     EPNGVLRSQKLGGELLNGT---LQLNITQYAETIYQ---KSSLTIAPGLQNLALSSYERLVGYP--YGCVEQTMSRVLPTTLVYQYLLERGELDEDTE-- 231
WP_011499743_1 EAEGVTREIVDNGILKNGT---VELDATLPDAIVPD-SEKVILSFTPSIVAQTISGVDDLLGMP--YGCGEQNMMLFSTDVEVLRYLKATGQQNPEVQ-- 231
WP_048206237_1 EAEGVTREIVDNGILKEGS---VELDTTLPEGIVPD-SEKVLVSFTPSIVAQTITGVDDLLGMP--YGCGEQNMMLFSTDVEVLRYLKATGQQNPEIE-- 231
WP_015053894_1 EPEGATRELVENGILNNGS---VMLDVTLPDGIVPD-SGKVLVSFSPSIVAQTINGVDNLLGMP--YGCGEQNMMLFSTDVEVLRYLEATGQDNPGLK-- 231
WP_023844962_1 EAEGTTREIVDNGVLSSGS---VELDTTLPADIVSD-SEKILVSFTPSIVAQSISGVDDLLGMP--YGCGEQNMMLFSTDVEVLRYLKATGQDNPEVR-- 231

WP_083755773_1 ---FDNMVSSGIIKL--TTEERKPRIFIDGYA-----WGLYGSY---SEKPIHTGYVLYGLTTAKMN---GHVVNNTYLDN---G------------AK 298
WP_013799567_1 ---FDSMVNNGIERL--TTGIRKPKV----ISNNEYAWGLWGSL---YPKPIHTGYVLYGLTTANMS---GYAVDMKYIEN---G------------AR 299
WP_018154498_1 ---FGSNVNNGIERL--TTGTNKPEV----ISSYEYAWGLWGAS---YPNPIHTGYVLYGLTTAKMN---GYDVNMTHIEN---G------------AR 299
WP_013866661_1 LNKTKKNVKIGIDTI--IS----------RQRNDGSWQWWGHG---KSSVYFSAYSIYTMSLAYND--------HDFRGI---SGNASKEKAVIENGVK 305
PKL61212_1     -QTIQQTMQNALDRMNAPNGDNAQQLAGQPYGDRSGGWA-WGKDTWSTPSMFYTFYTNYVITELKKD----MDADPGFWNV---DANMD--GIDLNASAN 320
WP_048104849_1 -KTVRTAMQNALGHM-NATGYN--------AQQASGGWA-WGT--WSTPSMFYTFYPNYVITELL------MDNDPAFWDV---DASMN--GIDLDASAN 307
CVK34051_1     -TTVRTAMQKALDHMNPPDGYNAQQLAGQSYGDGSGGWA-WGTSAWSTPSMFYTFYPNYVITELLRD------NDAAFWNV---DANMS--NIDLNASAN 318
WP_015591433_1 SARVNKSIMMGVQSL--AKGGVR-------GQHDNGSWSMWGWN--PKGDGFYTMYASYGLGRVAEDDVFGYLVKNNLTTTHVYSSSVNKGKFNFNDTIY 319
WP_004075953_1 RNRANESVERGINVL--VNGKLR-------GQHDDGGWSLWGYGSSEASSSSYASYTLAKINRTDED---LRHLLNGKISS---GGTVQPNTVNFDMLIK 318
WP_004040593_1 RSRANESVEGGISVL--VKGALR-------GQHDDGGWSLWGYGESEASSSSYAAYTLARVNESGED---LNRLLTGKISK---NATVNPGTVNFDKLVE 318
WP_042684179_1 -SSLNSTVARGVQRL-----------QYGALKPKANGAWSMWGGP-YSPDSVYFTTYALYALSSVRMS-----TVNYSTIGT------VSVNDTLLDKGAM 308
WP_050459351_1 TGQINASVGAGVARL--TTGQD--------AQHENGSWSMYGND--PDGDVFYTAYALFGTSAVGND-----PVQSARAPV---RADLD--RIDANATVA 308
WP_008096231_1 TGQINASVDAGVARL--TTGQD--------AQHENGSWSMYGND--PDGDVFYTAYALFGTSAVGND-----PVQSARAPV---RADLD--RIDANATVA 308
WP_004967133_1 SDRINDSVGAGVARL--TTGQD--------AQHANGSWSMYGND--PDGDVFYTAYALFGTSAVGND-----PVQSARAPV---RADLD--RIDANATVS 308
WP_007275935_1 SDRINDSVGAGVARL--TTGQD--------AQHANGSWSMYGND--PDGDVFYTAYALFGTSAVGND-----PVQSARAPV---RADLD--GIDANATVS 308
WP_081457886_1 RQRANDTIEIGVEKL--GTNQASSLIWPDLAQNEDGSWSMYGGAFYDEGDLYYTTAALQGVTSVAND-----EVQGTRAEV---SDDIQ--TVNASRSIE 318
WP_058566553_1 RDRANSTISAGISKL---ADEPTPGTYFNISQHDNGAWSMYGNN--PRGDLFYTIYALQGASAVSDD-----AVQSNRTDV---NASLK--RINQSGAVL 315
WP_058826100_1 RDRANSTISAGISKL---ADEPTPGAYFNISQHDNGAWSMYGNN--PRGDLFYTIYALQGASAVSDD-----TVQSNRTDV---NKSLQ--NINQSGAVL 315
WP_050459478_1 RDRSNSTISAGISKL---ADEPTPGAYVNISQHDNGAWSMYGNN--PRGDLFYTIYALQGVSAVSDD-----AVQSSRTDV---NKSLQ--NINQSGAVL 315
WP_008095939_1 RDRANSTISAGISKL---ADEDTPGAYVNISQHDNGAWSMYGNN--PRGDLFYTIYALQGVSAVSDD-----TVQSSRTDV---NKSLQ--NINQSGAVL 315
WP_049967566_1 RDRANSTISAGISKL---ADEDTPGAYVNISQHDNGAWSMYGNN--PRGDLFYTIYALQGVSAVSDD-----TVQSSRTDV---NKSLQ--NINQSGAVL 315
OLS27023_1     -KNLITNMQAGLDRY--TSKF---------LNEKEGGFGLWDGN---NPSVFHTSLAISVIGKISPF---VNVPDDIFTGA----------------RD 296
OLS16413_1     -KELNNMITSGLSRL--YS-----------FRHYDGGFGWWHND---NSNIYMTSYVLYGLNKIVET---GIVVDEEIILQ----------------AAK 295
WP_011499743_1 -AKALTFITTGYQRE--LT-----------FRHSDGSFSAFGES-DGEGSLWLTSFVLSQFSGAR-D---LTTIDENIVRE---A-------------AE 296
WP_048206237_1 -AKAQTYIITGYQRE--LT-----------FRHSDGAFSAFGES-DPEGSLWLTAFVLSQFSGAR-D---VTTIDENILSE---A-------------AD 296
WP_015053894_1 -AKAEMYIVTGYQRQ--LT-----------YRHSDGSFSAFGES-DPEGSLWLTAFVLSQFSGAR-D---LTTIDEGILAE---S-------------AG 296
WP_023844962_1 -AKAEMYIITGYQRE--LT-----------YRHSDGSFSAFGES-DENGSLWLTSFVLSQFSAAR-D---VTTIDENILSE---A-------------AD 296
```

```
WP_083755773_1 WLIT--KQKTD----GS--WE--SDYAY-YMRDD--------FSSNALITISLQQVYNVTD-NNEIKPEIFNATNKSVRYLISQNINN----------- 366
WP_013799567_1 YLVH--SQNSD----GS--WN--TGYTY-YMRDE--------FSSNALITISLAQTYNLTT-NETLKQEIFNATNKSVRYLISQNID------------ 366
WP_018154498_1 YLVN--SQNDD----GS--WN--TGWTY-YMRDE--------YSSNALITISLAQTYNITT-NETLKQEIFNATNKSVRYLISQTVY------------ 366
WP_013866661_1 WLIS--NQSSD----GS--WRAIPGAHE-YIRQT--------IPLTAWTMTSLAEARDATD-NNTLKSEINNSLEKGANFLLKQNLT------------ 374
PKL61212_1     WLIQ--KQKDE----GY--W----SDWG-YISND--------VEWTGFISENLAGEYPYL--NETMKGAVNVSLEKSCAWLLAYDYDST---------- 386
WP_048104849_1 WLIQ--KQKREGVANGS--WS---DWGY--ISND--------VEWTGFISENLKNEYPYL--NKTMKIEVNASLDRSCNWLLTHNYTNE---------- 377
CVK34051_1     WLIG--RQKEE----GR--WS---DWGY--ISND--------VEWTGFISENLANEYPYL--NNTMKGAVNASLRKSCNWLLDQNYEEDYKEK------ 388
WP_015591433_1 WFAQAANRNET---TGAIWWEWPWQGTHW-FFQGH--------LPVTSWIMVSHYQLINEGLINSSAKQVANQTMANVTKYLVSVQNSDGGWNQWGAKDDP 407
WP_004075953_1 WFNK--NHKTE---SGTMYWEWEAPVCHSWTSQS----------NTPFVMLIHSMINETCEVKGEYKDYMLRNMENATKYVVNNINTGRISG-------- 395
WP_004040593_1 WFYLNPDNPSS----GT--WT--WSAGVCHAWTP--------TSNTGFVMLIHDMIRQQGTISEPYATYMTKNMQNATRYFVQNQAADGSWGGS------ 396
WP_042684179_1 WLVS--QQNAD----GS--WT---GRGSDYLRSN--------NTMTAWVLRSLMQCVPYA--DATNQSAINTSISKGFAYLLSTQNSDGGWGDKPTGSTA 387
WP_050459351_1 WLAD--EQAAD----GR--IR---NDKF-YFDDD--------AAMTGFTLVALDQAGPY---NATAAATADQLRGDAASYLVTAQRADGAWGDGS----- 380
WP_008096231_1 WLAD--EQAAD----GR--IR---NDKF-YFDDD--------AAMTGFTLVALDQAGPY---NATAAATADQLRGDAASYLVTAQRADGAWGDGS----- 380
WP_004967133_1 WLAD--EQAAD----GR--IR---NDKF-YFDDD--------AAMTGFTLVALDQAGPY---NATAASTADQLRGDAASYLVAAQRADGAWGDGT----- 380
WP_007275935_1 WLAA--EQAAD----GR--IR---NDKF-YFDDD--------AAMTGFTLVALDQAGPY---NATAAQTADRLRGDAASYLVAAQRADGAWGEGS----- 380
WP_081457886_1 WLSD--HQRAD----GS--VP---ATDY-FLRDQ--------FSSTGYTMVAFDRAEPHL--NSSATQTAEDFRVDAAVFLIENQNSDGSWASDRSQSDI 396
WP_058566553_1 WLAD--KQRND----GS--VR---PNGY-FLRDR--------TSATAYTTVAIDRAGDDL--NATAQTAADEFSVDAAVHLIERQNSDGSWEAG------ 387
WP_058826100_1 WLAD--KQRND----GS--VR---PSGY-FLRDD--------ASATAYTTVAIDRAGDDL--NATAQTAANEFSVDAAVYLIEQQNSDGSWEAG------ 387
WP_050459478_1 WLAD--KQRNN----GR--VR---PSGY-FLRDD--------TSATAYTTVAIDRAGDDL--NATAQTAANEFSVDAAVYLIEQQNGDGSWEAG------ 387
WP_008095939_1 WLAD--KQRNN----GR--VR---PSGY-FLRDD--------TSATAYTTVAIDRAGDDL--NATAQTAANEFSVDAAVYLIEQQNGDGSWEAG------ 387
WP_049967566_1 WLAD--KQRNN----GR--VR---PSGY-FLRDD--------TSATAYTTVAIDRAGDDL--NATAQTAANEFSVDAAVYLIEQQNGDGSWEAG------ 387
OLS27023_1     FLSS--KQNED----GS--YE---SSKG--VHQN--------FPAT-LSNLSMTAYVAHS------QALGGVTDQPAIEWLLKQIETEDLKS-------- 360
OLS16413_1     FLLD--KQSSS----GK--WT---SDYW-NIND---------LPFTAFVTRGLSTIA-----NKSLLTDYGTVMSLAHDFIETQWVN------------- 356
WP_011499743_1 WIGS--YQKED----GS--WE---AVGF-VIHEDMMGGVSGTYALTAYVTLALDEYGYAA----------PIVMENARSYLEAELDK------------- 361
WP_048206237_1 WIIS--HQKED----GS--WE---SVGF-VIHQDMMGGLSGTYALTAYVTLALDEYGYAP----------DEVMDSALTYLEDNLDA------------- 361
WP_015053894_1 WIES--HQLAD----GS--WE---PVGF-VIHQDMMGGVSGTYALTAYVTLALDDYGSAN----------PVVMADARKYLEDNLAT------------- 361
WP_023844962_1 WIES--YQQED----GS--WE---QVGF-VIHQDMMGGVSGTYALTAYTTLALEEYGSAN----------PVVMSKAQQYLEDNLAG------------- 361

WP_083755773_1 --DDDIYKLGYKAWVLSNAYNMG-----INDSDVFNALNDTVVKLSAWADENQ--------EKSVT---YG--FTGTT---SYSIYG--------VASES 435
WP_013799567_1 --KNNIYDLGFKAWALSEAYNMG-----INDSEVKDALNQTIKDIGNWINEHN--------NANVI---SL--FRGTS---SYSVYG--------VASES 435
WP_018154498_1 --ENNIYDLGFKAWALSEAYNMG-----INDSEVKEALNETIIAIGNWKNEHK--------NDEAI---SL--FRGTS---SYSVYG--------VASES 435
WP_013866661_1 ----KTEEISMSMIALNR------------TGFNWANISGNMSSLATKLKNKQ--------SSDGH--WNPDLWSGWA---YWEP------------ET 432
PKL61212_1     --NADTQALSYAIIGLAAIRDH--------GIGDAGEIDAEIDALKTRLLGKM--------VTSGLESYWD----DQN---LWDTY-----------EP 449
WP_048104849_1 ----DTQALSYAILGLVAIRDHG-------GIGDAEAINETVDDLKGELLKKR--------DRVGS--YWE----DSY---GWNTY-----------EP 437
CVK34051_1     --DTGTLALSYAILGLVAIRDH--------GIGDAEEINITVDALKGQLYNKS--------TLSGSEIYWS----DSS---RWDTY-----------ES 451
WP_015591433_1 AKPSDAISTALSVWGLKLYGTPS-------DEVNRSQIENAIEKGVAWLLNNTYSAA----PDKVY--WHH--PLASP---WWDNYG--------RKSEA 481
WP_004075953_1 --DDKNMATALSLWGLEAFGITS-------ADVSQEALDTAKTTAREYLIEAQS-------KEDGS--WST---GSKY---GWSNTG--------RITES 463
WP_004040593_1 --SDKAMATALGLWGLQSYGTNS-------DDVTQAQIDTAKANATAWLVANQ--------NADGS--WDA---DSSY---GWYSNG--------RTTES 463
WP_042684179_1 YTQSNAYTTGLVLYLLATSNNNT------NWVNNTTQVQNAITNATAWLVAHQLSSDVSRYAKKGS--FYP--VSRDVAGHSYSRVG--------VYTEA 469
WP_050459351_1 --TPNTMSTALAVWGLEA------------SGVETAPVRTAIDDGRAWLLDNQ--------ADSGA--WSQ---RRSA---SWSDVG--------TTSET 442
WP_008096231_1 --TPNTMSTALAVWGLEA------------SGVETAPVRTAIDDGRAWLLDNQ--------ADSGA--WSQ---RRSA---SWSDVG--------TSSET 442
WP_004967133_1 --DPNTMSTALAVWGLEA------------SGVETPAVQTAIDDGRTWLLDAQ--------AESGA--WTE---RRSA---SWSDVG--------TTSET 442
WP_007275935_1 --TPNTMSTALAVWGLEA------------SGVETAAVQTAIDDGRTWLLDAQ--------ADGGA--WTE---RRSA---SWSDVG--------TTSET 442
WP_081457886_1 --SQKPQATALALRGLATVNESAALRQRVNQQTDT-DLSTAIDNGSQWLIDNQ--------NADGS--WDA--YRNSF---FWSSNG--------EVTRA 470
WP_058566553_1 --SREAQATAYAVQALAAVNDSDTLRTQVNSELSSGSVSMALDSGTQWLVANQ--------HDDGS--WTG--YTNSP---YWSNTG--------EKARA 462
WP_058826100_1 --TKEAQATAYAVQALAAVNDSDTLRNQVNNELSSGNVSMALDSGVEWLVANQ--------QADGS--WAG--YTNSP---YWSNVG--------EKARA 462
WP_050459478_1 --SKQAQATAYAVQALAAVNDSDTLRTQVNSELSSGNVSMALDSGVQWLAANQ--------REDGS--WAG--YTNSP---YWSNTG--------EKARA 462
WP_008095939_1 --SREAQATAYAVQALAAVNDSDTLWTQVNNELSSGNVSMALDSGVQWLAANQ--------REDGS--WAG--YTNSP---YWSNTG--------EKARA 462
WP_049967566_1 --SREAQATAYAVQALAAVNDSDTLRTQVNNELSSGNVSMALDSGVQWLVANQ--------QEDGS--WAG--YTNSP---YWSNVG--------EKARA 462
OLS27023_1     --DPTVIALLLDSIAMLK------------DKLPKEIVDK-IPKVTELLLTNIEE------NDKGS--YWS----------KGSALS--------SEVET 419
OLS16413_1     --SSYVAALTLESFY---------------NTPYSTFLESNLIQHLQNEYVLD--------PKLGI--YWT------DLKKGYKALG--------KDIET 415
WP_011499743_1 --QDDPYALAIGTLAL--------------QKLESDRADESMEKLLE--LAKE--------DENGV--YWG--YDDVM-PEPYEYGGYGFHPISSKNVET 430
WP_048206237_1 --QDDPYTLAIGTLAL--------------EKLESERADEAREKLLA--MAKE--------DENGM--YWG--YDDVV-PEPYEYGGYGIYPVSSKNVET 430
WP_015053894_1 --QKDPYALAIGTLAL--------------QKLDSPFADKALNNLLA--ISKQ--------DDHGT--YWG--YGDGPVPMPYEHHGYDFMVPSSKNVET 431
WP_023844962_1 --QDDPYALAIGTLAL--------------QKLDSSMADEALIELLA--ISKQ--------DEDGM--YWG--YGDGDVRLPYEHGGYGFIAPSSKNVET 431

WP_083755773_1 VAISSIGLHNSKD--------IINNDA------YSKLNSHLVSIYSIY---GGSGWGSTKSTGYALRAITLE---RPEAVDNMTIDVYVN--ST-----L 508
WP_013799567_1 IAVASIGCQKAKD--------IIDNDE------YITLNNMLINIYSRY---GGSGWGSTKSTGLALRALTESG--NATNTNNIQITVKVD--GN-----K 509
WP_018154498_1 IAVASIGCQKANG--------IINNSE------YGTLNSMLINIYSRY---GGSGWGSTKSTGLALRALTESG--TATNTNNIQITVKVD--GN-----E 509
WP_013866661_1 TGYSILALHYA-G--------YPNNDS-----NVSAGMDYLLTHFNPG---WG--WGSTKQTATAVQTIVII---NPNVELNSNVSVYVD--GN-----L 503
PKL61212_1     TASAVLALHEA-G--------VDATEL-------SGGVSHLIGNRAGRSYSGG--WGSTRTSAAVINTLTNVV--PPANMD-FTVDVEIKS-PAGATVWS 527
WP_048104849_1 TASAILALNKA-G--------ISADEL-------SGGISHLIGNRAGRSYSGG--WGSTRTSAAVINTLTNVV--PQKPVA-FTVDAEIVR-GDGTSVWS 515
CVK34051_1     TASAVLALNRS-G--------MSADEL-------SGGITHLIGNRAGRSYSGG--WGSTRTSAAVINTLTGVV--PQKPGV-FAVDVEIKRVGDDTPVLP 530
WP_015591433_1 TAYAIIALNESRSMNNLNFTVLDSNNA-----TIAKAVNYLVSVYRGH---GS--FGYTASTQAALHALTLL---QVVPMNETTVTINID--NGA----V 562
WP_004075953_1 TAYAILALNAT-G--------ITGDNT-----TITDGINWLVNQYENG---GK--WGYTWASQAAIDALITC---QGSVKTSGTVSVDID--SGSIV--K 537
WP_004040593_1 TAYTVLALNAT-G--------IKADDT-----TIQKGVNWLINQYESG---GS--WGYTWATQAAIDALIHC---QPISVSSGTVDVSID--GTHI---- 535
WP_042684179_1 TAYALLGLNAT-G--------LDSSNT-----TIADGRKYLYGIYQSD---GS--WGYTKSSAVVINALTKTI--IPGGTIDLNITVGID--GT-----N 541
WP_050459351_1 TAYALMALNAT-G--------LENTNA-----SVQAGQTYLVTVYDDR---GS--WGYTRATALAINTLTLG---QGGSTPTHDVTVTFG--TESTPALV 519
WP_008096231_1 TAYALMALNAT-G--------LENTNA-----SVQAGQTYLVTVYDDR---GS--WGYTRATALAINTLTLG---QGGSNPTHDVTVTFG--TESTPALV 519
WP_004967133_1 TAYALMALNAT-G--------LENTNA-----SVQDGQTYLVTVYDDR---GS--WGYTRATALAINTLTLG---QGGSNPTHDVTVAFG--TESNPALV 519
WP_007275935_1 TAYALMALNAT-G--------LENTNA-----SVQDGQTYLVTVYDDR---GS--WGYTRATALAINTLTLG---QGGANPTHDVTVAFG--TESNPALV 519
WP_081457886_1 TGHSILALNETQG--------VTTQGTSDAADSVEDATDYLVGVYDSD---GS--FGNTRATGVAINALTTA---DQRGVTPQTVDVTVG--G------V 546
WP_058566553_1 TGNAIIALNAAEG--------YTAANN----DTVSDGVGYLVGIYQGG---GS--FGNTRATGVAIDALTTA---DQRNAGSQTVTVEIN--ST-----V 535
WP_058826100_1 TGNAIIALNDAAG--------YTATSN----GTVSDGVGYLVGIYQGG---GS--FGNTRATGVAIDALTTA---DQRNAGSQTVTIEIN--SS-----V 535
WP_050459478_1 TGNAIIALNAAEG--------YTAASN----DTVSDGVGYLVSIYQGG---GS--FGNTRATGVAIDALTTA---DQRNAGSQTVTVEIN--ST-----V 535
WP_008095939_1 TGNAIIALNAAEG--------YTAASN----DTVSDGVGYLVSIYQGG---GS--FGNTRATGVAIDALTTA---DQRNAGAQTVTVEIN--ST-----F 535
WP_049967566_1 TGNAIIALNAAEG--------YTAAGN----DTVSDGVGYLVSIYQGG---GS--FGNTRATGVAIDALTTA---DQRNAGAQTVTVEIN--ST-----V 535
OLS27023_1     TSYALAALAHTEN--------YPAEIL----NIFETGANFLLNTRSSS---G---WFTTRDTLWATLALGEIASKMEQNAVNGTLNVKLN--GE-----V 494
OLS16413_1     TSTALRVLATI-D--------YGTNAI-----LIQEAIQWIVSKQQR----WG--WYSTADTSAAIQAFISLSEYESKAIL-GDVDVIIN--GN-----N 487
WP_011499743_1 TAYATLAL-------------IETNDP-----RASSSLKWIAAQRNSN---GG--FSSTQDTVMAFRALMTA---AAVAGRDVDATVTVS--GDGV---E 499
WP_048206237_1 TAYATLAL-------------IEVNDP-----AASSSLKWIAAQRNSN---GG--FSSTQDTVMAFRALMTA---AATAGRDVDATITVT--GDGE---E 499
WP_015053894_1 TAYATLAL-------------IDARNP-----VASSSLKWISAQRNSN---GG--FSSTQDTVMAFRALMSA---AASAGRDVDATVKVT--ADNT---T 500
WP_023844962_1 TAYATLAL-------------IEANNP-----SATSSLKWIAAQRNSN---GG--FSSTQDTVMAFRALMSA---AASAGRDIDATVHVI--ADGT---E 500

WP_083755773_1 IDSIILNNSNPKYVGKY-INDSHLT-----PGS-------------HTISFNFSKN-AKIVCGGLISQTT 558
WP_013799567_1 IDTITVNATNPKFIGKY-YNNTLLT-----SGE-------------HNITFEFSGD-SKILCGVLMSQTT 559
WP_018154498_1 INTITVNATNPKFVGKY-YNNTLLT-----SGK-------------HEIAFEFSGS-SKILCGALVSQTT 559
WP_013866661_1 ITNVSLTKKTPHAVVNI-PSSNLAI------GN-------------HKITLKSTGS-GKVVASGLIKQWV 552
PKL61212_1     RNDIPFNQASFSFIHEL-TEEELDTLYGFGATN------GTATVVISGKTDADPDNVAKLSVSIDSFEQV 590
WP_048104849_1 CMGIEFNETSFGFERTL-SIDELNILYGSSVPD------DTAKVIISNKNDAASDNPGKLSVSIDSFEQV 578
CVK34051_1     QKRIEFNETSFGFEHTL-LIGELDALYGSGELN------DTAKVIISNKSDAGAKNPSKLSISIDSFEQV 593
WP_015591433_1 VKDVTVNSTNPKVIIVL-TPDELNAVNSNGTSE-------NTNRRIHTVNVTRVSGDSLVIVSVENEQTV 624
WP_004075953_1 NVALSDAAGTQRVEVTL-TEDEMKTLMSGGTRTRGITGNPFGEVRYHTVDVTLTGD-GPVIVSVENTQWA 605
WP_004040593_1 -CTLNVDATNPKDEYTL-TADQMAAMMANGTETR--YPVPYSTVKEHLVDVDRTGGTGTILVSVENSQRA 601
```

```
WP_042684179_1 VTTVHLTNTNPKATIPL-WGERTNSSSPLGAGN-------------HTVTVYQTGT-GTSLVGVSVSQTV 596
WP_050459351_1 TKTIDVDSTDALDSVEL-TDSELQTLRDNADGP-------------ITVQVDTTGT-GVLVVGLENDQLV 574
WP_008096231_1 TKTIDVDSTDALDSVEL-TESELQTLRDNADGP-------------ITVQVDTTGT-GVLVVGLENDQLV 574
WP_004967133_1 TKTIDVDSTDALDSVEL-TESELQTLRDNADGP-------------ITVQVDTTGT-GVLVVGFENDQLV 574
WP_007275935_1 TKTIDVDSTDALDSVEL-TESELQTLRDNADGP-------------ITVQVDTTGT-GVLVVGIENDQLV 574
WP_081457886_1 TKTASVDSSTTTDTVTF-STSELQQFRDEGTGK-------------IELEAQNSDS--LVVIGVESVQVV 600
WP_058566553_1 SKNVTVDGNTPTAVVSF-TTDELETLRQ--TGS-------------ITLTAENSTR---VVVGAESEQLV 586
WP_058826100_1 TKEVSVDGNTPTATVTF-TTDELDTLRE--TGT-------------ITLTVDNSTR---VVVGAESEQLV 586
WP_050459478_1 TKDVTVDGNTPTATVSF-TTDELETLRE--TGD-------------ITLTIENSTR---VVVGAESEQLV 586
WP_008095939_1 TEDVTVDGNTPTATVSF-TTDELETLRE--TGT-------------ITLTVENSTQ-TPVVVGAESEQLV 588
WP_049967566_1 TKDVTVDGNTPTATVSF-TTDELETLRE--TGT-------------ITLTIENSTR---VVVGAESEQLV 586
OLS27023_1     IASEFINKEN-KYYKIYDVRNIFLDKFT--TGS-------------NKVEVFLEGE-GTGHIALEIRKWY 547
OLS16413_1     IASIKYLNDTTPEVSTYKLDDFMK------DGE-------------NFLNIVRQGS-GKVLYYLTTEQTL 537
WP_011499743_1 IKGLRITSENYDVVNIIEVPEGVEVLEMELSGS-------------GELNYQLVRRFNVILPEIIEHEQI 556
WP_048206237_1 LKSLRVTSENYDVVNIIEVPDGVESVNMTLEGS-------------GELNYQLVRKFNVILPEIIEHEEI 556
WP_015053894_1 VRSIDVNSRNFDVVQIVEIPENTSQARLEMEGT-------------GELSYQLVRRFNVILPEVTPQKEI 557
WP_023844962_1 ISSVEVNQQNFDVVQIIEIPDDVSTVTLSLEGT-------------GDLNYQLVRRFNVILPDVVEQNEI 557
```
