# Supplementary figures and images for: A Unique Gene Module in Thermococcales Archaea Centered on a Hypervariable Protein Containing Immunoglobulin Domains
Source: Front Microbiol. 2021 Aug 18;12:721392. doi: 10.3389/fmicb.2021.721392 (PMC8416519; doi:10.3389/fmicb.2021.721392)

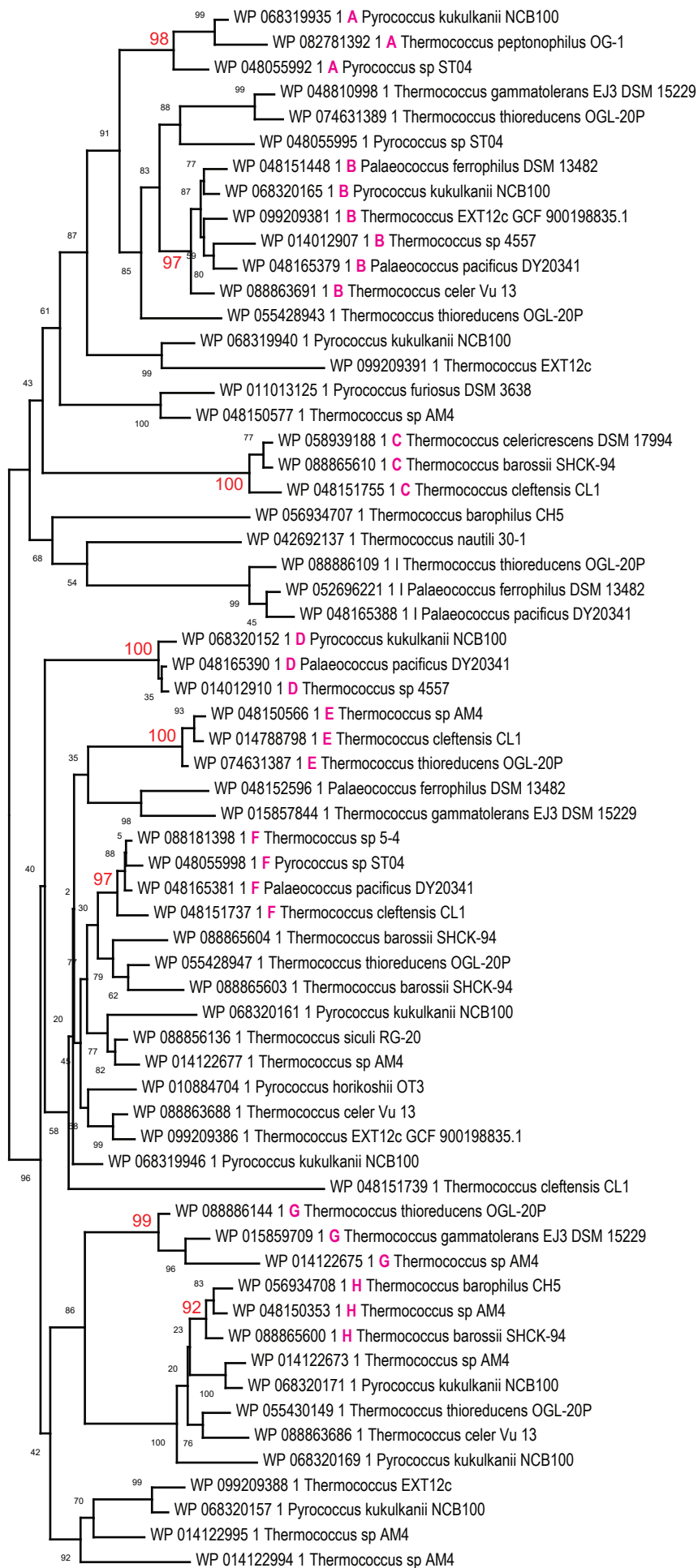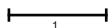

Supplement: Supplementary Figure 2 — Phylogenetic tree of the ∼200 last amino acids of Ig-like domain containing proteins associated with thermococcal hypervariable system. [file Image_2.pdf]

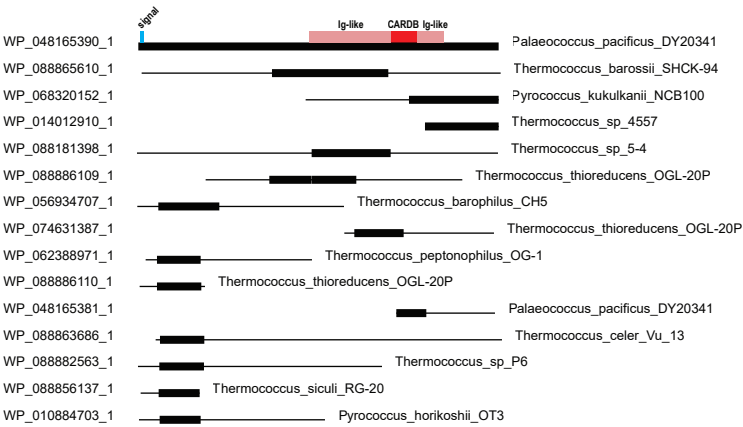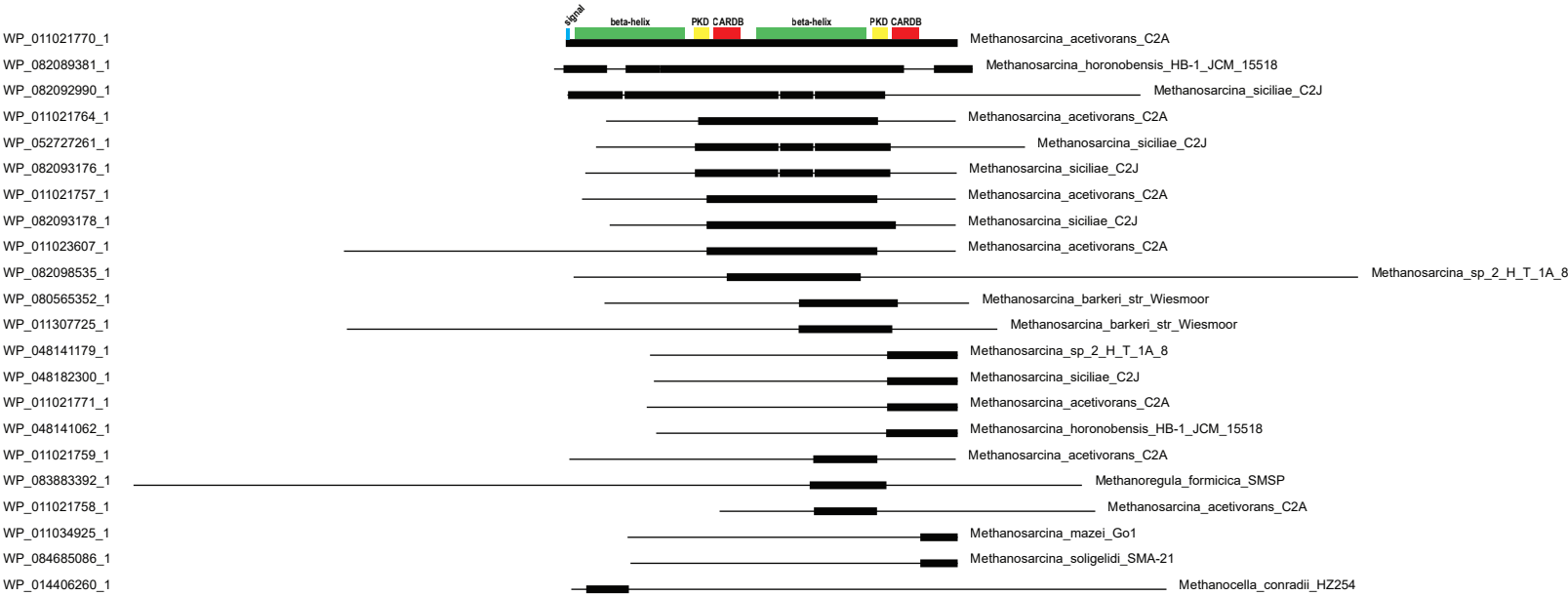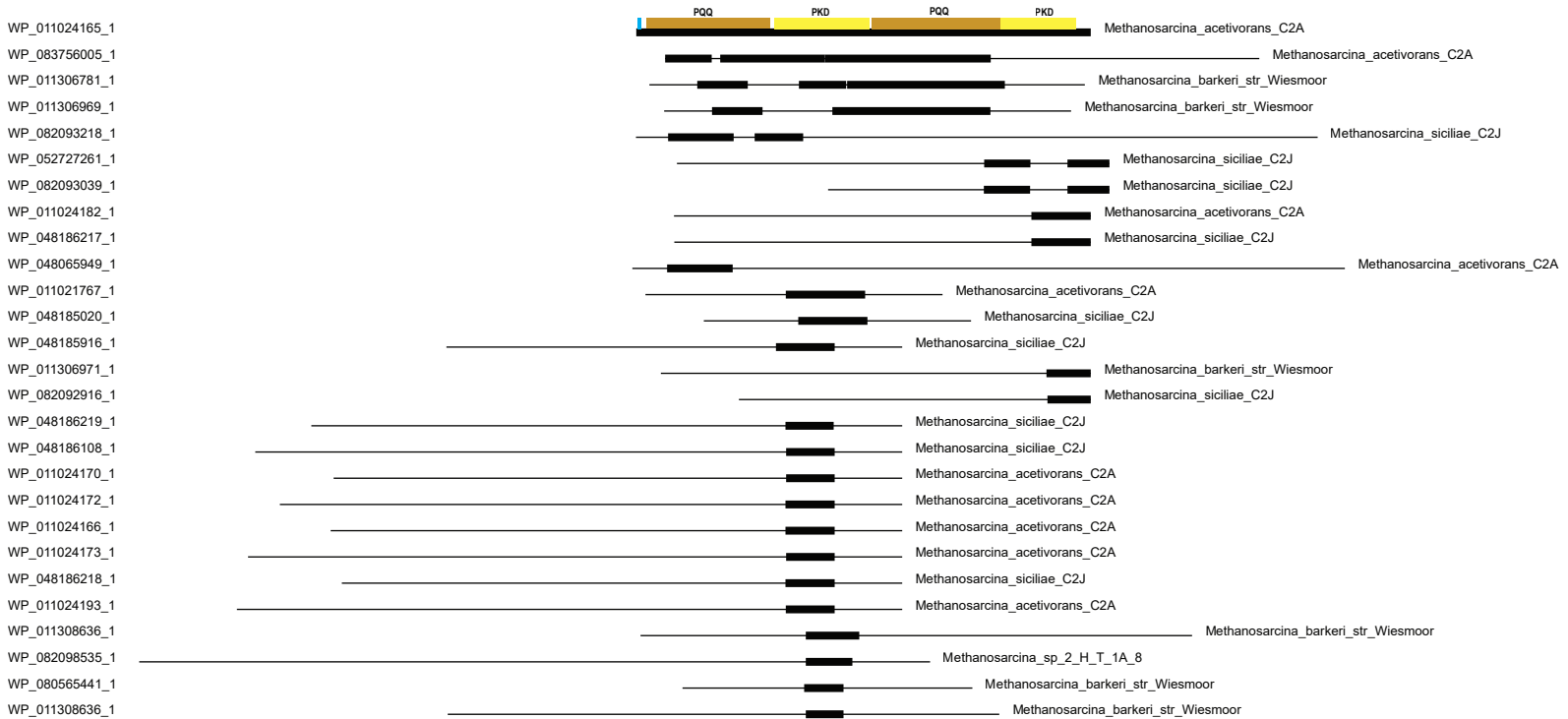

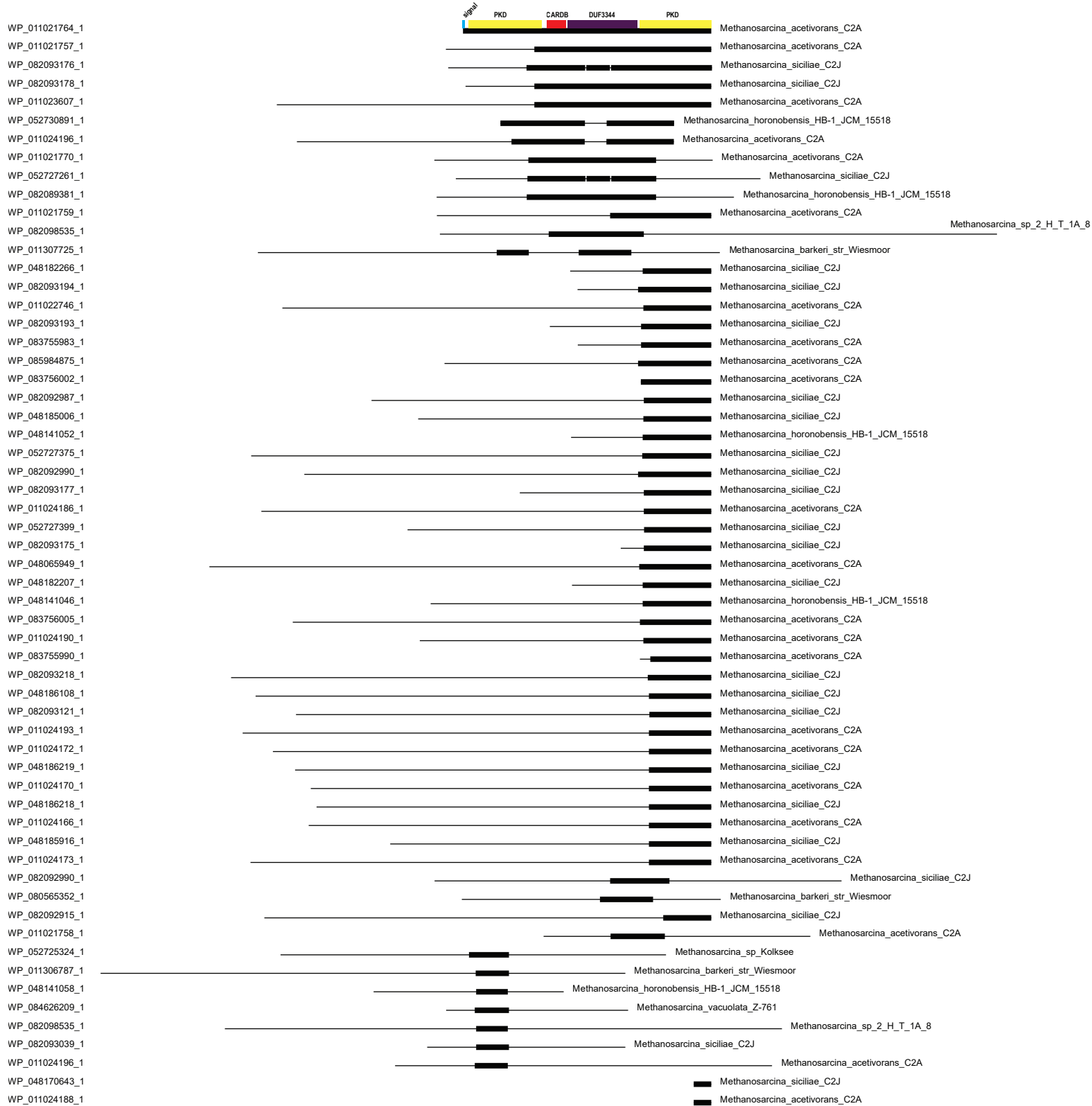

Supplement: Supplementary Figure 3 — Mosaic proteins containing Ig-like domain. In each scheme is shown high similarity region for each blast hit which is mapped on the query protein (first line). The length of the line is proportional for respective protein length. For query sequence the domain organization is schematically shown above the respective line. The BLAST search was performed with the parameters modified as follows: “-seg no -comp_based_stats 0 -dbsize 20000000 -gapopen 32767 -gapextend 32767.” This modification allows to identify ungapped regions of a very high similarity, which might be suggestive of intraprotein recombination (or mosaicism). [file Image_3.pdf]
